# Cubane Electrochemistry: Direct Conversion of Cubane Carboxylic Acids to Alkoxy Cubanes Using the Hofer–Moest Reaction under Flow Conditions

**DOI:** 10.1002/chem.201904479

**Published:** 2019-11-07

**Authors:** Diego E. Collin, Ana A. Folgueiras‐Amador, Derek Pletcher, Mark E. Light, Bruno Linclau, Richard C. D. Brown

**Affiliations:** ^1^ School of Chemistry University of Southampton Highfield, Southampton SO17 1BJ UK

**Keywords:** bioisostere, cubanes, electrosynthesis, flow electrochemistry, oxidative decarboxylation

## Abstract

The highly strained cubane system is of great interest as a scaffold and rigid linker in both pharmaceutical and materials chemistry. The first electrochemical functionalisation of cubane by oxidative decarboxylative ether formation (Hofer–Moest reaction) was demonstrated. The mild conditions are compatible with the presence of other oxidisable functional groups, and the use of flow electrochemical conditions allows straightforward upscaling.

## Graphical abstract

**Just go with the flow**: Oxidative anodic decarboxylation of cubane carboxylic acids leading to cubyl ethers is described. This first direct electrochemical functionalisation at the cubane system is chemoselective and scalable, with yields of up to 60 % (see scheme).

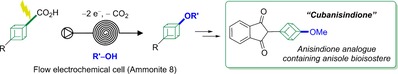

First synthesised in the 1960s^1^ and originally proposed as 3D benzene bioisostere in the 1990s,^2^ it is only in the past decade that cubanes have gained real traction in medicinal chemistry.^3^, ^4^ Substituting a phenyl ring by a cubyl unit can lead to improved physical and biological properties.^5^ In addition, 1,4‐disubstituted cubanes have applications as non‐aromatic rigid spacers in organic materials and polymers.^6^

To date, the only practical large‐scale synthesis of cubane leads to 1,4‐cubanedicarboxylic acid,^1^, ^7^ and while cubane functionalisation has been extensively investigated,^8^ in practice it has relied heavily on interconversions of the carboxylic acid functional group or indirect decarboxylative functionalisation reactions (Scheme 1). To the best of our knowledge, the only direct decarboxylative cubane C−C bond formation is a single example of Pb(OAc)_4_‐mediated Kochi coupling reported by Moriarty (Scheme 1).^9^ More generally, redox‐active esters (RAEs), such as the *N*‐phthalimido ester **3**, have proven to be useful intermediates in Fe‐catalysed cubyl‐aryl coupling to give **4**.^10^, ^11^ Baran and co‐workers also showed RAEs as intermediates for Giese radical reactions (not shown),^12^ and they have been employed for decarboxylative cubyl‐heteroatom bond formation (e.g., decarboxylative borylation, **3**→**5**).^13^, ^14^ Other examples of decarboxylative radical‐based methods have been reported.^15^, ^16^

**Scheme 1:**
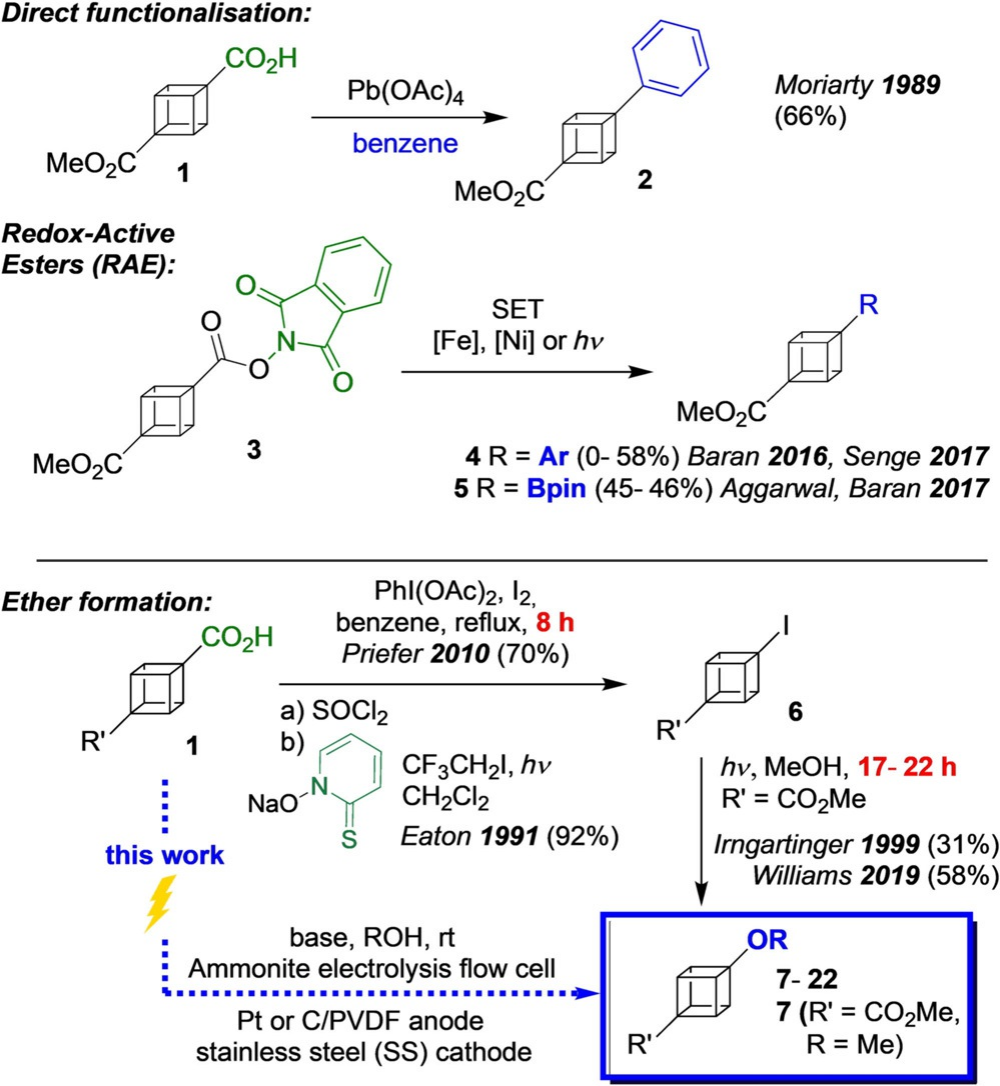
Decarboxylative cubane functionalisation.

The synthesis of alkoxy cubanes **7** has been reported from iodides **6**, which are again accessed from cubane carboxylic acids,^17^, ^18^, ^19^ or their pyridyl esters.^20^, ^21^ Eaton and co‐workers reported that photolysis of a dilute (0.03 m) solution of 1,4‐diiodocubane in methanol afforded 1‐iodo‐4‐methoxycubane in 10 % yield.^22^ Subsequently, Irngartinger^23^ and later Williams and co‐workers21a improved the efficiency with photolysis yields up to 58 %, providing access to methoxycubanes as anisole isosteres.

The viability of processes proceeding via the intermediacy of cubyl radicals is amply supported by the examples described above. Additionally, the possibility of forming cubyl cations is inferred from solvolyses of corresponding iodide or triflate.^24^ This precedent, together with our ongoing interest in the area of electrosynthetic transformations,^25^ led us to explore the application of electrochemistry as a direct approach to generate these reactive intermediates^26^ from cubanecarboxylic acids. In Kolbe electrolysis, anodic oxidation of carboxylic acid salts initially forms alkyl radicals, which may undergo coupling to give symmetrical or unsymmetrical products. The initially formed radicals may be further oxidised to give carbocations, which can react with a nucleophile to give, for example, alcohols, ethers, and esters.^27^ This non‐Kolbe carbocation pathway, known as the Hofer–Moest reaction, has very recently been optimised by Baran, Blackmond and co‐workers for the synthesis of hindered ethers through carbocation formation under batch electrolysis conditions. Despite reporting an impressive array of ether products (>80), cubane carboxylic acid was reported to be a challenging substrate, which did not give the Hofer–Moest product under their conditions.^28^ Herein, we report direct anodic functionalisation from the commercially available carboxylic acid **1** under flow conditions as a convenient method for cubane to give a variety of ethers. Furthermore, the scalability of the process was demonstrated.

Our electrochemical investigations of cubanecarboxylic acids were facilitated through the application of flow electrolysis cells.^29^ Interest in laboratory electrosynthesis in continuous flow has grown significantly over the last decade^30^ and presents some advantages compared to batch electrolysis. In flow cells, the electrode surface to reactor volume ratio is much higher than in batch, facilitating higher productivity and more efficient mass transfer. Moreover, the interelectrode gap is usually small (typically between 0.1‐1 mm), allowing a low loading or even elimination of supporting electrolyte.^31^

For initial exploration of the reactivity of the cubane moiety towards electrolysis, and whether radical or carbocation reactivity would be observed, cubane carboxylic acid **1** was subjected to typical Kolbe‐type electrolysis conditions in an undivided flow reactor.^32^ Partial deprotonation using KOH as base and excess current (6.2 F) was applied in MeOH, using Pt as anode material.25a Without supporting electrolyte, preliminary experiments established that 0.5 equivalents of base was required to reach the desired cell current and methyl 4‐methoxy‐1‐cubanecarboxylate (**7**) was obtained in 14 % as the major product (Table 1, entry 1). In addition, starting material **1** (50 %) and a small amount of the hydrogenolysis product **10** (<5 %) were observed. This promising initial result showed that the cubane ring itself is clearly compatible with anodic oxidation, and that processes proceeding through cubane carbocation formation are viable.

**Table 1:** Selected electrolysis optimisation conditions.

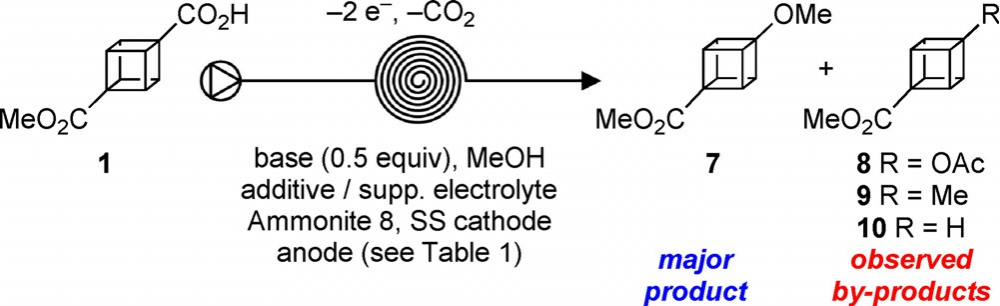						
Entry	Anode	Conditions	Flow rate [mL min^−1^]	Current [mA] (charge [F])	RSM [%]^[a]^	Yield [%]^[b]^
1	Pt	KOH	0.2	200 (6.2)	50	14
2^[c]^	Pt	KOH, AcOH	0.2	200 (6.2)	none	40
3	Pt	KOH, AcOH	0.4	400 (6.2)	none	36^[d]^
4^[c]^	Pt	Et_3_N, AcOH	0.2	200 (6.2)	none	44
5	Pt	Et_3_N	0.2	200 (6.2)	82	8
6^[c]^	C/PVDF	Et_3_N, AcOH	0.2	200 (6.2)	none	12
7^[c]^	C/PVDF	Et_3_N, AcOH	0.5	200 (2.5)	none	44
8	C/PVDF	Et_3_N	0.5	200 (2.5)	none	52
9	C/PVDF	Et_3_N	0.25	200 (5.0)	none	24
10^[e]^	C/PVDF	Et_3_N, NaClO_4_	0.5	200 (2.5)	32	4
11^[e]^	C/PVDF	Et_3_N, Et_4_NBF_4_	0.5	200 (2.5)	20	12
12	C/PVDF	DBU	0.5	200 (2.5)	none	34
13^[f]^	C/PVDF	Et_3_N	0.5	200 (2.5)	22	36

General conditions: 0.25 mmol of **1**, MeOH (2.5 mL), *c=*0.1 M. [a] Determined by using calibrated GC. [b] Yield of **7** determined using calibrated GC. [c] AcOH (1.0 equiv). [d] Isolated yield: 1.03 g of **1** gave 350 mg of **7**. [e] 5 mm of supporting electrolyte. [f] Et_3_N (0.75 equiv). RSM=remaining starting material.

Optimisation efforts continued using Pt and carbon‐based anodes (see the Supporting Information for details). For the Pt anode, it was observed that the presence of supporting electrolytes, such as Et_4_NBF_4_ and perchlorate salts (from NaClO_4_, LiClO_4_) supressed decarboxylation of cubane, with only traces of **7** observed. Interestingly, while basic conditions are required to form the carboxylate, addition of acetic acid resulted in a significantly improved yield of 40 % (entry 2). As was anticipated, the corresponding acetoxycubane **8** and the cross‐Kolbe product **9** were observed as minor by‐products in the crude reaction mixtures. However, the addition of an excess of acetic acid was not found to significantly increase the formation of **8** and **9**.

Doubling the flow rate and maintaining the amount of charge (0.4 mL min^−1^, 400 mA (6.2 F)) led to the isolation of product **7** in 36 % yield in a gram‐scale reaction, along with the formation of the reported by‐products (entry 3). However, attempts to use other alcohols as nucleophiles were hampered by poor solubility of the inorganic base, limiting further development of these conditions. Although heterogeneous solutions can be used in a batch electrochemical setup,^28^ such an approach is not desirable in a flow system. Hence, the use of an organic base was explored, with Et_3_N affording the best results leading to **7** in 44 % yield (entry 4).^33^ Omitting acetic acid led to reduced conversion and only 8 % of the desired product (entry 5).

Carbocation formation is reported to be favoured by using carbon anodes under basic conditions with added perchlorate salts as supporting electrolyte.^27^ In the current work, exchanging the Pt anode for a carbon material (C/PVDF, carbon polyvinylidene fluoride composite) under the previously optimised conditions, resulted in a reduced yield of **7** (12 %, entry 6) alongside decomposition products (^1^H NMR analysis). However, decreasing the applied charge to 2.5 F gave an elevated yield (44 %) of the methyl ether **7** (entry 7), and omission of AcOH achieved a further improvement to 52 % (entry 8). Significantly, anodic oxidation using the C/PVDF anode, 200 mA (2.5 F) and 0.5 mL min^−1^ allowed for the formation of **7** with increased productivity and substantially improved current efficiency (42 % compared to 14 % at Pt).

Decreasing the flow rate, while maintaining the same cell current (double amount of charge, 5.0 F), also gave a decreased yield of 24 % (entry 9), highlighting the potential for overoxidation of the methoxylated product **7**. This was confirmed by resubmitting the reaction mixture containing **7** to the electrolysis conditions, resulting in a decreased yield of 30 % (from 50 % with one pass through the reactor). Different bases and supporting electrolytes were explored (entries 10–13 and the Supporting Information), but the yield was not improved further, and the presence of NaClO_4_ or Et_4_NBF_4_ retarded decarboxylation.

With optimised conditions in hand, we explored different alcohols as nucleophiles (Figure 1). A range of alkoxylated products from alcohols with different steric hindrance were successfully synthesised (**7**, **11**–**13**). This included deuterated ethers, illustrated by the synthesis of methyl 4‐methoxy(*d*_3_)‐1‐cubanecarboxylate (**11**) in 40 % yield. The corresponding crystal structure was obtained (see the Supporting Information, Section 10.4). Selectively deuterated substrates are of interest for medicinal chemistry studies, in which the use of deuteration has shown, in some cases, to improve the pharmacokinetic properties.^34^ Furthermore, fluorinated cubane ethers (**14** and **15**) were obtained in moderate to good yields from TFE (2,2,2‐trifluoroethanol) and HFIP (1,1,1,3,3,3‐hexafluoroisopropanol), respectively. The application of fluorinated alcohols was not viable using the C/PVDF anode, which swells in fluorinated solvents (see the Supporting Information, Section 3.5.3), but was possible using a Pt anode. Other sources of carbon that could be used with fluorinated solvents, such as glassy carbon,^31^, ^35^ showed inferior results for this particular reaction.

**Figure 1:**
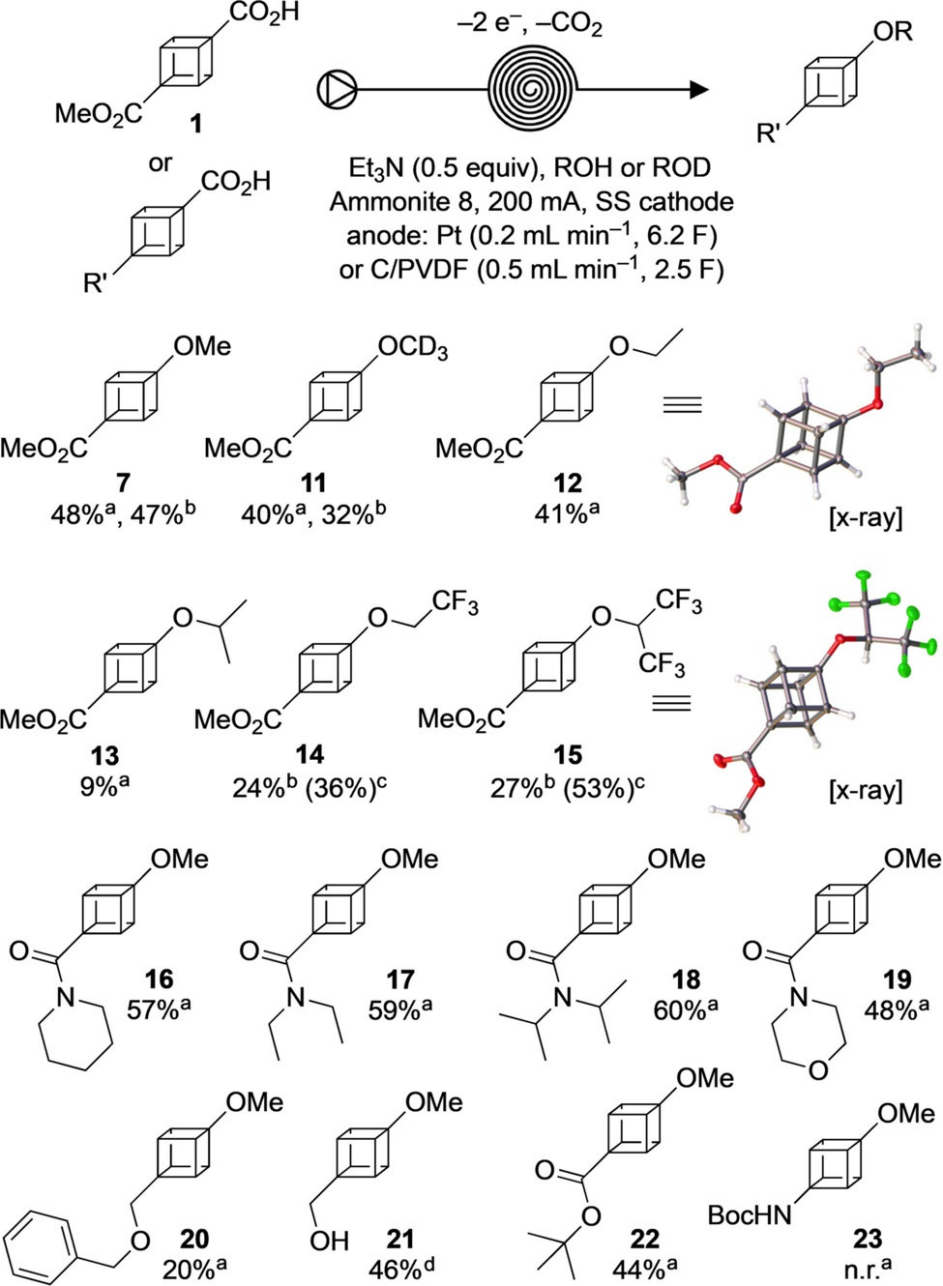
Reaction scope. [a] Isolated yield with C/PVDF, cubanecarboxylic acid [0.1 m]. [b] Isolated yield with Pt anode, 1 equiv AcOH added. [c] ^19^F NMR yield with α,α,α‐trifluorotoluene as internal standard. [d] Isolated yield with C/PVDF, cubanecarboxylic acid [0.05 m].

The substrate scope was further explored by using different functional groups on the cubane moiety, with attention to potential chemoselectivity issues including oxidation of C−H bonds adjacent to nitrogen in amides.^36^ Pleasingly, oxidation of the carboxylate occurred in preference, leading to compounds **16**–**19** in good yields (48–60 %). Piperidine (e.g., **16**) and morpholine rings (e.g., **19**) remained unchanged, offering interesting potential scaffolds for application in drug discovery. Benzyl ethers, which are susceptible to anodic oxidation, were also tolerated albeit with the desired decarboxylative coupling product **20** obtained in a reduced 20 % yield. Gratifyingly, the presence of a free alcohol in the molecule did not disturb the electrochemical transformation and compound **21** was obtained in 46 % yield as the main product. Although a *tert‐*butyl ester is compatible with the reaction conditions (**22**, 44 %), a Boc‐protected amine with or without the use of base did not lead to the desired electrolysis product **23**, and starting material was recovered.

To demonstrate the ease of laboratory scale‐up using the flow electrolysis approach described herein, 12.5 mmol of starting material was successfully oxidised using the same reactor, giving one gram of pure methoxy‐cubane **7** in only four hours (Scheme 2). Although on small scale the formation of methyl 1‐cubanecarboxylate **10** as hydrogenolysis by‐product is insignificant, on gram‐scale of approximately 150 mg of **10** was isolated.

**Scheme 2:**
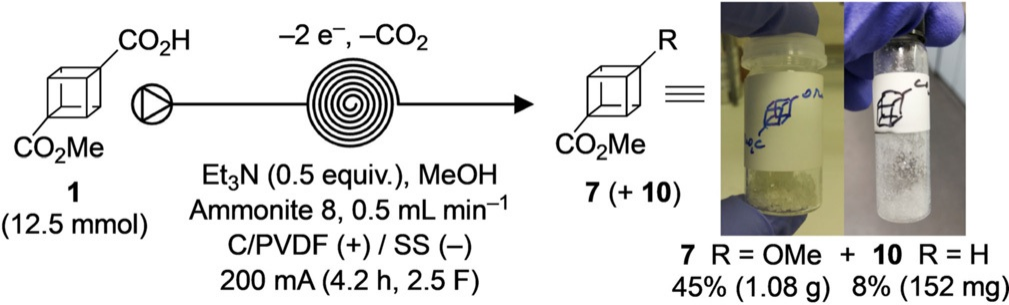
Gram‐scale decarboxylative functionalisation of cubane.

It is worth noting that applying similar conditions in a batch‐type cell (similar geometry to commercial batch reactors), compound **7** was obtained in 50 % yield. However, three hours of electrolysis time was required instead of ten minutes in flow for the same scale (0.5 mmol), and a considerably increased amount of charge (4.0 F) was applied to achieve full conversion (see the Supporting Information, Section 5).

The demonstration of a one‐step Hofer–Moest process in flow making **7** accessible on gram‐scale, opens up possible applications of **7** as primary building block for the synthesis of methoxylated phenyl bioisosteres. Cubanisidine **25** was identified as a bioisostere of the highly electron‐rich and relatively toxic *p*‐anisidine **26** (Scheme 3), and its synthesis was undertaken. Saponification of ester **7** gave 4‐methoxycubane carboxylic acid **24** in 94 % yield, which was followed by Yamada–Curtius^37^ rearrangement to give the corresponding Boc‐amine **23** in 69 % yield. Cleavage of the carbamate protecting group delivered 4‐methoxy‐1‐cubanamine as its hydrochloride salt **25**.

**Scheme 3:**
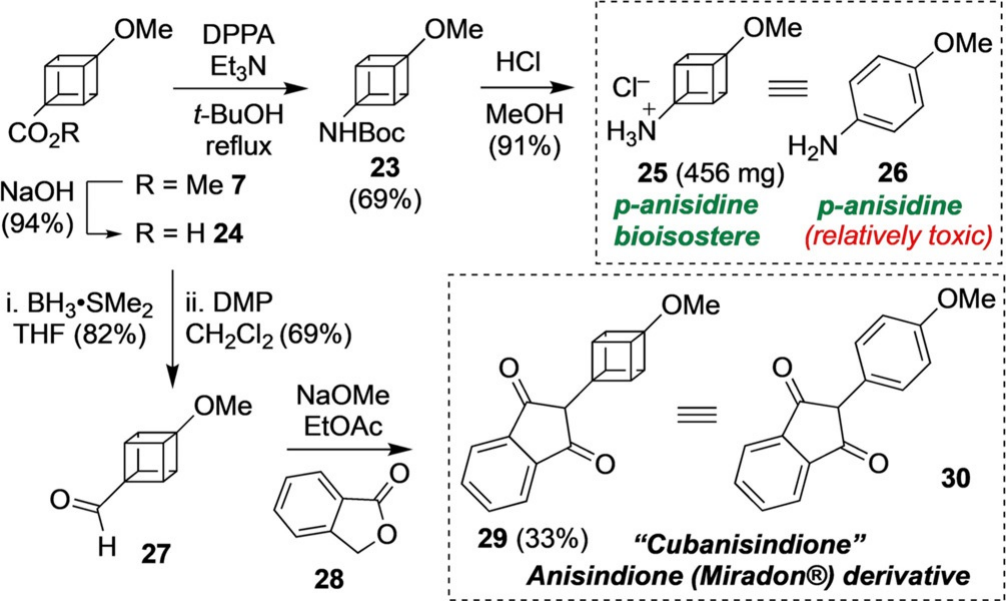
Methyl 4‐methoxy‐1‐cubanecarboxylate (**7**) as a precursor of anisole bioisosteres.

As a further illustrative example, following Williams’ synthesis of a number of pravadoline analogues featuring anisole bioisosteres,21a the cubane derivative of the synthetic drug anisindione (**30**) was investigated. Anisindione (**30**), known under the brand name Miradon^®^, is used as anticoagulant. 4‐Methoxy 1‐cubanecarboxylic acid (**24**) was reduced to 4‐methoxy‐1‐cubanemethanol in 82 % yield, and subsequent oxidation to the corresponding aldehyde **27** was achieved using Dess–Martin periodinane (DMP). Finally, a condensation reaction^38^ of 4‐methoxy‐1‐cubanecarbaldehyde (**27**) and phthalide **28** using NaOMe in EtOAc gave the desired cubanisindione **29** in 33 % yield.

In the electrolysis at a Pt anode (but not a C/PVDF anode), the addition of acetic acid was essential to achieve a satisfactory yield of the methoxylated cubane **7**. Cyclic voltammetry confirmed the electroactive species to be the carboxylate anion (Figure 2); in the absence of Et_3_N, no oxidation peak is observed (green curve) at potentials prior to solvent decomposition, but after the addition of the base a clear peak (red curve) is seen. This peak is unchanged by the addition of acetic acid (blue line, see the Supporting Information for further details, Section 6). The poor yield of **7** at Pt in the absence of acetic acid is surprising: We postulate that the presence of acetic acid modifies the properties of the electrode surface, possibly by adsorbed methyl radicals.^39^ In addition, the observation of the cross Kolbe‐product **9** and the hydrogenolysis product (**10**) indicate that the cubyl radical is an intermediate in the formation of the carbocation.

**Figure 2:**
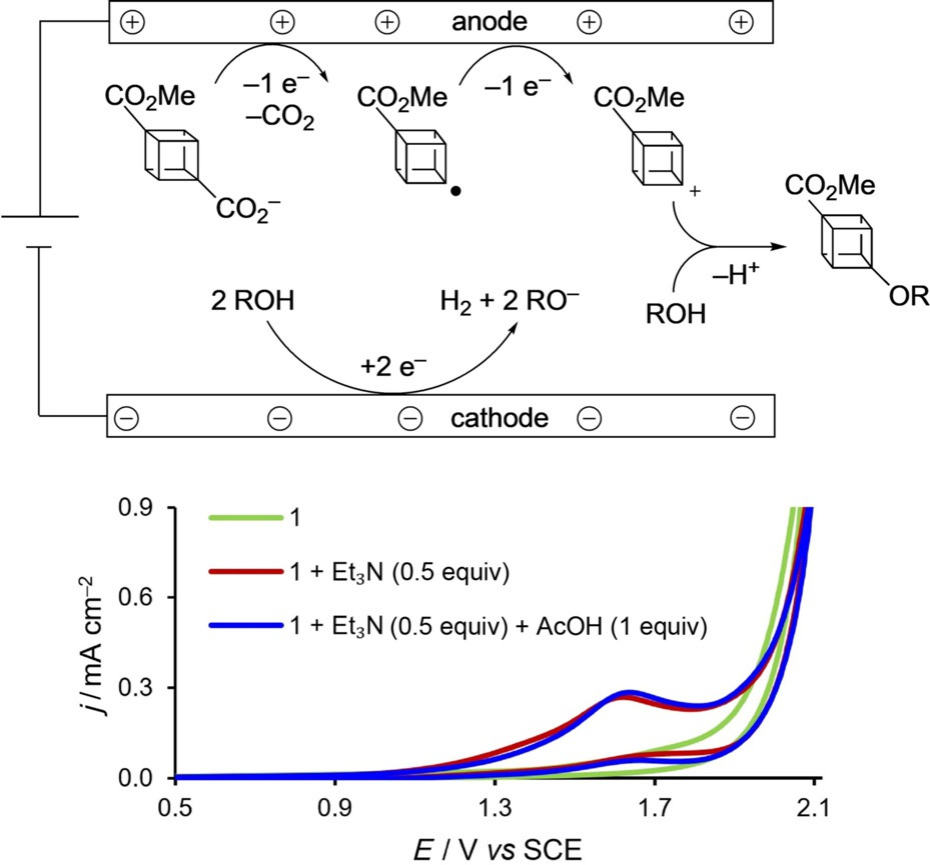
Proposed mechanism and cyclic voltammograms of the acidic Hofer–Moest reaction conditions.

In conclusion, to the best of our knowledge, we report the first electrochemical cubane ring system functionalisation, through a Hofer–Moest oxidative decarboxylative ether formation. Successful investigation of the substrate scope involving potentially electrochemically oxidisable functionalities clearly indicates the mildness of the reaction conditions. The use of flow electrolysis facilitates laboratory scale‐up, as was shown by a gram‐scale synthesis in a matter of hours in the same reactor and under the same conditions, nicely demonstrating the advantage of this approach. A further straightforward three‐step process provided a cubanisidine biosiostere building block, and another example of a methoxycubane analogue of a drug is described. Organic electrosynthesis is considered as a sustainable methodology, because electrical current replaces potentially hazardous/toxic and costly chemical reagents and any ensuing waste stream.^40^ This is clearly shown herein, with facile access to the methoxycubane core from the commercially available **1** by only using methanol as solvent and triethylamine (0.5 equiv) as reagent. No supporting electrolytes were required, facilitating purification. Hence, this first demonstration of an electrochemical reaction involving cubanes will be of great interest in medicinal and materials chemistry, with great promise for further developments. Further applications are under investigation in our laboratories.

## Conflict of interest

The authors declare no conflict of interest.

## Supporting information

As a service to our authors and readers, this journal provides supporting information supplied by the authors. Such materials are peer reviewed and may be re‐organized for online delivery, but are not copy‐edited or typeset. Technical support issues arising from supporting information (other than missing files) should be addressed to the authors.

Supplementary
