# Supplementary material for: Cubane Electrochemistry: Direct Conversion of Cubane Carboxylic Acids to Alkoxy Cubanes Using the Hofer–Moest Reaction under Flow Conditions
Source: Chemistry. 2019 Nov 7;26(2):374–8. doi: 10.1002/chem.201904479 (PMC6973092; doi:10.1002/chem.201904479)
Supplement: Supplementary file 1 — Supplementary [file CHEM-26-374-s001.pdf]

CHEMISTRY
A European Journal

Supporting Information

Cubane Electrochemistry: Direct Conversion of Cubane Carboxylic Acids to Alkoxy Cubanes Using the Hofer–Moest Reaction under Flow Conditions

Diego E. Collin, Ana A. Folgueiras-Amador, Derek Pletcher, Mark E. Light, Bruno Linclau,* and Richard C. D. Brown*[a]

chem_201904479_sm_miscellaneous_information.pdf

Contents

1 General Experimental 8
2 Experimental setup for the Hofer–Moest reaction (Figures S1–S2) 9
3 Optimisation of the reaction parameters for cubane decarboxylation 10
3.1 GC analysis 10
3.1.1 Experimental procedure for GC yield calculations: 10
3.2 Byproducts obtained using Platinum anode in presence of acetic acid 15
3.3 Solubility screen of bases (Table S3) 17
3.4 Screening conditions using Platinum anode 18
3.5 Screening conditions with Carbon/PVDF anode 21
3.5.1 Base screening 21
3.5.2 Electrolyte screening 21
3.5.3 Electrode stability in fluorinated solvents (Figure S12) 22
4 Flow Setup 23
4.1 Calculation for the current needed in the flow cell (I_{theo}) and charge applied (F) 23
4.2 Platinum electrode 23
4.3 C/PVDF electrode 23
5 Batch Setup 24
5.1 Calculation of the current needed in the batch cell (I_{theo}) and charge applied (F) 24
5.1.1 C/PVDF electrode 24
6 Cyclic Voltammetry 26
7 Synthetic procedures 30
7.1 General procedure A: Electrolysis with platinum electrode 30
7.2 General procedure B: Electrolysis with Carbon/PVDF electrode 30
7.3 Gramme-scale synthesis of methyl 4-methoxy-1-cubanecarboxylate (7) 30
7.4 Methyl 4-methoxy-1-cubanecarboxylate (7) 32
7.5 Methyl cubanecarboxylate (10) 32
7.6 Synthesis of methyl 4-methoxy(d_3)-1-cubanecarboxylate (11) 33
7.7 Methyl 4-ethoxy-1-cubanecarboxylate (12) 33
7.8 Methyl 4-isopropoxy-1-cubanecarboxylate (13) 34
7.9 Methyl 4-trifluoroethoxy-1-cubanecarboxylate (14) 34
7.10 Methyl 4-(1,1,1,3,3,3-hexafluoroisopropoxy)-1-cubanecarboxylate (15) 35
7.11 Synthesis of (4-methoxycubyl)(piperidin-1-yl)methanone (16) 36
7.12 Synthesis of *N*,*N*-diethyl-4-methoxycubane-1-carboxamide (17) 36

7.13 Synthesis of *N*,*N*-diisopropyl-4-methoxycubane-1-carboxamide (18) 37
7.14 Synthesis of (4-methoxycubyl)(morpholino)methanone (19) 37
7.15 Synthesis of 4-methoxy-1-cubanebenzylether (20) 38
7.16 Synthesis of 4-methoxy-1-cubylmethanol (21) 39
7.17 Synthesis of *tert-butyl* 4-methoxy-1-cubanecarboxylate (22) 39
7.18 Synthesis of 4-methoxy-1-cubanecarboxylic acid (24) 40
7.19 Synthesis of *tert*-butyl 4-methoxy-1-cubylcarbamate (23) 40
7.20 Synthesis of 4-methoxy-1-cubanammine hydrochloride (25) 41
7.21 Synthesis of 4-methoxy-1-cubancarbaldehyde (27) 42
7.22 Synthesis of Cubanisindione (29) 42
7.23 Synthesis of 4-methoxy-1-cubylmethanol (21) 43
7.24 Synthesis of *tert*-butyl 4-methyl cubanedicarboxylate (S1) 43
7.25 Synthesis of methyl 4-hydroxymethyl-1-cubanecarboxylate (S2) 44
7.26 Synthesis of methyl 4-benzyloxymethyl-1-cubanecarboxylate (S3) 45
7.27 Synthesis of methyl 4-((*tert*-butoxycarbonyl)amino)cubane-1-carboxylate (S4) 45
7.28 General procedure C for the synthesis of amides 47
7.29 Synthesis of methyl 4-(piperidine-1-carbonyl)cubane-1-carboxylate (S5) 47
7.30 Synthesis of methyl 4-(diethylcarbamoyl)cubane-1-carboxylate (S6) 48
7.31 Synthesis of methyl 4-(diisopropylcarbamoyl)cubane-1-carboxylate (S7) 48
7.32 Synthesis of methyl 4-(morpholine-4-carbonyl)cubane-1-carboxylate (S8) 49
8 General procedure D for the synthesis of cubanecarboxylic acids 50
8.1 Synthesis of 4-*tert*-butoxycarbonyl-1-cubanecarboxylic acid (S9) 50
8.2 Synthesis of 4-hydroxymethyl-1-cubanecarboxylic acid (S10) 51
8.3 Synthesis of 4-benzyloxymethyl-1-cubanecarboxylic acid (S11) 51
8.4 Synthesis of 4-((*tert*-butoxycarbonyl)amino)cubane-1-carboxylic acid (S12) 52
8.5 Synthesis of 4-(piperidine-1-carbonyl)cubane-1-carboxylic acid (S13) 52
8.6 Synthesis of 4-(diethylcarbamoyl)cubane-1-carboxylic acid (S14) 53
8.7 Synthesis of 4-(diisopropylcarbamoyl)cubane-1-carboxylic acid (S15) 53
8.8 Synthesis of 4-(morpholine-4-carbonyl)cubane-1-carboxylic acid (S16) 54
9 NMR Spectra 55
9.1 Methyl 4-methoxy-1-cubanecarboxylate (7) 55
9.1.1 ^{1}H NMR (400 MHz, $CDCl_3$) 55
9.1.2 ^{13}C NMR (101 MHz, $CDCl_3$) 55
9.2 Methyl cubanecarboxylate (10) 56

9.2.1 ^{1}H NMR (400 MHz, $CDCl_3$) 56
9.2.2 ^{13}C NMR (101 MHz, $CDCl_3$) 56
9.3 Methyl 4-methoxy(d_3)-1-cubanecarboxylate (11) 57
9.3.1 ^{1}H NMR (400 MHz, $CDCl_3$) 57
9.3.2 ^{13}C NMR (101 MHz, $CDCl_3$) 57
9.4 Methyl 4-ethoxy-1-cubanecarboxylate (12) 58
9.4.1 ^{1}H NMR (400 MHz, $CDCl_3$) 58
9.4.2 ^{13}C NMR (101 MHz, $CDCl_3$) 58
9.5 Methyl 4-isopropoxy-1-cubanecarboxylate (13) 59
9.5.1 ^{1}H NMR (400 MHz, $CDCl_3$) 59
9.5.2 ^{13}C NMR (101 MHz, $CDCl_3$) 59
9.6 Methyl 4-trifluoroethoxy-1-cubanecarboxylate (14) 60
9.6.1 ^{1}H NMR (400 MHz, $CDCl_3$) 60
9.6.2 ^{13}C NMR (101 MHz, $CDCl_3$) 60
9.6.3 ^{19}F NMR (376 MHz, $CDCl_3$) 61
9.7 Methyl 4-(1,1,1,3,3,3-hexafluoroisopropoxy)-1-cubanecarboxylate (15) 62
9.7.1 ^{1}H NMR (400 MHz, $CDCl_3$) 62
9.7.2 ^{13}C NMR (126 MHz, $CDCl_3$) 62
9.7.3 ^{19}F NMR (376 MHz, CDl_3) 63
9.7.4 (4-Methoxycubyl)(piperidin-1-yl)methanone (16) 64
9.7.5 ^{1}H NMR (400 MHz, $CDCl_3$) 64
9.7.6 ^{13}C NMR (101 MHz, $CDCl_3$) 64
9.8 *N*,*N*-Diethyl-4-methoxycubane-1-carboxamide (17) 65
9.8.1 ^{1}H NMR (400 MHz, $CDCl_3$) 65
9.8.2 ^{13}C NMR (101 MHz, $CDCl_3$) 65
9.8.3 *N*,*N*-Diisopropyl-4-methoxycubane-1-carboxamide (18) 66
9.8.4 ^{1}H NMR (400 MHz, $CDCl_3$) 66
9.8.5 ^{13}C NMR (101 MHz, $CDCl_3$) 66
9.9 (4-Methoxycubyl)(morpholino)methanone (19) 67
9.9.1 ^{1}H NMR (400 MHz, $CDCl_3$) 67
9.9.2 ^{13}C NMR (101 MHz, $CDCl_3$) 67
9.10 4-Methoxy-1-cubanebenzylether (20) 68
9.10.1 ^{1}H NMR (400 MHz, $CDCl_3$) 68
9.10.2 ^{13}C NMR (101 MHz, $CDCl_3$) 68

9.11 4-Methoxy-1-cubylmethanol (21) 69
9.11.1 ^{1}H NMR (400 MHz, $CDCl_3$) 69
9.11.2 ^{13}C NMR (101 MHz, $CDCl_3$) 69
9.12 *tert*-Butyl 4-methoxy-1-cubanecarboxylate (22) 70
9.12.1 ^{1}H NMR (400 MHz, $CDCl_3$) 70
9.12.2 ^{13}C NMR (101 MHz, $CDCl_3$) 70
9.13 *tert*-Butyl 4-methoxy-1-cubylcarbamate (23) (from 24) 71
9.13.1 ^{1}H NMR (400 MHz, $CDCl_3$) 71
9.13.2 ^{13}C NMR (126 MHz, $CDCl_3$) 71
9.14 4-Methoxy-1-cubanecarboxylic acid (24) 72
9.14.1 ^{1}H NMR (400 MHz, $CDCl_3$) 72
9.14.2 ^{13}C NMR (101 MHz, $CDCl_3$) 72
9.15 4-Methoxy-1-cubanamine hydrochloride (25) 73
9.15.1 ^{1}H NMR (400 MHz, D_2O) 73
9.15.2 ^{13}C NMR (101 MHz, D_2O) 73
9.16 4-Methoxy-1-cubancarbaldehyde (27) 74
9.16.1 ^{1}H NMR (400 MHz, $CDCl_3$) 74
9.16.2 ^{13}C NMR (101 MHz, $CDCl_3$) 74
9.17 Cubanisindione (29) 75
9.17.1 ^{1}H NMR (400 MHz, $CDCl_3$) 75
9.17.2 ^{13}C NMR (400 MHz, $CDCl_3$) 75
9.18 *tert*-Butyl 4-methyl cubanedicarboxylate (S1) 76
9.18.1 ^{1}H NMR (400 MHz, $CDCl_3$) 76
9.18.2 ^{13}C NMR (101 MHz, $CDCl_3$) 76
9.19 Methyl 4-hydroxymethyl-1-cubanecarboxylate (S2) 77
9.20 ^{1}H NMR (400 MHz, $CDCl_3$) 77
9.20.1 ^{13}C NMR (101 MHz, $CDCl_3$) 77
9.21 Methyl 4-benzyloxymethyl-1-cubanecarboxylate (S3) 78
9.21.1 ^{1}H NMR (400 MHz, $CDCl_3$) 78
9.21.2 ^{13}C NMR (101 MHz, $CDCl_3$) 78
9.22 Methyl 4-((*tert*-butoxycarbonyl)amino)cubane-1-carboxylate (S4) 79
9.22.1 ^{1}H NMR (500 MHz, $CDCl_3$) 79
9.22.2 ^{13}C NMR (126 MHz, $CDCl_3$) 79
9.23 Methyl 4-(piperidine-1-carbonyl)cubane-1-carboxylate (S5) 80

9.23.1 ^{1}H NMR (400 MHz, $CDCl_3$) 80
9.23.2 ^{13}C NMR (101 MHz, $CDCl_3$) 80
9.24 Methyl 4-(diiethylcarbamoyl)cubane-1-carboxylate (S6) 81
9.24.1 ^{1}H NMR (400 MHz, $CDCl_3$) 81
9.24.2 ^{13}C NMR (101 MHz, $CDCl_3$) 81
9.25 Methyl 4-(diisopropylcarbamoyl) cubane-1-carboxylate (S7) 82
9.25.1 ^{1}H NMR (400 MHz, $CDCl_3$) 82
9.25.2 ^{13}C NMR (101 MHz, $CDCl_3$) 82
9.26 Methyl 4-(morpholine-4-carbonyl)cubane-1-carboxylate (S8) 83
9.26.1 ^{1}H NMR (400 MHz, $CDCl_3$) 83
9.26.2 ^{13}C NMR (101 MHz, $CDCl_3$) 83
9.27 4-*tert*-Butoxycarbonyl-1-cubanecarboxylic acid (S9) 84
9.27.1 ^{1}H NMR (400 MHz, $CDCl_3$) 84
9.27.2 ^{13}C NMR (101 MHz, $CDCl_3$) 84
9.28 4-Hydroxymethyl-1-cubanecarboxylic acid (S10) 85
9.28.1 ^{1}H NMR (400 MHz, CD_3OD) 85
9.28.2 ^{13}C NMR (101 MHz, CD_3OD) 85
9.29 Methyl 4-benzyloxy-1-cubanecarboxylic acid (S11) 86
9.29.1 ^{1}H NMR (400 MHz, $CDCl_3$) 86
9.29.2 ^{13}C NMR (101 MHz, $CDCl_3$) 86
9.30 4-((*tert*-Butoxycarbonyl)amino)cubane-1-carboxylic acid (S12) 87
9.30.1 ^{1}H NMR (500 MHz, CD_3OD) 87
9.30.2 ^{13}C NMR (126 MHz, CD_3OD) 87
9.31 4-(Piperidine-1-carbonyl)cubane-1-carboxylic acid (S13) 88
9.31.1 ^{1}H NMR (400 MHz, $CDCl_3$) 88
9.31.2 ^{13}C NMR (101 MHz, $CDCl_3$) 88
9.32 4-(Diethylcarbamoyl)cubane-1-carboxylic acid (S14) 89
9.32.1 ^{1}H NMR (400 MHz, $CDCl_3$) 89
9.32.2 ^{13}C NMR (101 MHz, $CDCl_3$) 89
9.33 4-(Diisopropylcarbamoyl)cubane-1-carboxylic acid (S15) 90
9.33.1 ^{1}H NMR (400 MHz, $CDCl_3$) 90
9.33.2 ^{13}C NMR (101 MHz, $CDCl_3$) 90
9.34 4-(Morpholine-4-carbonyl)cubane-1-carboxylic acid (S16) 91
9.34.1 ^{1}H NMR (400 MHz, $CDCl_3$) 91

9.34.2 ^{13}C NMR (101 MHz, $CDCl_3$) 91

10 X-Ray Crystallographic Data 92

10.1 Methyl 4-methoxy-1-cubanecarboxylate [CCDC: 1905443 2019sot0002_K1_100K] 7 92

10.2 Methyl 4-(1,1,1,3,3,3-hexafluoroisopropoxy)-1-cubanecarboxylate [CCDC: 1889723 2018sot0040_K1_100K] 15 94

10.3 Methyl 4-ethoxy-1-cubanecarboxylate [CCDC: 1919701 2019sot0009_K1_100K] 12 96

10.4 Methyl 4-methoxy(d_3) cubanecarboxylate (11) [CCDC: 1919978 2019sot0008_R1_100K] 98

11 References 100

1 General Experimental

Methanol (Fisher Scientific, HPLC grade) and all other solvents and reagents were used as received from chemical suppliers unless otherwise stated. Tetraethylammonium tetrafluoroborate (Alfa Aesar, 99%) was recrystallised from hot methanol and dried at 60 ºC under reduced pressure (~10 mbar) for 24 h.

TLC was performed on aluminium-precoated plates coated with silica gel 60 with an F254 indicator; visualised under UV light (254 nm) and/or by staining with cerium ammonium molybdate (CAM). Flash column chromatography was performed with Sigma Aldrich 60 silica gel (40–63 micron).

Fourier-transform infrared (FT-IR) spectra are reported in wavenumbers (cm^{-1}) and were recorded using a diamond ATR accessory, as solids or neat liquids.

^{1}H NMR, ^{13}C NMR and ^{19}F NMR spectra were recorded in $CDCl_3$, D_2O or CD_3OD solutions at 298 K at 400 MHz, 101 MHz and 376 MHz, or 500 MHz and 126 MHz, respectively. Chemical shifts are reported in δ units using $CHCl_3$ (δ 7.27 ppm ^{1}H, δ 77.00 ppm ^{13}C), H_2O (δ 4.75 ppm ^{1}H) and CH_3OH (δ 3.31, δ 4.87 ppm ^{1}H, 49.15 ppm ^{13}C) as an internal standards. Coupling constants (*J*) were recorded in Hz and are corrected. The following abbreviations for the multiplicity of the peaks are s (singlet), d (doublet), t (triplet), q (quartet), quint (quintet), sxt (sextet), spt (septet), oct (octet) br (broad), and m (multiplet). Melting points were obtained in an open capillary and are uncorrected. Electrospray resolution mass spectra were recorded on a H2Os ZMD quadrupole spectrometer. High resolution mass spectra were recorded on a Bruker Daltonics MaXis mass spectrometer equipped with a time of flight analyzer.

Yields of compound **7** and remaining starting material **1** were determined by gas chromatography using a Shimadzu GC 2014 equipped with an autosampler, FID detector and Agilent technologies HP5 column (length 30 m, I.D. 0.32 mm, film thickness 0.25 µm). The results were processed using GC Solution Lite software. Separations were carried out using He as carrier gas with a flow rate of 2.48 mL min^{-1} through the column. A split injection was conducted using a split ratio of 100:1. The injection and detector temperatures were maintained at 280 and 295 °C, respectively. The oven temperature was initially held at 60 ºC and then programmed to increase at 10 ºC min^{-1} to 250 °C, where it was held for 2 min. The GC was calibrated using a range of solutions of known concentration of both the starting material and the product.

Syntheses were carried out in an Ammonite 8 flow cell (Cambridge Reactor Design) with platinum or C/PVDF (carbon/ polyvinylidene fluoride composite) anodes and 316L stainless steel cathode. Full details of the Ammonite 8 have been published elsewhere,[1] and the reactor is available commercially from Cambridge Reactor Design Ltd. (www.cambridgereactordesign.com). The cell current was controlled with a Rapid Electronics switching mode power supply (85-1903). A peristaltic pump (Ismatec® REGLO Digital Ms-2/6) was used to flow the solutions through the electrochemical cell.

2 Experimental setup for the Hofer–Moest reaction (Figures S1–S2)

Syntheses were carried out in an Ammonite 8 flow cell (Cambridge Reactor Design) with platinum or carbon anode and 316L stainless steel cathode.[1] This cell has a spiral electrolyte channel, 1 m in length and 2 mm in width and the interelectrode gap is 0.5 mm (Figure S1).

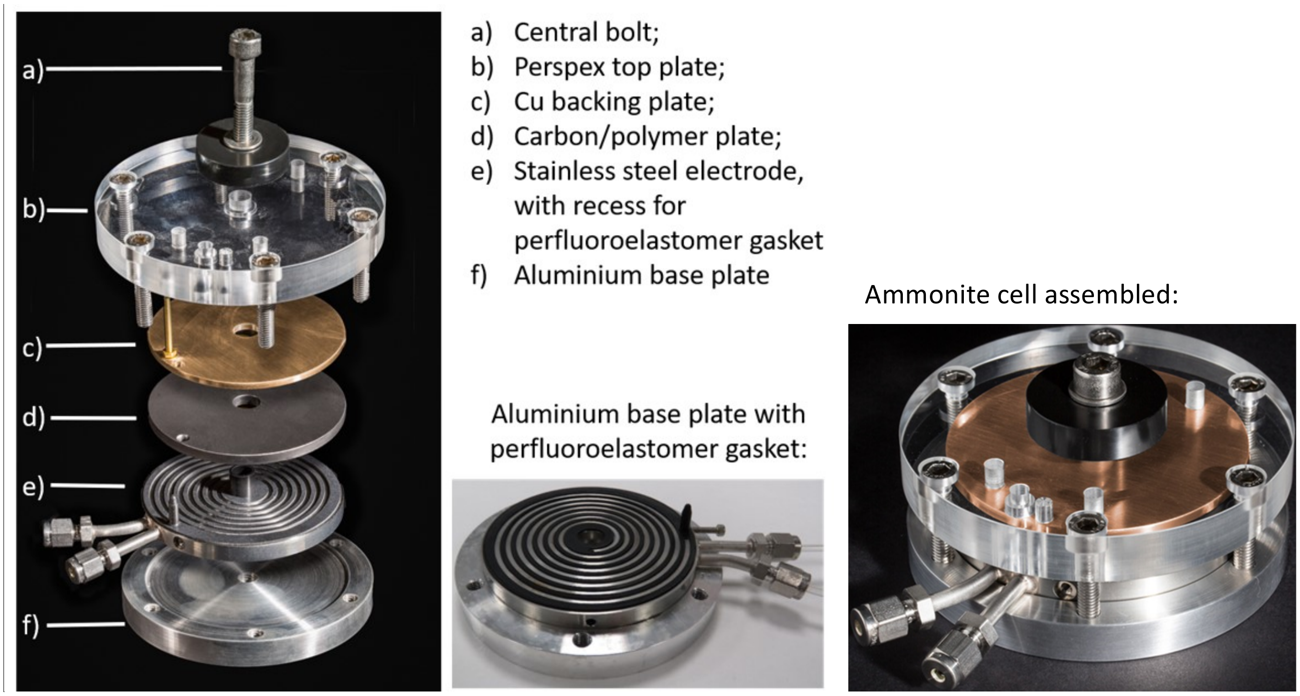

Figure S1: Pictures of the Ammonite 8 electrolysis cell.

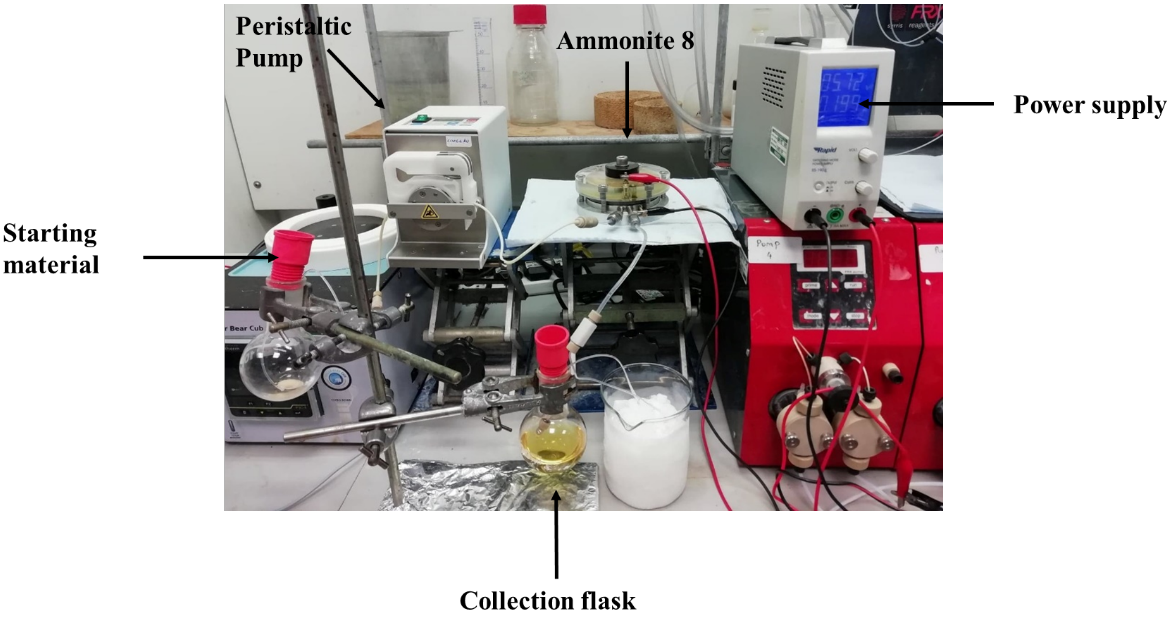

Figure S2: Picture of the experimental setup. From left to right: REGLO Digital Ms-2/6 peristaltic pump, Ammonite 8 flow cell and Rapid Electronics switching mode power supply.

3 Optimisation of the reaction parameters for cubane decarboxylation

For the analysis of the crude mixtures, quantification by ^{1}H NMR was not possible as many of the protons of the formed cubane-type products overlap with each other. Therefore, the reaction conditions were studied by using calibrated GC with the starting material **1** and methyl 4-methoxy-1-cubanecarboxylate (**7**). Due to inadequate separation of methyl 4-methyl-1-cubanecarboxylate (**9**) and methyl 1-cubanecarboxylate (**10**), and the small amount of byproducts collected as a mixture, the GC was only calibrated using methyl 4-methoxy-1-cubanecarboxylate (**7**). All optimisation reactions analysed by GC were carried out on 0.25 mmol scale.

3.1 GC analysis

3.1.1 Experimental procedure for GC yield calculations:

4-Methoxycarbonyl-1-cubanecarboxylic acid (**1**, 51 mg, 0.25 mmol), Et_3N (17 µL, 0.125 mmol, 0.5 equiv.) and, if necessary depending on the general procedure used, AcOH (15 µL, 0.25 mmol, 1 equiv.) were added to methanol to make up 2.5 mL total volume. This sample was pumped into the reactor using the specified conditions (flow rate and current), and collected in a 5 mL volumetric flask (dilution to C = 0.05 M). Once all the solution had passed through the reactor, pure methanol was passed through the reactor and collected in the same 5 mL volumetric flask until the total sample volume was 5 mL. Samples were analysed by calibrated GC to determine the corresponding yields.

NOTE: GC yields reported are for the entire sample rather than steady state. Moreover, due to gas formation during the reaction (cathode: H_2 and anode: CO_2), the flow rate was not constant during electrolyses, and therefore, residence time changes during the course of the experiment.

Calibration curves were created for 4-methoxycarbonyl cubanecarboxylic acid (**1**) and methyl 4-methoxy cubanecarboxylate (**7**).

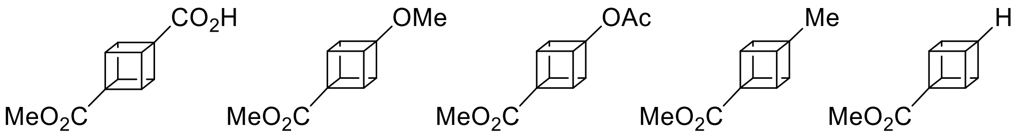

1 **7** **8** **9** **10**

Table S1: Retention times of compounds analysed by GC.

Compound	Retention time [min]
4-Methoxycarbonyl cubanecarboxylic acid **1**	13. 36
Methyl 4-methoxy-1-cubanecarboxylate **7**	10.11
Methyl 4-methyl-1-cubanecarboxylate **9**	8.00
Methyl 4-acetoxy-1-cubanecarboxylate **8**	11.54
Methyl cubanecarboxylate **10**	8.26

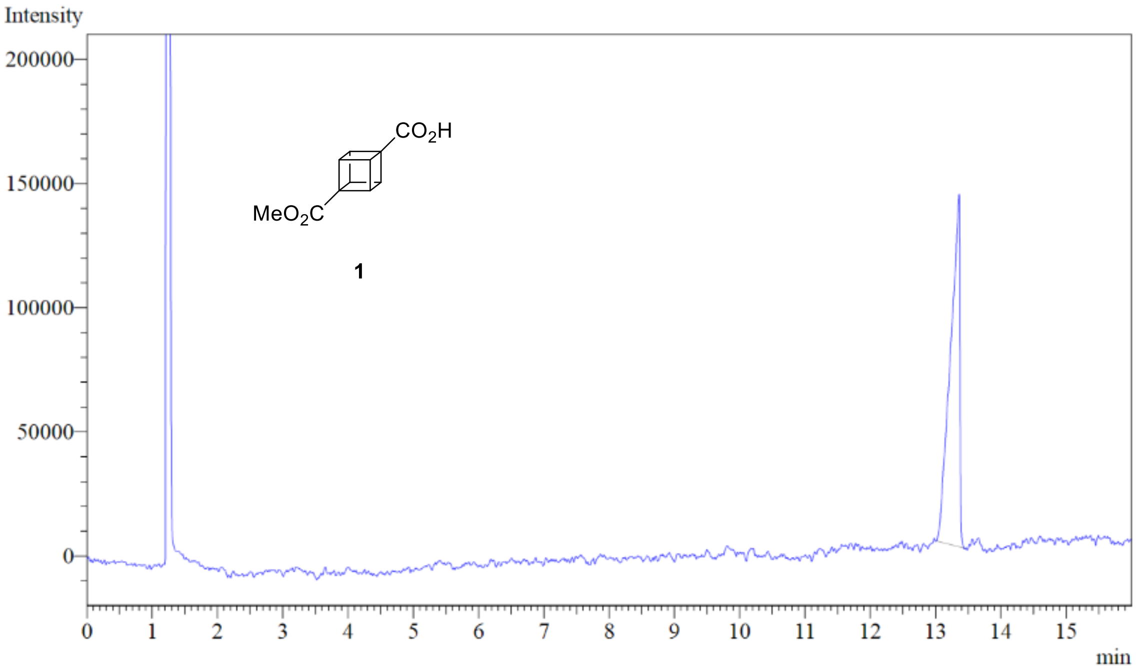

Figure S3: GC chromatogram of 4-methoxycarbonyl-1-cubanecarboxylic acid (**1**), retention time: 13.3 min.

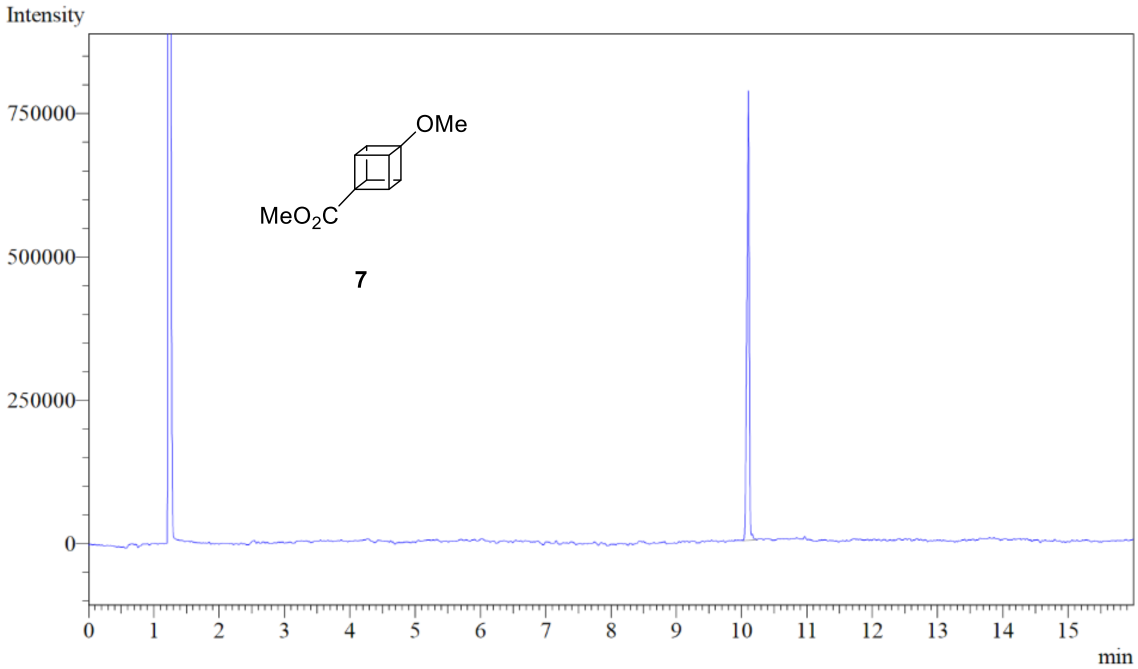

Figure S4: GC chromatogram of pure methyl 4-methoxy-1-cubanecarboxylate (**7**), retention time: 10.1 min.

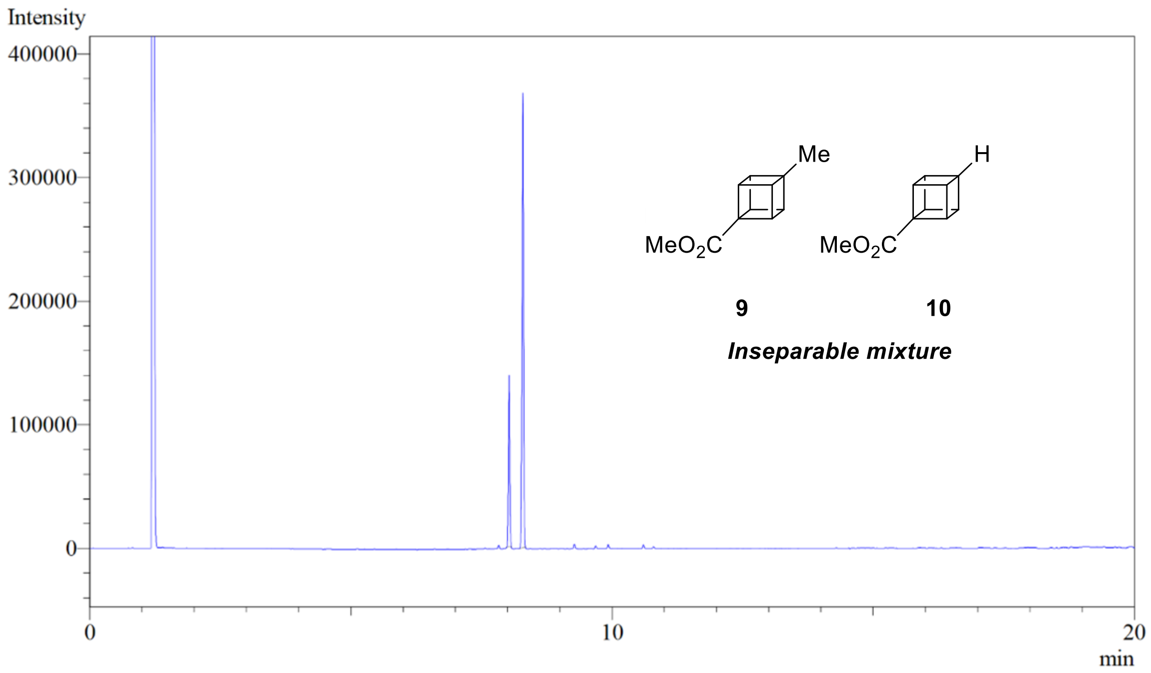

Figure S5: GC chromatogram of isolated mixture of compounds **9** (methyl 4-methyl-1-cubanecarboxylate, retention time: 8.0 min) and **10** (methyl cubanecarboxylate, retention time: 8.29 min). We were not able to separate this mixture by column chromatography.

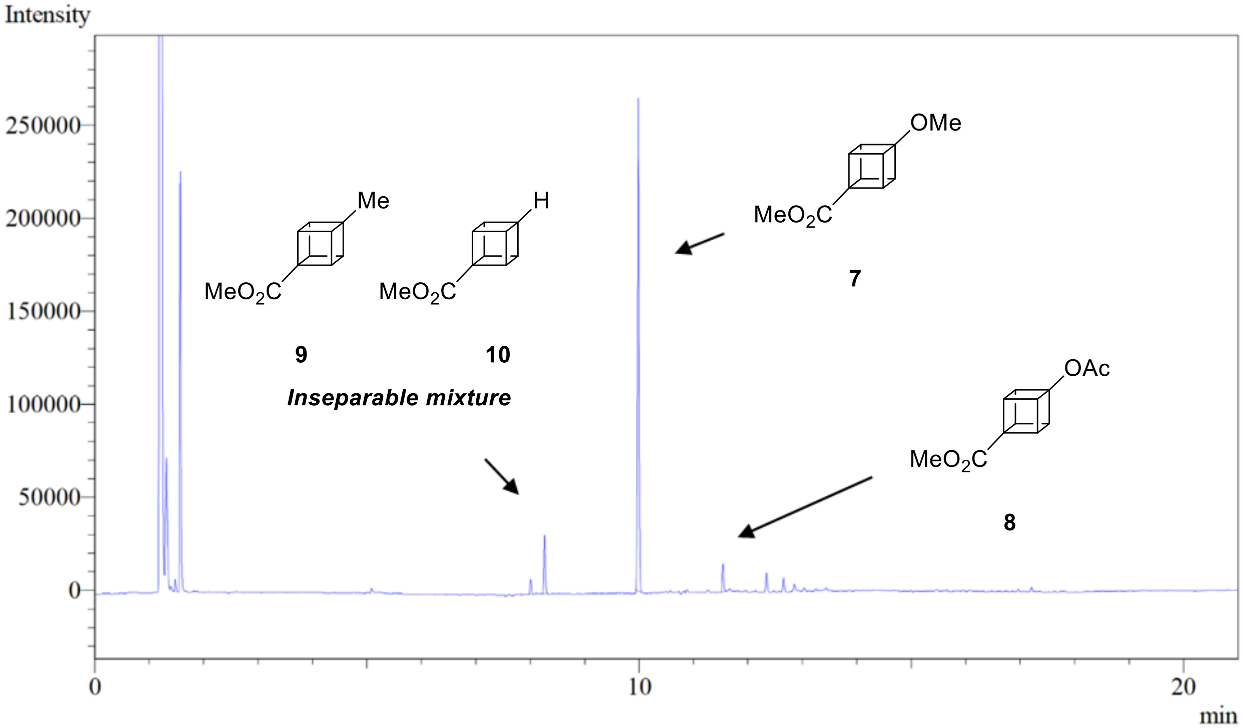

Peak#	Ret.Time	Area	Height	Conc.	Unit	Mark	ID#	Cmpd Name
1	8.007	14690	7931	0.000				
2	8.263	57697	31441	0.000				
3	9.987	497191	260369	0.022	M		2	Cubane product MeO
4	11.542	27853	14716	0.000				
Total		597431	314457					

Figure S6: GC chromatogram of crude mixture collected at the outlet of the reactor under the optimised conditions with Pt electrode.

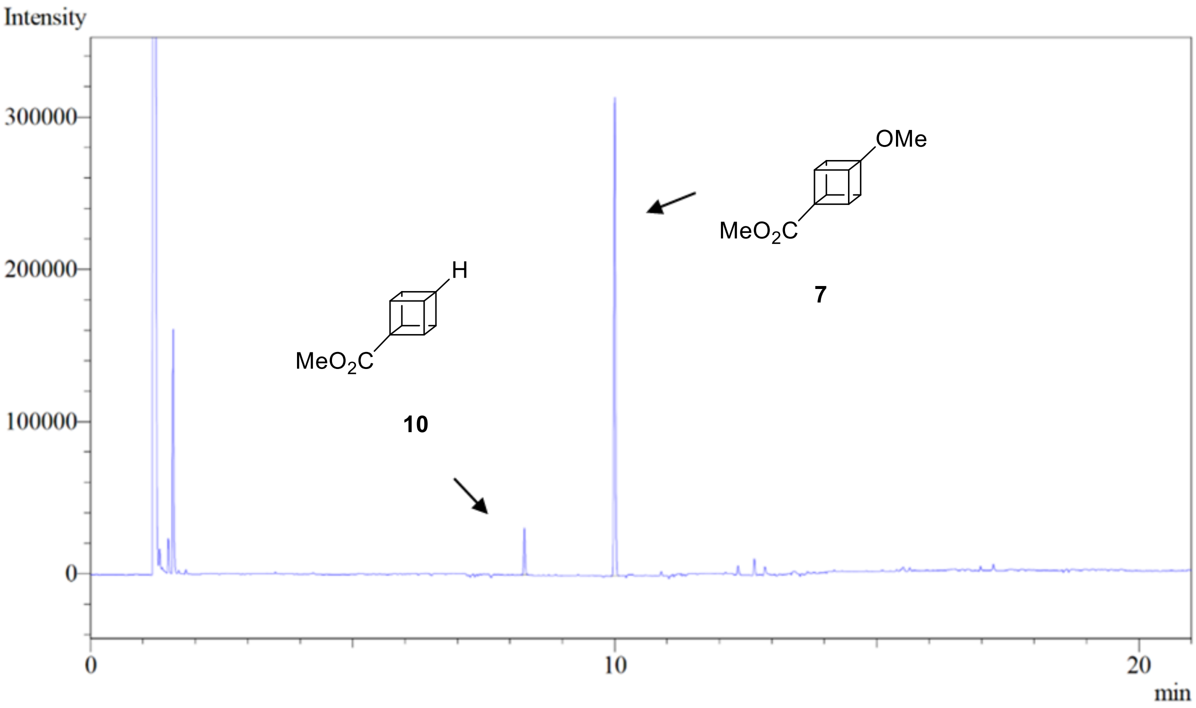

Peak#	Ret.Time	Area	Height	Conc.	Unit	Mark	ID#	Cmpd Name
1	8.277	55835	30437	0.000				
2	10.002	600959	305653	0.026	M		2	Cubane product MeO
Total		656794	336090					

Figure S7: GC chromatogram of crude mixture collected at the outlet of the reactor under the optimized conditions with C/PVDF electrode.

3.1.1.1 GC Area Ratio

The following table shows the area ratio of byproducts compared to the main compound **7** under the optimised conditions using Pt and C/PVDF electrodes (see GC chromatogram above). The ratios were similar for other conditions screened.

Table S2: GC area ratio for main product **7** and byproducts.

Conditions	GC Area Ratio
Pt	100:3:12:6 (**7:8:9:10**)
C/PVDF	100:9 (**7:10**)

3.2 Byproducts obtained using Platinum anode in presence of acetic acid

Quantification by ^{1}H NMR was not possible as many of the protons of the formed cubane-containing products overlap with each other. Moreover, methyl 4-methyl-1-cubanecarboxylate (**9**) and methyl cubanecarboxylate (**10**) were not separable by column chromatography.

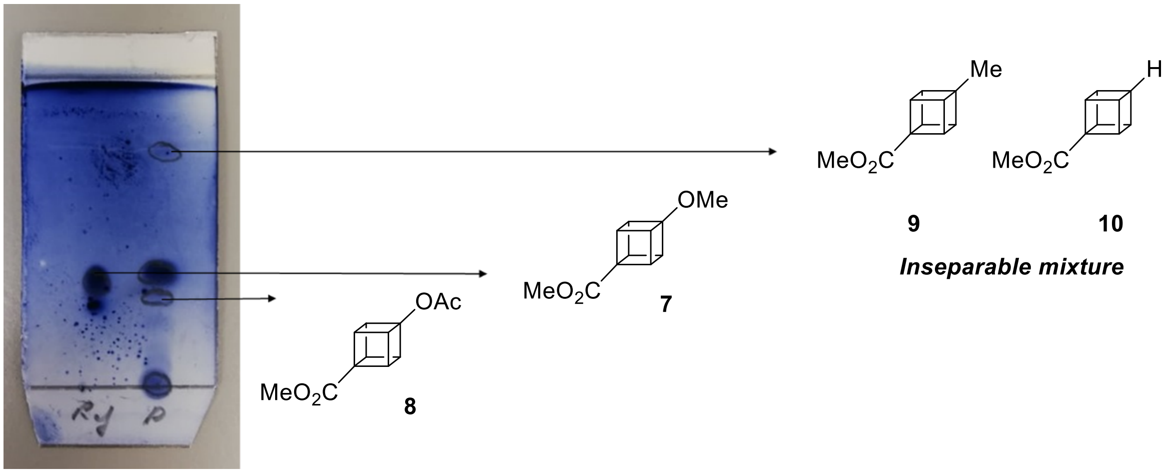

Figure S8: TLC (pentane/Et_2O, 9:1)of crude mixture collected at the outlet of the reactor under the optimised conditions. (Stain: CAM).

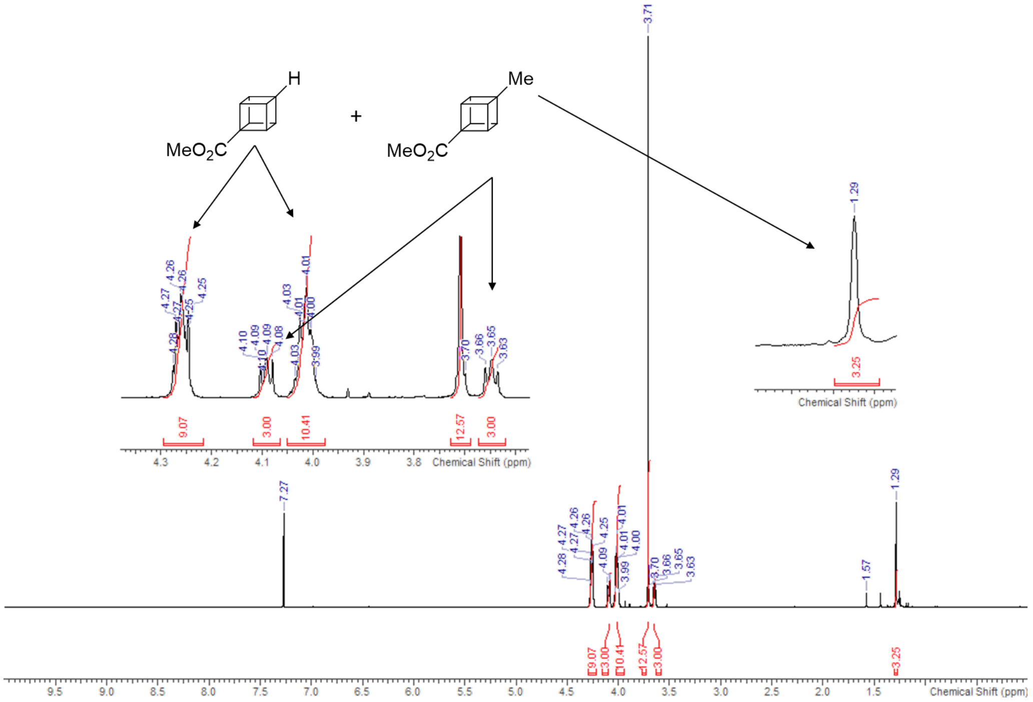

Figure S9: ^{1}H NMR of the mixture of methyl cubanecarboxylate (**10**) and methyl 4-methyl-1-cubanecarboxylate (**9**).

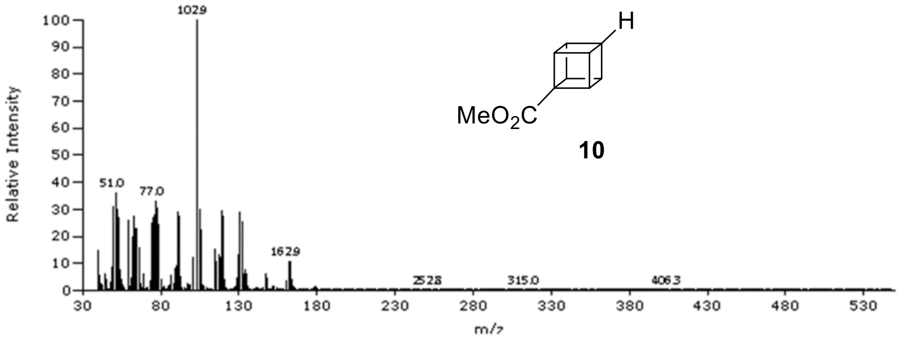

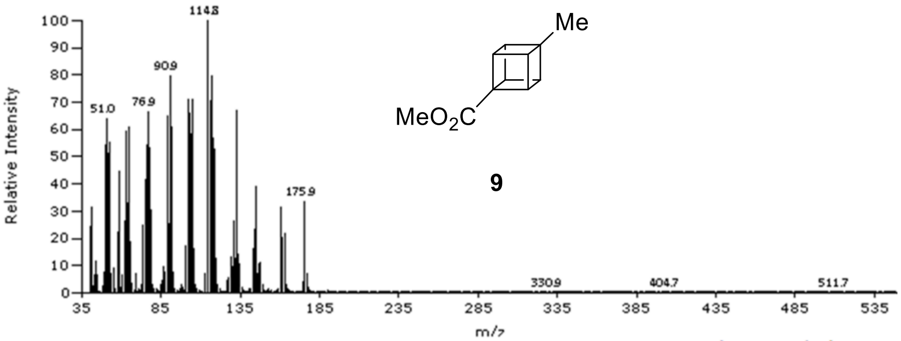

Figure S10: LRMS of methyl cubanecarboxylate (**10**, MW: 162.19 g mol^{-1}) and methyl 4-methyl-1-cubanecarboxylate (**9**, MW: 176.21 g mol^{-1}).

3.3 Solubility screen of bases (Table S3)

A qualitative solubility study has been carried out in order to find homogenous conditions for the reaction.

General conditions: Concentration of starting material **1**, c = 0.1 M, 0.5 equivalent of base.

Table S3: Qualitative analysis of the solubility of **1** in different solvents.

**Solvent** / **Base**	**KOH**	**Et_3N**
methanol	Soluble	Soluble
ethanol	Soluble	Soluble
isopropanol	Insoluble	Soluble
n-propanol	Insoluble	Soluble
CF_3CH_2OH	Soluble	Soluble
HFIP	Soluble	Soluble

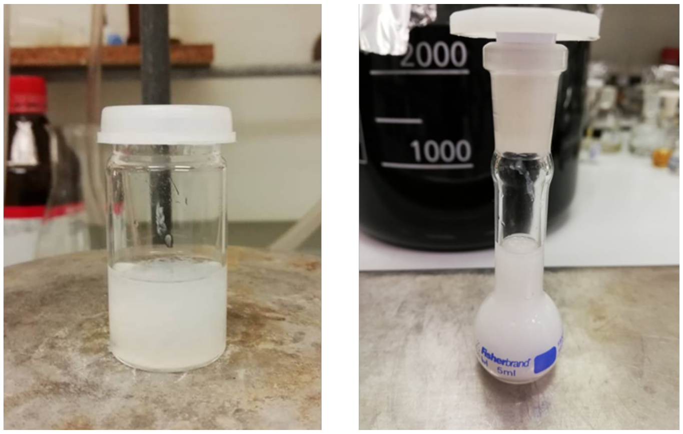

Figure S11: (Left**)** Precipitation in isopropanol with KOH (0.5 equiv.) as base. (Right) Precipitation in isopropanol with K_2CO_3 (5 mol %) as base. In both cases, a very viscous gel formed.

3.4 Screening conditions using Platinum anode

Table S4: Base screen.

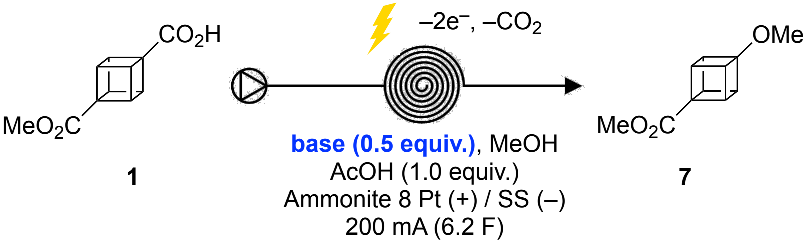

Entry	Base	Flow rate [mL min^{-1}]	GC yield[a] [%]
1	Et_3N	0.2	44
2[b]	Et_3N	0.5	6
3	2,6-lutidine	0.2	46[c]
4	DBU	0.2	44
5	KOH	0.2	46
6[b]	KOH[d]	0.2	14

[a] Calculated using calibrated GC; [b] no AcOH added; [c] Required current was not achieved under the applied limited potential (12 V); [d] 1.0 equiv. of KOH.

When DBU and KOH were used, a large amount of solid deposit was observed on the working electrode. Potassium hydroxide is not soluble in other alcohols, which makes this base unsuitable for a wider scope of substrates.

Table S5: Further conditions screen (Pt anode).

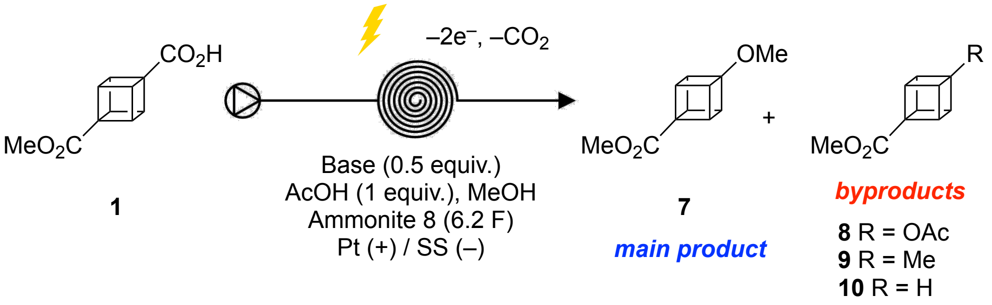

Entry	Base	Flow rate [mL min^{-1}]	Current [mA] (charge)	RSM [%][a]	Yield of 7 [%][a]
1[b]	KOH	0.2	200	None	7
2	KOH	0.2	200	None	50
3	Et_3N	0.1	100	None	12
4[c]	KOH	0.4	400	–	36[d]
5	Et_3N	0.2	200	None	44
6	Et_3N	0.1	100	69	10
7	Et_3N	0.4	400	10	32
8[b]	Et_3N	0.2	200	None	40
9[e]	Et_3N	0.2	200 (12.4 F)	None	33
10[f]	Et_3N	0.2	200	60	20

General conditions: 0.25 mmol of **1**, C = 0.1 M, Pt anode. [a] RSM (Remaining Starting Material) and yield of **1** were calculated using a calibrated GC. [b] 5 equiv. AcOH. [c] Scale: 1.03 g of **1** gave 350 mg of **7**. [d] Isolated yield. [e] C = 0.05 M. [f] no AcOH.

Table S6: Additive and/or supporting electrolyte screening (Pt anode).

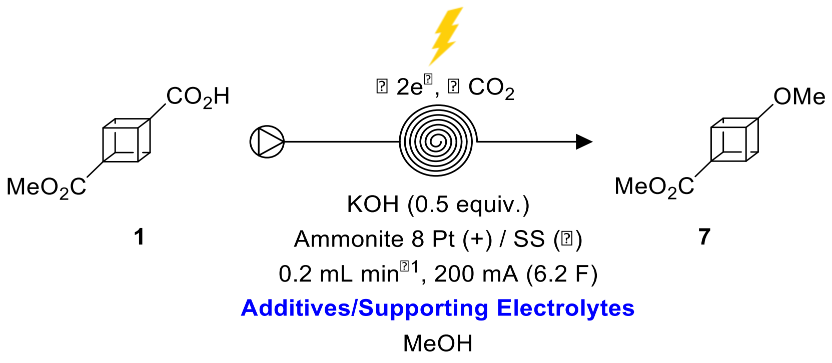

Entry	Additives/Supporting Electrolytes	Yield [%]
1	None	14[a]
2	AcOH (1 equiv.)	36[b]
3	TFA (5 equiv.)	traces
4	$NaClO_4$ (0.5 equiv.)	traces
5	pivalic acid (1 equiv.)	22[b]
6	Et_4NBF_4 (0.5 equiv.)	traces
7	$LiClO_4$ (0.5 equiv.)	traces

[a]GC Yield. [b]isolated yield.

3.5 Screening conditions with Carbon/PVDF anode

3.5.1 Base screening

Table S7: Base screening.

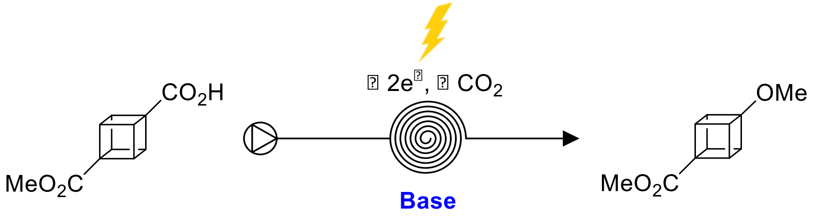

1 Ammonite 8 C/PVDF (+) / SS (−) 7

0.5 mL min^{-1}, 200 mA (2.5 F)

MeOH

Entry	Base	Equiv. of base	GC Yield [a] [%]
1		0.10	32
2	Et_3N	0.25	28
3		0.50	52
4		0.75	36[b]
5	2,6-lutidine	0.50	52[c]
6	DBU	0.50	52
7	K_2CO_3	0.05	34[d]

[a] Calculated by calibrated GC; [b] 22% of **1** remaining. [c] Deposit on the electrode; [d] 160 mA current (2.0 F).

3.5.2 Electrolyte screening

Table S8: Supporting electrolyte screening (C/PVDF anode).

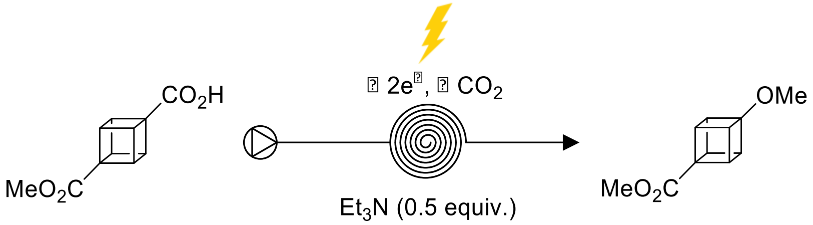

Et_3N (0.5 equiv.)

1 Ammonite 8 C/PVDF (+) / SS (−) 7

0.5 mL min^{-1}, 200 mA (2.5 F)

Additives/ Supporting Electrolytes

MeOH

Entry	Additives/ Supporting Electrolytes [5 mM]	Remaining SM [%]	GC Yield [a] [%]
1	$NaClO_4$	32	4
2	Et_4NBF_4	20	12
3	-	0	52

[a] Calculated by calibrated GC.

3.5.3 Electrode stability in fluorinated solvents (Figure S12)

The C/PVDF electrode was found to be incompatible with fluorinated solvents, as shown in Figure S12. Erosion of graphite has been previously reported in the literature when using fluorinated solvents such as HFIP or TFE.[2]

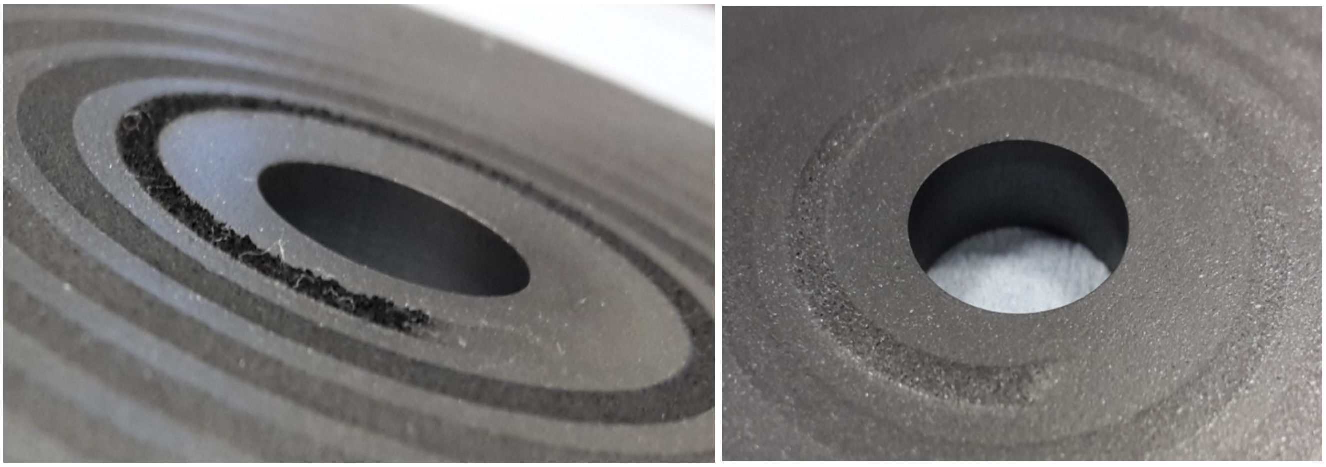

Figure S12: (Left**)** C/PVDF after a reaction run in HFIP. (Right) C/PVDF after being polished shows how it remains swollen where it was in contact with HFIP in the flow channel.

4 Flow Setup

4.1 Calculation for the current needed in the flow cell (I_{theo}) and charge applied (F)

The *theoretical current* (I_{theo}) needed for in a flow electrochemical process can be calculated using the following equation:

$$\text{Faraday's law applied to flow conditions: } I_{theo} = n\, F\, C\, Q_v$$

- I_{theo} = Theoretical current (A)
- n = number of electrons involved in the electrochemical process
- F = Faraday's constant (96 485 s A mol^{-1})
- C = Concentration (mol mL^{-1})
- Q_v = Flow rate (mL s^{-1})

The *current efficiency (CE)* of the electrochemical process can be calculated using the following equation:

$$\text{Current efficiency: CE} = \frac{I_{theo}}{I_{cell}} \times \text{yield\%}$$

4.2 Platinum electrode

The Hofer–Moest reaction is to a 2-electron oxidation process (n = 2). The optimised conditions using a Pt electrode were identified as:

Concentration = 0.1 mol L^{-1} = 0.0001 mol mL^{-1}

Flow rate = 0.2 mL min^{-1} = 0.00333 mL s^{-1}

Therefore, with the equation shown above, the theoretical current (I_{theo}) for this transformation can be calculated:

I_{theo} = 0.064 A

But the applied cell current (I_{cell}) is 0.2 A, which means that a 3.1 fold excess of current is used. This is the stoichiometry of current required, and is represented by "*a*":

$$I_{cell} = x\, F\, C\, Q_v \qquad \text{Where:} \qquad x = n\, a$$

$$x = \frac{0.2}{0.0001 \times 0.00333 \times 96485} = 6.2$$

"6.2 F (in Coulombs) is the charge applied to the electrochemical reaction with a Pt electrode"

Yield of **2** = 44%

$$\text{CE} = \frac{0.064}{0.2} \times 44\% = \mathbf{14\%}$$

4.3 C/PVDF electrode

Similarly, for the C/PVDF electrode:

Concentration = 0.1 mol L^{-1} = 0.0001 mol mL^{-1}

Flow rate = 0.5 mL min^{-1} = 0.00833 mL s^{-1}

I_{theo} = 0.16 A

I_{cell} = 0.2 A

Yield of **2** = 52%

x = 2.5 → *"2.5 F (Coulombs) is the charge applied to the electrochemical reaction with a C/PVDF electrode"*

$$\text{CE} = \mathbf{42\%}$$

5 Batch Setup

5.1 Calculation of the current needed in the batch cell (I_{theo}) and charge applied (F)

5.1.1 C/PVDF electrode

The *theoretical electrolysis time (t_{theo})* needed in a batch electrochemical process can be calculated using the following equation:

$$\text{Faraday's law applied to batch conditions: } t_{theo} = \frac{n\,mF}{I}$$

- t_{theo} = time of electrolysis (s)
- I = Current applied (A)
- n = number of electrons involved in the electrochemical process
- m = moles of substrate to be electrolysed (mol)
- F = Faraday's constant (96 485 s A mol^{-1})

The *current efficiency (CE)* of the electrochemical process for a batch-type reaction can be calculated using:

$$\text{Current efficiency: CE} = \frac{t_{theo}}{t_{real}} \times \text{yield\%}$$

The Hofer–Moest reaction is to a 2-electron oxidation process (n = 2).

- *Surface area:* The surface area of the batch electrode used was 1.8 cm^2.
- *Current density:* The cell averaged current density used was the same as for the reaction in the Ammonite 8: 10 mA/cm^2. Therefore:
- *Current applied (I):* 10 mA/cm^2 x 1.8 cm^2 = 18 mA.
- *Moles of substrate (n):* 0.5 mmol (in 5 mL)

Therefore, with the equation shown above, the electrolysis time (t_{theo}) for this transformation can be calculated:

t_{theo} = 5400 s (90 min)

But the reaction required 180 min (10800 s) to achieve >95% conversion of starting material, which means that a 2 fold excess of current was used. This is the stoichiometry of current needed, and is represented by "*a*":

$$t = \frac{x\,m\,F}{I} \qquad \text{Where:} \quad x = n\,a$$

$$x = \frac{0.018 \times 10800}{0.0005 \times 96485} = 4.0$$

"4.0 F (in Coulombs) is the charge applied to the batch electrochemical reaction with a C/PVDF electrode"

GC Yield of **7** = 50%

$$CE = \frac{5400}{10800} \times 50\% = \mathbf{25\%}$$

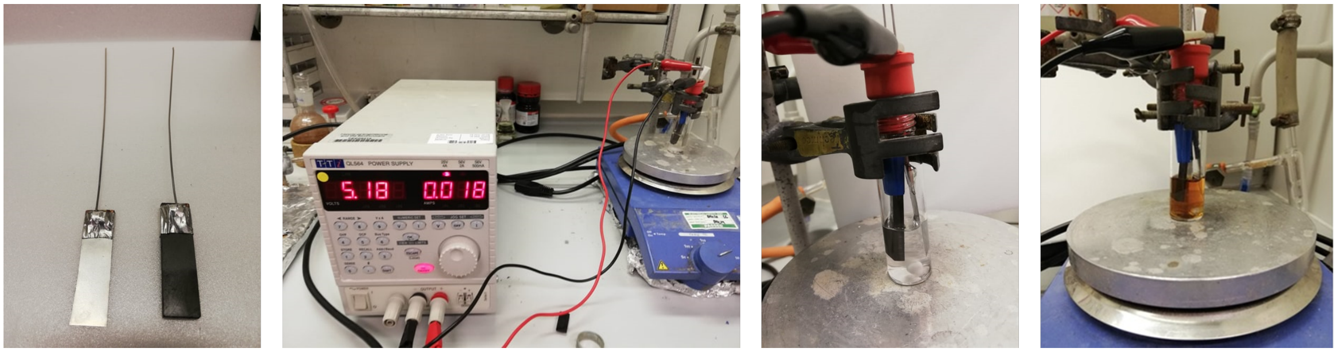

Figure S13: (*From left to right):* Steel and carbon/PVDF electrodes; Power supply (18 mA); Solution before electrolysis; Solution after 3 h of electrolysis.

Experimental procedure (batch electrolysis):

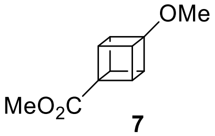

7

The acid **1** (103 mg, 0.500 mmol) and Et_3N (35 µL, 0.25 mmol) were dissolved in MeOH (5 mL) in a vial (see Figure S13). A C/PVDF anode and a stainless steel cathode (12 mm wide) were submerged in the solution, having a working surface of 1.8 cm^2. The solution was stirred and a constant current of 18 mA was applied. An aliquot was analysed by GC after 2 F of charge was passed (90 min), and the amount of product **7** was estimated to be 25% (50% starting material **1** remaining). The electrolysis was continued for another 90 minutes (4.0 F in total), and analysed by GC. The amount of compound **7** was estimated to be 50% (GC yield) with full consumption of starting material. This is comparable to the GC yield obtained using the flow reactor conditions.

6 Cyclic Voltammetry

Cyclic voltammetry was carried out in a three electrode, two-compartment cell with a vitreous carbon disc (diameter 3 mm) working electrode, a Pt wire counter electrode and an aqueous SCE reference electrode mounted in a Luggin capillary. An Autolab PGStat204 potentiostat with Nova 1.9 software was used and responses were analysed using Nova 1.9 software.

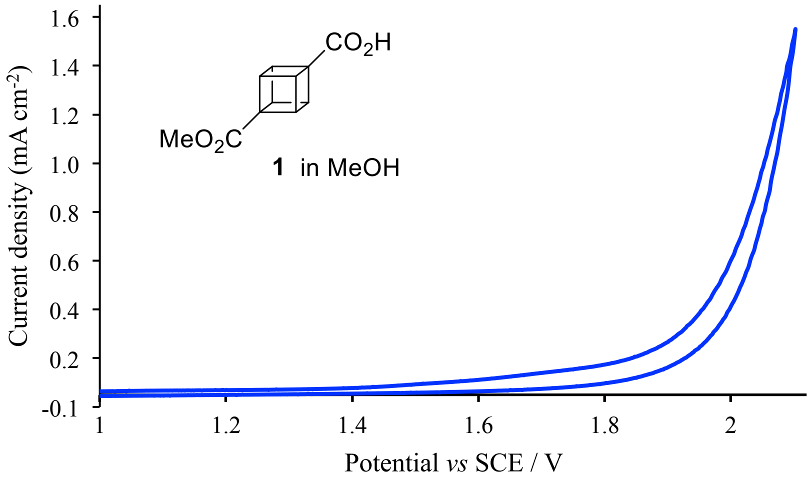

Figure S14: Cyclic voltammogram for 5 mM cubane carboxylic acid (**1**) in MeOH/0.1 M Et_4NBF_4. Potential scan rate 25 mV s^{-1}.

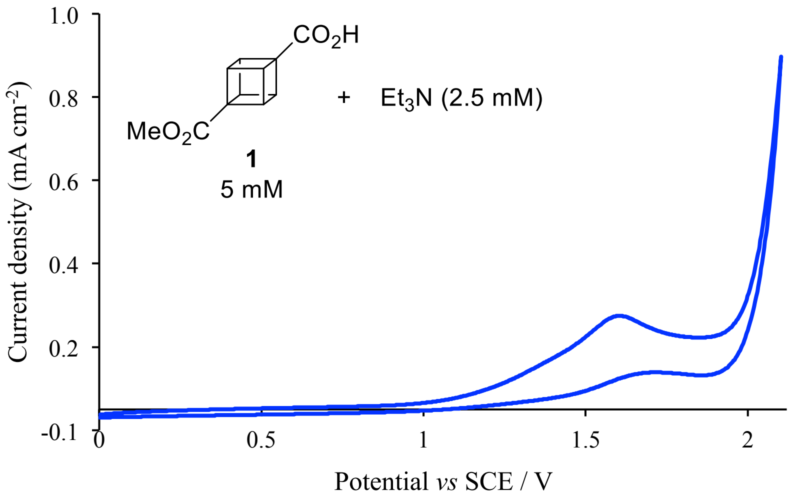

Figure S15: Cyclic voltammogram for 5 mM cubane carboxylic acid (**1**) and 2.5 mM Et_3N in MeOH/0.1 M Et_4NBF_4. Potential scan rate 25 mV s^{-1}

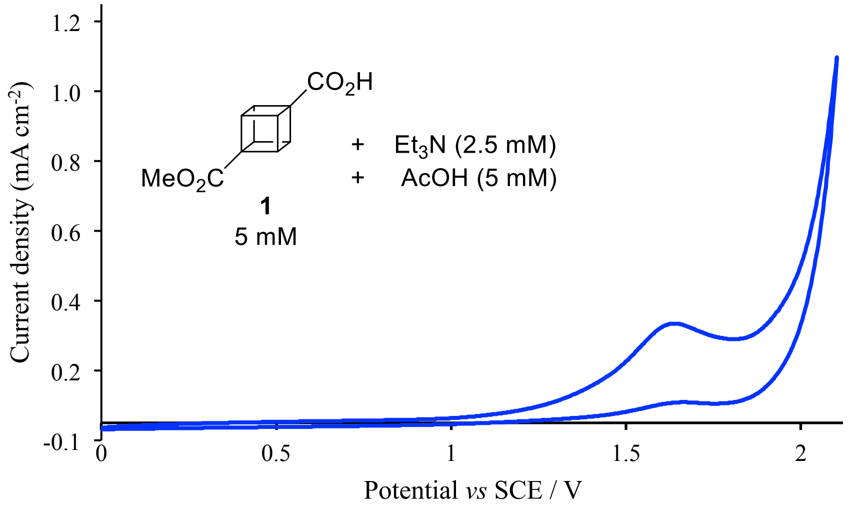

Figure S16: Cyclic voltammogram for 5 mM cubane carboxylic acid (**1**), 2.5 mM Et_3N and 5 mM AcOH in MeOH/0.1 M Et_4NBF_4. Potential scan rate 25 mV s^{-1}.

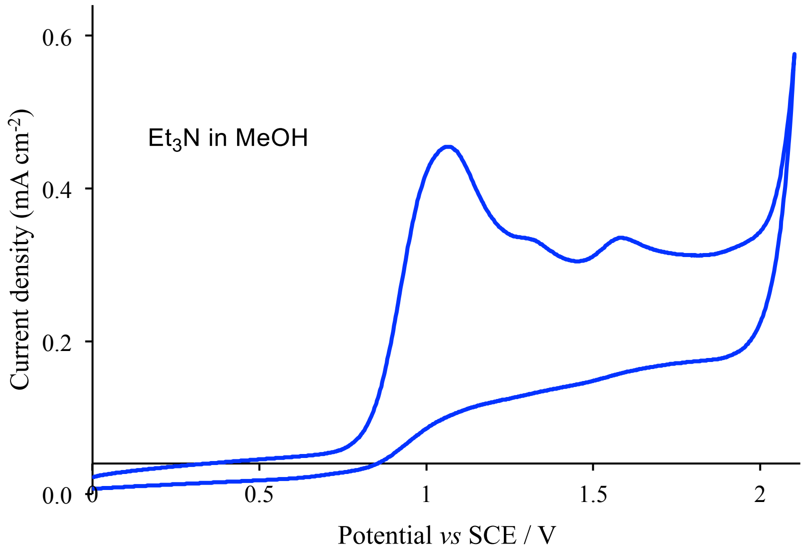

Figure S17: Cyclic voltammogram for 2.5 mM Et_3N in MeOH/0.1 M Et_4NBF_4. Potential scan rate 100 mV s^{-1}.

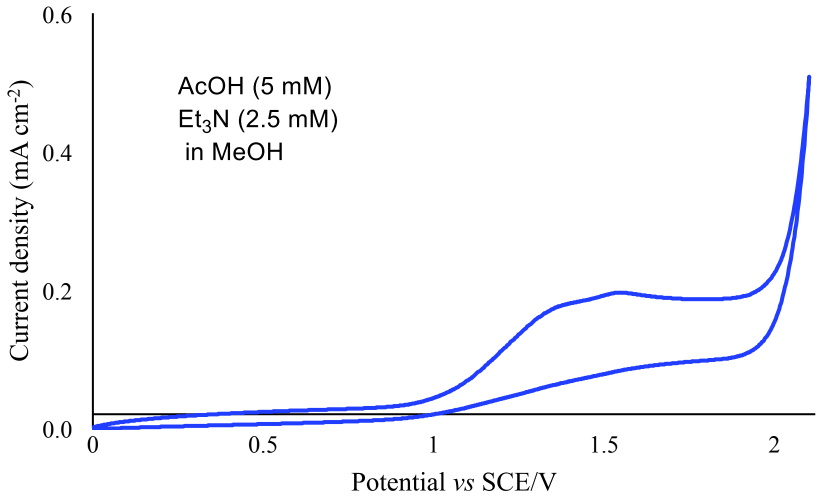

Figure S18: Cyclic voltammogram for 5 mM AcOH + 2.5 mM Et_3N in MeOH/0.1 M Et_4NBF_4. Potential scan rate 25 mV s^{-1}.

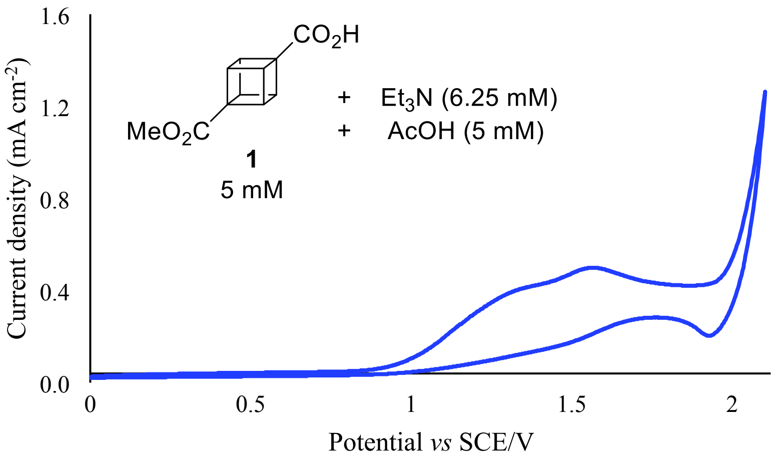

Figure S19: Cyclic voltammogram for 5 mM cubane **1** + 5 mM AcOH + 6.25 mM Et_3N in MeOH/0.1 M Et_4NBF_4. Potential scan rate 25 mV s^{-1}.

If an oxidation or reduction is diffusion controlled, the peak heights between experiments should change by a factor equal to the square root of the change in scan rate. *e.g.* Going from 25 mV/s to 100 mV/s is a change by a factor of 4. Therefore, peak heights in these 2 experiments should differ by a factor of 2 (i.e. double or half) if the process occurring is diffusion controlled rather than being dependant on chemical rate or being mass transfer limited. According to the results shown in Figure S20, we can confirm that this process is diffusion controlled and does not depend on the rate of subsequent chemical steps.

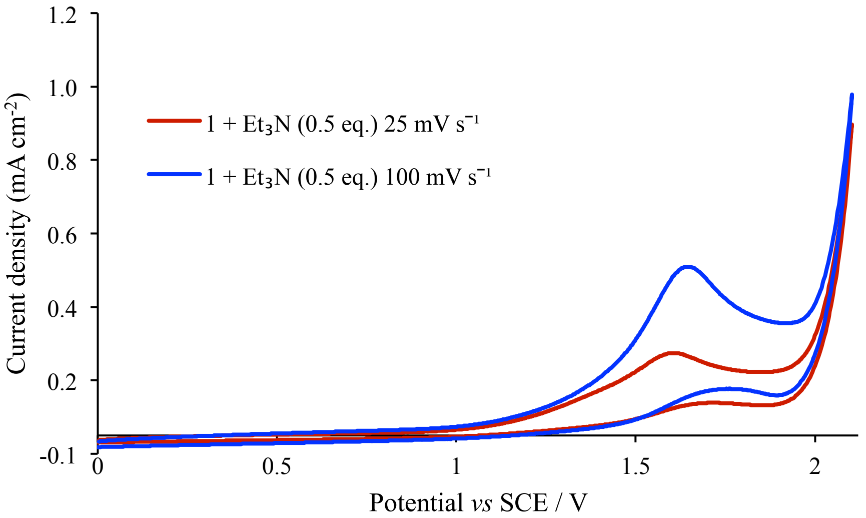

Figure S20: Cyclic voltammogram for 5 mM cubane carboxylic acid (**1**) and 2.5 mM Et_3N in MeOH/0.1 M Et_4NBF_4. Red line: Potential scan rate 25 mV s^{-1}; Blue line: Potential scan rate 100 mV s^{-1}

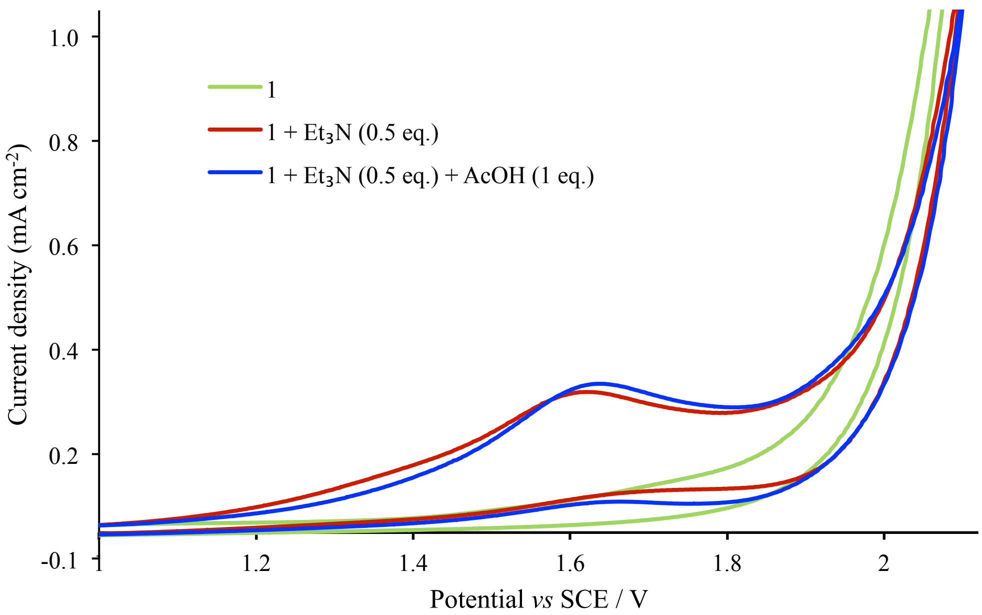

Figure S21: Cyclic voltammogram for 5 mM cubane carboxylic acid (**1**), 2.5 mM Et_3N and 5 mM AcOH in MeOH/0.1 M Et_4NBF_4. Potential scan rate 25 mV s^{-1}.

7 Synthetic procedures

7.1 General procedure A: Electrolysis with platinum electrode

To a 10 mL volumetric flask, containing a solution of 4-methoxycarbonyl-1-cubanecarboxylic acid (**1**, 206 mg, 1.0 mmol, 1 equiv.) in the corresponding solvent, AcOH (60 µL, 1.0 mmol, 1 equiv.) and Et_3N (68 µL, 0.50 mmol, 0.5 equiv.) were added. The solution was stirred until homogenous and pumped through the Ammonite 8 reactor (internal volume = 1 mL) with a fixed flow rate of 0.2 mL min^{-1} and an applied current of 200 mA (which for these reactions lead to 6.2 F). After all the solution was passed through the reactor, the corresponding solvent was pumped through the reactor for 15 minutes. The solvent was removed under reduced pressure, and the crude reaction mixture was purified by column chromatography.

7.2 General procedure B: Electrolysis with Carbon/PVDF electrode

To a 10 mL volumetric flask, containing a solution of 4-methoxycarbonyl-1-cubanecarboxylic acid (**1**, 206 mg, 1.00 mmol, 1.00 equiv.) in the corresponding solvent, Et_3N (68 µL, 0.50 mmol, 0.50 equiv.) was added. The solution was stirred until homogenous and pumped through the Ammonite 8 reactor (internal volume = 1 mL) with a fixed flow rate of 0.5 mL min^{-1} and an applied current of 200 mA (which for these reactions lead to 2.5 F). After all the solution was passed through the reactor, the corresponding solvent was pumped through the reactor for 5 minutes. The solvent was removed under reduced pressure, and the crude reaction mixture was purified by column chromatography.

7.3 Gramme-scale synthesis of methyl 4-methoxy-1-cubanecarboxylate (7)

To a 250 mL round-bottom flask, containing a solution of 4-methoxycarbonyl-1-cubanecarboxylic acid (**1**, 2.57 g, 12.5 mmol, 1.00 equiv.) in MeOH (125 mL) were added Et_3N (0.87 mL, 6.3 mmol, 0.50 equiv.). The solution was stirred until homogenous and pumped through the Ammonite 8 reactor with a flow rate of 0.5 mL min^{-1} (internal volume = 1 mL, theoretical residence time t_R = 2 min) with an applied current of 200 mA using a C/PVDF anode. After all the solution had passed through the reactor, pure methanol was passed through the reactor for 5 min. The solvent was removed under reduced pressure and purification was achieved via flash column chromatography (9:1 pentane/Et_2O) to afford methyl 4-methoxy-1-cubanecarboxylate (**7**, 1.08 g, 5.62 mmol, 45%) as an off-white solid and methyl cubanecarboxylate (**10**, 152 mg, 0.937 mmol, 8%) as a colourless solid.

NOTE: During the large-scale reaction, the voltage in the cell slowly but steadily increased with time due to a small deposit on the platinum electrode. When the voltage reached ≈ 10 V (usually between 2 – 4 V when the reaction starts), the starting material flask was switched with pure methanol and passed through the reactor for 5 minutes and collected in the same collection flask as the product. The Ammonite 8 reactor was then

disassembled, the platinum electrode was cleaned with a cotton wool and methanol to remove the deposit, and the cell was reassembled. The same above procedure was repeated until all the solution passed through the reactor.

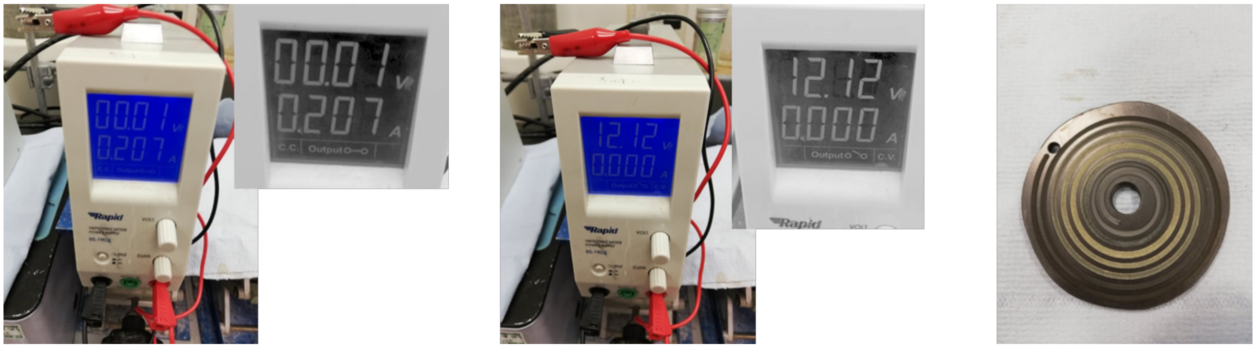

Figure S22: (Left) Selected current (200 mA). For the power supply used, the measured current ranged between 0.202–0.207 A. (Middle) Maximum selected voltage (12 V). (Right) Carbon/PVDF electrode after prolonged reaction.

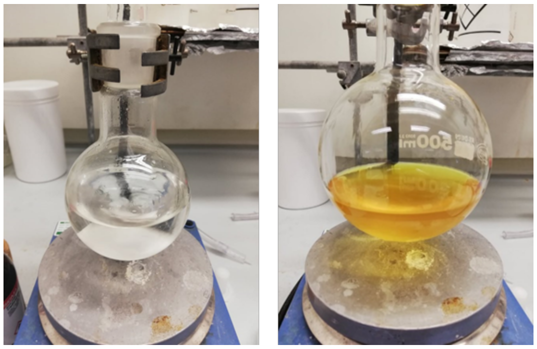

Figure S23: (Left) Solution of starting material **1**. (Right) Solution obtained after reaction.

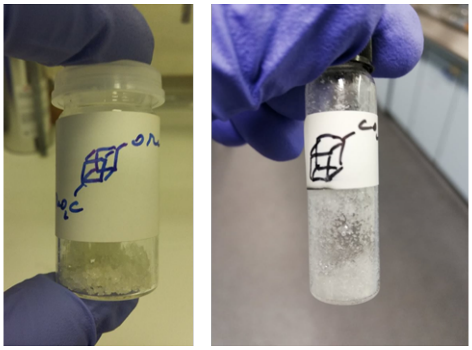

Figure S24: Obtained methyl 4-methoxy-1-cubane (**2**) and methyl cubanecarboxylate (**5**).

7.4 Methyl 4-methoxy-1-cubanecarboxylate (7)

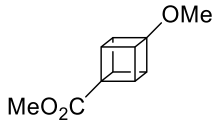

Prepared according to the **general procedure B**. The crude material was purified by flash column chromatography (9:1 pentane/Et_2O) to afford methyl 4-methoxy-1-cubanecarboxylate (**7**, 92 mg, 0.48 mmol, 48%) as an off-white solid. Data are consistent with those reported.[3]

Formula $C_{11}H_{12}O_3$;
MW 192.21 g.mol^{-1};
TLC R_f 0.19 (9:1 pentane/Et_2O);
mp 38 – 39 °C (Et_2O), [Lit.[3] 37 – 38 °C];
^{1}H NMR (400 MHz, $CDCl_3$) δ 4.15 – 4.21 (m, 3H), 3.97 – 4.03 (m, 3H), 3.71 (s, 3H), 3.34 (s, 3H) ppm;
^{13}C NMR (101 MHz, $CDCl_3$) δ 172.8 (*C*OOMe), 91.4 (*C*–OMe), 56.7 (*C*–COOMe), 51.5 (2C, COO*CH_3* + O*CH_3*), 50.5 (3C, CH cubyl), 42.5 (3C, CH cubyl) ppm;
IR 2986 (w), 2832 (w), 1700 (s), 1436 (m), 1296 (s), 1210 (m), 1020 (m), 836 (s), 570 (s) cm^{-1};
HRMS (ESI) m/z for $[C_{11}H_{13}O_3]^+$ $[M+H]^+$ calcd: 193.0859 found: 193.0861.

7.5 Methyl cubanecarboxylate (10)

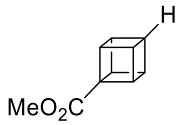

According the **general procedure B**, methyl cubanecarboxylate (**10**, 13 mg, 0.080 mmol, 8%) was isolated as a white solid. Data are consistent with those reported.[4]

Formula $C_{10}H_{10}O_2$;
MW 162.19 g.mol^{-1};
TLC R_f 0.74 (9:1 pentane/Et_2O);
^{1}H NMR (400 MHz, $CDCl_3$) δ 4.20 – 4.31 (m, 3H), 3.94 – 4.06 (m, 4H), 3.71 (s, 3H) ppm;
^{13}C NMR (101 MHz, $CDCl_3$) δ 172.8 (CO), 55.6 (*C*–$COOCH_3$), 51.4 (COO*CH_3*), 49.5 (3C, CH cubyl), 47.8 (CH cubyl), 45.1 (3C, CH cubyl) ppm.

7.6 Synthesis of methyl 4-methoxy(d_3)-1-cubanecarboxylate (11)

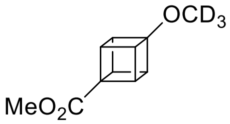

Prepared according to the **general procedure B**. The crude material was purified by flash column chromatography (9:1 pentane/Et_2O) to afford methyl 4-methoxy(d_3)-1-cubanecarboxylate (**11**, 79 mg, 0.40 mmol, 40%) as a white solid.

Formula $C_{11}H_9D_3O_3$;
MW 195.23 g.mol^{-1};
TLC R_f 0.19 (9:1 pentane/Et_2O);
mp 39 – 40 °C (hexane);
^{1}H NMR (400 MHz, $CDCl_3$) δ 4.15 – 4.20 (m, 3H), 3.97 – 4.02 (m, 3H), 3.71 (s, 3H) ppm;
^{13}C NMR (126 MHz, $CDCl_3$) δ 172.8 (*C*OOMe), 91.4 (*C*–OCD_3), 56.7 (*C*–COOMe), 51.5 (COOCH_3 + OCD_3), 50.6 (3C, CH cubyl), 42.5 (3C, CH cubyl) ppm;
IR 2988 (m), 2956 (w) 2847 (w), 1715 (s),1434 (m), 1304 (s br), 1207 (m), 1088 (m), 836 (s), 551 (s) cm^{-1};
HRMS (ESI) m/z for $[C_{11}H_{10}D_3O_3]^+$ $[M+H]^+$ calcd: 196.1048 found: 196.1046. $[M+Na]^+$ calcd: 218.0867 found: 218.0869.

7.7 Methyl 4-ethoxy-1-cubanecarboxylate (12)

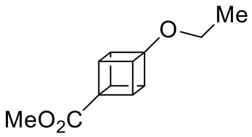

Prepared according to the **general procedure B** with C/PVDF anode. The crude material was purified by flash column chromatography (9:1 pentane/Et_2O) to afford methyl 4-ethoxy-1-cubanecarboxylate (**12**, 84 mg, 0.41 mmol, 41%) as a yellowish-white solid.

Formula $C_{12}H_{14}O_3$;
MW 206.24 g.mol^{-1};
TLC R_f 0.27 (9:1 pentane/Et_2O);
^{1}H NMR (400 MHz, $CDCl_3$) δ 4.11 – 4.23 (m, 3H), 3.97 – 4.06 (m, 3H), 3.71 (s, 3H), 3.54 (q, J = 7.1 Hz, 2H), 1.27 (t, J =7.0 Hz, 3H) ppm;
^{13}C NMR (101 MHz, $CDCl_3$) δ 172.9 (*C*OOMe), 90.6 (*C*–OCH_2CH_3), 59.9 (*C*H_2CH_3), 56.6 (*C*–COOMe), 51.5 (COOCH_3), 51.2 (3C, CH cubyl), 42.6 (3C, CH cubyl), 15.4 (CH_2*C*H_3) ppm;

IR 2974 (m), 2873 (w), 1722 (s), 1303 (s), 1124 (s), 1045 (s) cm^{-1};
HRMS (ESI) m/z for $[C_{12}H_{15}O_3]^+$ $[M+H]^+$ calcd: 207.1016 found: 207.1016.

7.8 Methyl 4-isopropoxy-1-cubanecarboxylate (13)

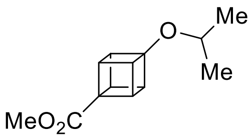

Prepared according to the **general procedure B** with C/PVDF anode. The crude material was purified by flash column chromatography (9:1 pentane/Et_2O) to afford methyl 4-isopropoxy-1-cubanecarboxylate (**13**, 19 mg, 0.086 mmol, 9%) as an off-white solid.

Formula: $C_{13}H_{16}O_3$;
MW 220.27 $g.mol^{-1}$;
TLC R_f = 0.18 (9:1 pentane/Et_2O);
^{1}**H NMR** (400 MHz, $CDCl_3$) δ 4.10 – 4.18 (m, 3H), 3.97 – 4.04 (m, 3H), 3.90 (spt, *J* = 6.2 Hz, 1H), 3.70 (s, 3H), 1.20 (d, *J* = 6.2 Hz, 6H) ppm;
^{13}**C NMR** (101 MHz, $CDCl_3$) δ 172.9 (*C*OOMe), 90.1 (*C*–OCH(CH_3)$_2$), 68.4 (*CH*(CH_3)$_2$), 56.4 (*C*–COOMe), 52.4 (3C, CH cubyl), 51.5 (COO*CH$_3$*), 42.7 (3C, CH cubyl), 23.5 (2C, CH(*C*H_3)$_2$) ppm;
IR 2978 (m), 1724 (s), 1296 (s), 1128 (m), 1088 (m) cm^{-1};
HRMS (ESI) m/z for $[C_{13}H_{17}O_3]^+$ $[M+H]^+$ calcd: 221.1172 found: 221.1174.

7.9 Methyl 4-trifluoroethoxy-1-cubanecarboxylate (14)

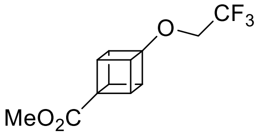

Prepared according to the **general procedure A**. The crude material was purified by flash column chromatography (hexane/Et_2O gradient from 10:1 to 9:1) to afford methyl 4-trifluoroethoxy-1-cubanecarboxylate (**14**, 62 mg, 0.24 mmol, 24%) as a yellow solid.

NOTE: purification of fluorinated alkoxy-cubanes required repeated column chromatography.

Formula $C_{12}H_{11}F_3O_3$;
MW 260.21 $g.mol^{-1}$;
TLC R_f 0.14 (9:1 hexane/Et_2O);
^{1}**H NMR** (400 MHz, $CDCl_3$) δ 4.18 – 4.24 (m, 3H), 4.01 – 4.07 (m, 3H), 3.86 (q, *J* = 8.7 Hz, 2H), 3.71 ppm (s, 3H) ppm;

^{19}F NMR (376 MHz, CDl_3) δ –74.41 (t, *J* = 8.7 Hz, CF_3) ppm;
^{13}C NMR (101 MHz, $CDCl_3$) δ 172.4 (*C*OOMe), 123.6 (q, *J* = 278.0 Hz, CF_3), 91.4 (*C*–OCH_2CF_3), 62.7 (q, *J* = 34.5 Hz, CH_2), 56.6 (*C*–COOMe), 51.6 (COO*CH_3*) 51.0 (3C, CH cubyl), 42.5 (3C, CH cubyl) ppm;
IR 2988 (w), 2957 (w), 1712 (s), 1437 (m), 1263 (s), 1162 (s), 961 (s), 837 (s), 691 (s) cm^{-1};
HRMS (ESI) m/z for $[C_{12}H_{11}F_3O_3Na]^+$ $[M+Na]^+$ calcd: 283.0552 found: 283.0558.

7.10 Methyl 4-(1,1,1,3,3,3-hexafluoroisopropoxy)-1-cubanecarboxylate (15)

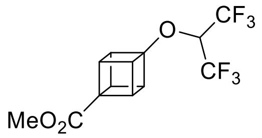

Prepared according to the **general procedure A**. The crude material was purified by flash column chromatography (hexane/Et_2O gradient from 10:1 to 9:1) to afford methyl 4-(1,1,1,3,3,3-hexafluoroisopropoxy)-1-cubanecarboxylate (**15**, 89 mg, 0.27 mmol, 27%) as a white solid.

NOTE: purification of fluorinated alkoxy-cubanes required repeated column chromatography.

Formula $C_{13}H_{10}F_6O_3$;
MW 328.21 $g.mol^{-1}$;
TLC R_f 0.22 (9:1 hexane/Et_2O);
mp 103 – 105 °C (HFIP);
^{1}H NMR (400 MHz, $CDCl_3$) δ 4.22 – 4.29 (m, 3H), 4.19 (spt, *J* = 6.0 Hz, 1H), 4.03 – 4.12 (m, 3H), 3.72 (s, 3H) ppm;
^{19}F NMR (376 MHz, $CDCl_3$) δ –73.74 (6F, d, *J* = 5.2 Hz, 2 CF_3) ppm;
^{13}C NMR (126 MHz, $CDCl_3$) δ 172.1 (*C*OOMe), 121.0 (q, *J* = 283.2 Hz, CF_3), 121.0 (q, *J* = 282.9 Hz, CF_3), 93.1 (C–O), 71.6 (spt, *J* = 33.1 Hz, *$CH(CF_3)_2$*), 56.6 (*C*–COOMe), 51.7 (3C, CH cubyl), 51.6 (COO*CH_3*), 42.4 (3C, CH cubyl) ppm;
IR 2995 (w), 2949 (w), 1716 (s), 1377 (s), 1266 (s), 1191 (m), 1087 (m), 878 (s), 686 (s) cm^{-1};
HRMS (ESI) m/z for $[C_{13}H_{11}F_6O_3]^+$ $[M+H]^+$ calcd: 283.0607 found: 283.0605.

7.11 Synthesis of (4-methoxycubyl)(piperidin-1-yl)methanone (16)

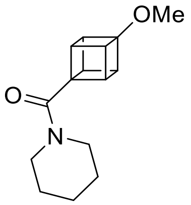

Prepared according to the **general procedure B** with C/PVDF anode. The crude material was purified by flash column chromatography (6:4 hexane/EtOAc) to afford (4-methoxycubyl)(piperidin-1-yl)methanone (**16**, 139 mg, 0.566 mmol, 57%) as an off-white solid.

Formula $C_{15}H_{19}NO_2$;
MW 245.32 g.mol^{-1};
TLC R_f 0.13 (6:4 hexane/EtOAc);
^{1}H NMR (400 MHz, $CDCl_3$) δ 4.11 – 4.20 (m, 3H), 3.92 – 4.01 (m, 3H), 3.46 – 3.56 (m, 2H), 3.33 (s, 3H), 3.13 – 3.24 (m, 2H), 1.60 – 1.70 (m, 2H), 1.48 – 1.60 (m, 4H) ppm;
^{13}C NMR (101 MHz, $CDCl_3$) δ 169.9 (CON), 90.6 (*C*–OMe), 59.2 (*C*–CON), 51.4 (OCH_3), 49.9 (3C, CH cubyl), 45.7 (CH_2), 42.7 (CH_2), 42.5 (3C, CH cubyl), 26.8 (CH_2), 25.4 (CH_2), 24.6 (CH_2) ppm;
IR 2977 (w), 2936 (br), 1611 (s), 1441 (s), 1289 (s), 1016 (m), 728 (m) cm^{-1};
HRMS (ESI) m/z for $[C_{15}H_{20}NO_2]^+$ $[M+H]^+$ calcd: 246.1489 found: 246.1489; for $[C_{15}H_{19}NNaO_2]^+$ $[M+Na]^+$ calcd: 268.1308 found: 268.1307.

7.12 Synthesis of *N,N*-diethyl-4-methoxycubane-1-carboxamide (17)

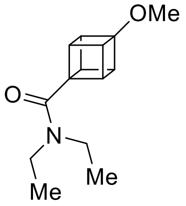

Prepared according to the **general procedure B** with C/PVDF anode. The crude material was purified by flash column chromatography (6:4 hexane/EtOAc) to afford *N,N*-diethyl-4-methoxycubane-1-carboxamide (**17**, 138 mg, 0.591 mmol, 59%) as a colourless oil.

Formula $C_{14}H_{19}NO_2$;
MW 233.31 g.mol^{-1};
TLC R_f 0.42 (6:4 hexane/EtOAc);
^{1}H NMR (400 MHz, $CDCl_3$) δ 4.11 – 4.19 (m, 3H), 3.93 – 4.01 (m, 3H), 3.35 (q, *J* = 7.1 Hz, 2H), 3.33 (s, 3H), 3.14 (q, *J* = 7.1 Hz, 2H), 1.19 (t, *J* = 7.1 Hz, 3H), 1.11 (t, *J* = 7.1 Hz, 3H) ppm;

^{13}**C NMR** (101 MHz, $CDCl_3$) δ 170.9 (CON), 90.7 (*C*–OMe), 59.5 (*C*–CON), 51.4 (OCH_3), 49.8 (3C, CH cubyl), 42.7 (3C, CH cubyl), 40.6 ($\mathit{CH_2}CH_3$), 39.1 ($\mathit{CH_2}CH_3$), 14.6 ($CH_2\mathit{CH_3}$), 12.8 ($CH_2\mathit{CH_3}$) ppm;

IR 2979 (s), 2831 (w), 1622 (s), 1442 (w), 1313 (s), 1294 (w), 1018 (m) cm^{-1};

HRMS (ESI) m/z for $[C_{14}H_{20}NO_2]^+$ $[M+H]^+$calcd: 234.1489 found: 234.1485; for $[C_{14}H_{19}NNaO_2]^+$ $[M+Na]^+$calcd: 256.1308 found: 256.1303.

7.13 Synthesis of *N,N*-diisopropyl-4-methoxycubane-1-carboxamide (18)

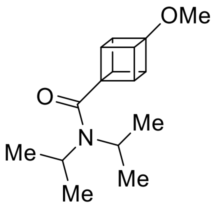

Prepared according to the **general procedure B** with C/PVDF anode and 4-(diisopropylcarbamoyl)cubane-1-carboxylic acid (**S15**, 254 mg, 0.90 mmol, 1.0 equiv.), Et_3N (62 μL, 0.45, 0.5 equiv.) in MeOH (9 mL). The crude material was purified by flash column chromatography (6:4 hexane/EtOAc) to afford *N,N*-diisopropyl-4-methoxycubane-1-carboxamide (**18**, 144 mg, 0.551 mmol, 60%) as a beige solid.

Formula $C_{16}H_{23}NO_2$;

MW 261.37 g.mol^{-1};

TLC R_f 0.13 (7:3 hexane/EtOAc);

^{1}**H NMR** (400 MHz, $CDCl_3$) δ 4.10 – 4.16 (m, 3H), 3.90 – 3.96 (m, 3H), 3.52 (spt, *J* = 6.7 Hz, 1H), 3.33 (s 1H), 3.29 (spt, *J* = 6.6 Hz, 1H), 1.41 (d, *J* = 6.8 Hz, 6H), 1.20 (d, *J* = 6.6 Hz, 6H) ppm;

^{13}**C NMR** (101 MHz, $CDCl_3$) δ 170.8 (CON), 90.6 (*C*–OMe), 60.4 (*C*–CON), 51.4 (OCH_3), 49.7 (3C, CH cubyl), 48.1 ($\mathit{CH}(CH_3)_2$), 45.8 ($\mathit{CH}(CH_3)_2$), 42.4 (3C, CH cubyl), 21.0 (2C, $CH(\mathit{CH_3})_2$), 20.5 (2C, $CH(\mathit{CH_3})_2$) ppm;

IR 2974 (m), 2932 (m), 2834 (w) 1618 (s), 1442 (s), 1308 (s), 1010, (s), 730 (m), 622 (m) cm^{-1};

HRMS (ESI) m/z for $[C_{16}H_{24}NO_2]^+$ $[M+H]^+$ calcd: 262.1802 found: 262.1806; for $[C_{16}H_{23}NNaO_2]^+$ $[M+Na]^+$ calcd: 284.1621 found: 284.1621.

7.14 Synthesis of (4-methoxycubyl)(morpholino)methanone (19)

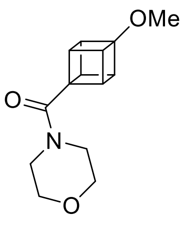

Prepared according to the **general procedure B** with C/PVDF anode and 4-(morpholine-4-carbonyl)cubane-1-carboxylic acid (S16) (**S16**, 210 mg, 0.80 mmol, 1.0 equiv.), Et_3N (56 µL, 0.40 mmol, 0.50 equiv.) in MeOH (8 mL). The crude material was purified by flash column chromatography (95:5 chloroform/methanol) to afford (4-methoxycubyl)(morpholino)methanone (**19**, 95 mg, 0.38 mmol, 48%) as a yellow solid.

Formula $C_{14}H_{17}NO_3$;
MW 247.29 g.mol^{-1};
TLC R_f (95:5 chloroform/methanol);
^{1}H NMR (400 MHz, $CDCl_3$) δ 4.14 – 4.21 (m, 3H), 3.95 – 4.04 (m, 3H), 3.65 – 3.70 (m, 5H), 3.56 – 3.63 (m, 2H), 3.34 (s, 3H), 3.24 – 3.30 (m, 2H) ppm;
^{13}C NMR (101 MHz, $CDCl_3$) δ 170.3 (CON), 90.5 (*C*–OMe), 66.9 (2C, OCH_2), 58.8 (*C*–CON), 51.5 (OCH_3), 50.0 (3C, CH cubyl), 45.2 (NCH_2), 42.4 (3C, CH cubyl), 41.9 (NCH_2) ppm;
IR 2989 (w), 2917 (w), 1610 (s), 1430 (m), 1112 (s), 1004 (s) cm^{-1};
HRMS (ESI) m/z for $[C_{14}H_{18}O_3]^+$ $[M+H]^+$ calcd: 248.1281 found: 248.1285.

7.15 Synthesis of 4-methoxy-1-cubanebenzylether (20)

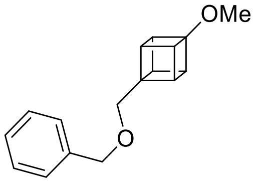

Prepared according to the **general procedure B** with C/PVDF anode and 4-benzyloxy-1-cubanecarboxylic acid **S11** (90 mg, 0.33 mmol, 1.00 equiv.), Et_3N (23 µL, 0.17 mmol, 0.50 equiv.) in MeOH (3.3 mL). The crude material was purified by flash column chromatography (9:1 hexane/EtOAc), followed by prep TLC (9:1 hexane/EtOAc) to afford 4-methoxy-1-cubanebenzylether (**20**, 17 mg, 0.067 mmol, 20%) as a white solid.

Formula $C_{17}H_{18}O_2$;
MW 254.33 g.mol^{-1};
TLC R_f 0.33 (9:1 hexane/EtOAc);
^{1}H NMR (400 MHz, $CDCl_3$) δ 7.28 – 7.39 (m, 5H), 4.57 (s, 2H), 4.06 – 4.13 (m, 3H), 3.62 – 3.67 (m, 3H), 3.61 (s, 2H), 3.34 (s, 3H) ppm;
^{13}C NMR (101 MHz, $CDCl_3$) δ 138.7 (Ar), 128.3 (2C, Ar), 127.6 (2C, Ar), 127.5 (Ar), 92.1 (*C*–OMe), 73.1 (Ar–CH_2), 70.9 (C_{cubyl}–CH_2), 58.5 (C_{cubyl}–CH_2), 51.2 (OCH_3), 49.9 (3C, CH cubyl), 40.5 (3C, CH cubyl) ppm;
IR 2978 (s), 2828 (m), 1451 (w), 1292 (s), 1071 (s), 1019 (s), 734 (m), 697 (s) cm^{-1};
HRMS (ESI) m/z for $[C_{17}H_{18}NaO_2]^+$ $[M+Na]^+$ calcd: 277.1199 found: 277.1197.

7.16 Synthesis of 4-methoxy-1-cubylmethanol (21)

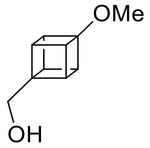

NOTE: 4-Hydroxymethyl-1-cubanecarboxylic acid is not soluble in methanol at a concentration of 0.1 M, therefore, 0.05 M concentration was used with a reduced current of 100 mA (instead of 200 mA) at the same flow rate to keep the charge constant (2.5 F).

Prepared according to the **general procedure B** with C/PVDF anode and 4-hydroxymethyl-1-cubanecarboxylic acid (99 mg, 0.56 mmol, 1.0 equiv.), and Et_3N (39 µL, 0.28 mmol, 0.5 equiv.) in MeOH (11 mL). The crude material was purified by flash column chromatography (6:4 pentane/Et_2O) to afford 4-methoxy-1-cubylmethanol (**21**, 42 mg, 0.26 mmol, 46%) as a white solid.

Formula $C_{10}H_{12}O_2$;
MW 164.20 g.mol^{-1};
TLC R_f 0.18 (6:4 pentane/Et_2O);
^{1}H NMR (400 MHz, $CDCl_3$) δ 4.05 – 4.14 (m, 3H), 3.79 (br s, 2H), 3.60 – 3.69 (m, 3H), 3.34 (s, 3H) ppm;
^{13}C NMR (101 MHz, $CDCl_3$) δ 92.2 (*C*–Me), 63.9 (CH_2), 59.7 (*C*-CH_2), 51.2 (OCH_3), 49.7 (3C, CH cubyl), 39.8 (3C, CH cubyl) ppm;
IR 3443 (br) 2974 (w), 2960 (w), 2844 (w), 1455 (w), 1285 (s), 1006 (s) cm^{-1};
LRMS (EI) m/z 163.9 for $C_{10}H_{12}O_2$ [M–H]$^-$.

7.17 Synthesis of *tert-butyl* 4-methoxy-1-cubanecarboxylate (22)

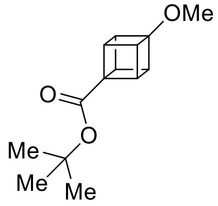

Prepared according to the **general procedure B** with C/PVDF anode and 4-*tert-but*oxycarbonyl-1-cubanecarboxylic acid (**S9**, 111 mg, 0.45 mmol, 1.0 equiv.), and Et_3N (31 µL, 0.23 mmol, 0.5 equiv.) in MeOH (4.5 mL). The crude material was purified by flash column chromatography (95:5 hexane/EtOAc) to afford *tert*-butyl 4-methoxy-1-cubanecarboxylate (**22**, 46 mg, 0.20 mmol, 44%) as a white solid.

Formula $C_{14}H_{18}O_3$;
MW 234.30 g.mol^{-1};
TLC R_f (95:5 hexane/EtOAc);

^{1}H NMR (400 MHz, $CDCl_3$) δ 4.10 – 4.17 (m, 3H), 3.89 – 3.96 (m, 3H), 3.33 (s, 3H), 1.46 (s, 9H) ppm;
^{13}C NMR (101 MHz, $CDCl_3$) δ 172.1 (*C*OO*t*Bu), 91.4 (*C*–OMe), 80.1 (O*C*(CH_3)$_3$), 57.8 (*C*–COO*t*Bu), 51.4 (O*CH_3*), 50.3 (3C, CH cubyl), 42.4 (3C, CH cubyl), 28.1 (3C, OC(*CH_3*)$_3$) ppm;
IR 2981 (s), 2932 (w), 1709 (s), 1304 (s), 1121 (s), 1018 (m), 837 (m), 568 (w) cm^{-1};
HRMS (ESI) m/z for $[C_{14}H_{18}NaO_3]^+$ $[M+Na]^+$ calcd: 257.1148 found: 257.1151.

7.18 Synthesis of 4-methoxy-1-cubanecarboxylic acid (24)

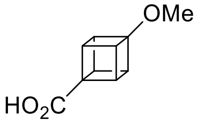

To a 25 mL flask, containing a solution of methyl 4-methoxy-1-cubanecarboxylate (**7**, 192 mg, 1.00 mmol, 1.0 equiv.) in THF (10 mL) was added NaOH (400 mg, 10.0 mmol, 10 equiv.) portionwise. After 16 h at room temperature, solvent was evaporated and the obtained solid was dissolved in 10 mL of water and washed with CH_2Cl_2 (3 × 5 mL). The aqueous phase was acidified with concentrated HCl (pH ≈ 1–2), and extracted with CH_2Cl_2 (4 × 5 mL). The combined organic extract was dried over $MgSO_4$ and concentrated under reduced pressure to afford 4-methoxy 1-cubanecarboxylic acid (**24**, 167 mg, 0.94 mmol, 94%) as a white powder.

Formula $C_{10}H_{10}O_3$;
MW178.19 g.mol^{-1};
TLC R_f n/a;
^{1}H NMR (400 MHz, $CDCl_3$) δ 4.17 – 4.25 (m, 3H), 4.01 – 4.09 (m, 3H), 3.35 (s, 3H) ppm;
^{13}C NMR (101 MHz, $CDCl_3$) δ 177.5 (COOH), 91.4 (*C*–OMe), 56.3 (*C*–COOH), 51.6 (OCH_3), 50.6 (3C, CH cubyl), 42.5 (3C, CH cubyl) ppm;
IR 2988 (w), 2980 (w), 2835 (w), 2848 (w), 2589 (w), 1667 (s), 1424 (m), 1294 (s), 1122 (m), 1010 (m) cm^{-1};
HRMS (ESI) m/z for $[C_{10}H_9O_3]^-$ $[M–H]^-$ calcd: 177.0557 found: 177.0561.

7.19 Synthesis of *tert*-butyl 4-methoxy-1-cubylcarbamate (23)

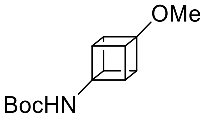

To a 50 mL flask, containing a solution of 4-methoxy-1-cubanecarboxylic acid (**24**, 500 mg, 2.81 mmol, 1.00 equiv.) in anhydrous *tert*-BuOH (10 mL) were added Et_3N (1.56 mL, 11.2 mmol, 4.00 equiv.) and diphenylphosphoryl azide (DPPA) (0.890 mL, 4.21 mmol, 1.50 equiv.). The resulting solution was heated under reflux for 14 h, then cooled to rt, and the solvent was removed under reduced pressure. The resulting brown oil was dissolved in EtOAc (25 mL) and washed with brine (3 × 10 mL). The combined organic phase was dried

over $MgSO_4$ and concentrated under reduced pressure. Purification by flash column chromatography (8:2 hexane/EtOAc) afforded *tert*-butyl 4-methoxy-1-cubylcarbamate (**23**, 483 mg, 1.94 mmol, 69%) as a white powder.

Formula $C_{14}H_{19}NO_3$;
MW 249.31 g.mol^{-1};
TLC R_f 0.34 (8:2 hexane /Et_2O);
^{1}H NMR (400 MHz, $CDCl_3$) δ 5.09 (br s, 1H), 4.03 (br s, 3H), 3.81 (br s, 3H), 3.33 (s, 3H), 1.46 (s, 9H) ppm;
^{13}C NMR (126 MHz, $CDCl_3$) δ 153.8 (NH*C*OOC(CH_3)$_3$), 92.0 (*C*–OMe), 79.8 (*C*–NHBoc), 67.3 (*C*(CH_3)$_3$), 51.3 (OCH_3), 47.8 (3C, CH cubyl), 45.5 (3C, CH cubyl), 28.3 (3C, C(*CH_3*)$_3$) ppm;
IR 3288 (m), 2991 (w), 2931 (w), 1675 (s), 1448 (w), 1521 (s), 1277 (s), 1166 (s), 1012 (s), 630 (m) cm^{-1};
HRMS (ESI+) m/z for $[C_{14}H_{20}O_3]^+$ $[M+H]^+$ calcd: 250.1438 found: 250.1440.

7.20 Synthesis of 4-methoxy-1-cubanamine hydrochloride (25)

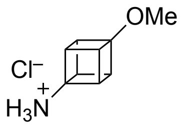

To a solution of *tert*-butyl 4-(methoxy)-1-cubylcarbamate (**23**, 670 mg, 2.69 mmol, 1.00 equiv.) in methanol (20 mL) at 0 °C was added a solution of HCl (6.75 mL of 4 N in 1,4-dioxane, 27.0 mmol, 10.0 equiv.) dropwise. The resulting solution was stirred for 7 h at rt. The solvent was concentrated under reduced pressure and the resulting solid was washed with Et_2O/acetone (4:1, 20 mL) to afford 4-methoxy-1-cubanamine hydrochloride (**25**, 456 mg, 2.46 mmol, 91%) as an off-white solid.

Formula $C_9H_{12}NOCl$;
MW 185.65 g.mol^{-1};
TLC Rf n/a;
^{1}H NMR (400 MHz, D_2O) δ 4.14 – 4.24 (m, 3H), 3.95 – 4.05 (m, 3H), 3.37 (s, 3H) ppm;
^{13}C NMR (101 MHz, D_2O) δ 91.6 (*C*–OMe), 65.4 (*C*–NH_3Cl), 51.4 (CH_3), 48.1 (3C, CH cubyl), 43.1 (3C, CH cubyl) ppm;
IR 3487 (w), 2905 (br), 1330 (s), 1318 (w), 1014 (s) cm^{-1}.

7.21 Synthesis of 4-methoxy-1-cubancarbaldehyde (27)

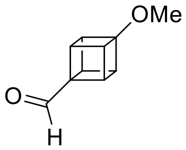

To a solution of 4-methoxy-1-cubylmethanol (**21**, 100 mg, 0.610 mmol, 1 equiv.) in CH_2Cl_2 (7 mL) was added Dess–Martin periodinane (0.284 g, 0.67 mmol, 1.1 equiv.) portionwise. The solution was stirred for 1 h, diluted with Et_2O (1.5 mL), poured into a saturated solution of $NaHCO_3$ containing sodium thiosulfate (0.611 g, 3.88 mmol, 6.4 equiv.), and stirred vigorously for 10 min. The phases were separated and the organic phase was washed with H_2O (3 × 2 mL), dried over $MgSO_4$ and concentrated under reduced pressure. Purification by flash column chromatography (8:2 pentane/Et_2O) afforded 4-methoxy-1-cubacarbaldehyde (**27**, 69 mg, 0.42 mmol, 69%) as a white powder.

Formula $C_{10}H_{10}O_2$;
MW 162.19 g.mol^{-1};
TLC R_f 0.39 (8:2 pentane/Et_2O);
^{1}H NMR (400 MHz, $CDCl_3$) δ 9.78 (1H, s), 4.16 – 4.23 (m, 3H), 4.10 – 4.16 (m, 3H), 3.35 (s, 3H) ppm;
^{13}C NMR (126 MHz, $CDCl_3$) δ 198.4 (CHO), 91.4 (*C*–OMe), 63.6 (*C*–CHO), 51.5 (CH_3), 50.7 (3C, CH cubyl), 41.3 (3C, CH cubyl) ppm;
IR 2995 (w), 2829 (w), 1681 (s), 1455 (m), 1283 (s), 1120 (m), 1013 (s) cm^{-1};
HRMS (ESI) m/z for $[C_{10}H_{11}O_2]^+$ $[M+H]^+$ calcd: 163.0754 found: 163.0755.

7.22 Synthesis of Cubanisindione (29)

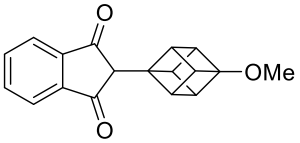

To a solution of 4-methoxy-1-cubancarbaldehyde **27** (114.0 mg, 0.703 mmol, 1.00 equiv.) and phthalide (**28**, 95 mg, 0.71 mmol, 1.00 equiv.) in EtOAc (2 mL) under N_2 atmosphere, was added a solution of NaOMe in MeOH (0.640 mL of 25 wt%, 2.82 mmol, 4.00 equiv.). The resulting solution was stirred at 40 °C for 3 h, then cooled to rt and concentrated under reduced pressure. The resulting red oil was dissolved in water (10 mL) and the aqueous solution was acidified with HCl (2 N) (pH ≈ 3). The aqueous phase was extracted with $CHCl_3$ (3 × 10 mL), dried over $MgSO_4$, and the solvent was removed under reduced pressure. Purification by flash chromatography (8:2 hexane/EtOAc), and recrystallisation from Et_2O, afforded cubanisindione (**29**, 65.0 mg, 0.24 mmol, 33%) as an off-white powder.

Formula $C_{18}H_{14}O_3$;

MW 278.31 g.mol^{-1};

TLC R_f 0.21 (8:2 hexane/EtOAc);

^{1}H NMR (400 MHz, $CDCl_3$) δ 7.97 (dd, *J* = 5.7, 3.1 Hz, 2H), 7.85 (dd, *J* = 5.6, 3.1 Hz, 2H), 4.07 – 4.14 (m, 3H), 3.80 – 3.87 (m, 3H), 3.31 (s, 3H), 3.29 (s, 1H) ppm;

^{13}C NMR (101 MHz, $CDCl_3$) δ 198.9 (2C, CO), 142.6 (2C, C_{Ar}), 135.7 (2C, CH_{Ar}), 123.1 (2C, CH_{Ar}), 92.1 (*C*–OMe), 57.5 (*C*CHCO), 55.3 (C*CH*CO), 51.3 (OCH_3), 50.5 (3C, CH cubyl), 41.8 (3C, CH cubyl) ppm;

IR 2985 (w), 2929 (w), 1706 (s), 1321 (m), 1253 (s) cm^{-1};

HRMS (ESI) *m/z* for $[C_{18}H_{14}NaO_3]^+$ $[M+Na]^+$ calcd: 301.0835 found: 301.0840

7.23 Synthesis of 4-methoxy-1-cubylmethanol (21)

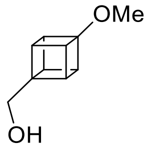

To a 50 mL flame-dried flask, methyl 4-methoxy-1-cubanecarboxylic acid (**24**, 500 mg, 2.81 mmol, 1.00 equiv.) was dissolved in anhydrous THF (15 mL) and cooled to 0 °C using an ice-bath. Afterwards, a solution of BH_3•SMe_2 (0.6 mL of 5 M in Et_2O, 3.0 mmol, 1.1 equiv.) was added dropwise and stirred for 2 h at rt. The reaction mixture was quenched carefully with H_2O (2 mL) and diluted with Et_2O (14 mL). The organic phase was washed with H_2O (2 × 14 mL) and brine (14 mL), dried over $MgSO_4$ and concentrated under reduced pressure. Purification was achieved *via* flash column chromatography (6:4 pentane/Et_2O) to afford 4-methyl-1-cubylmethanol (**21**, 380 mg, 2.31 mmol, 82%) as a white powder. See above for characterisation data (Section 7.16).

7.24 Synthesis of *tert*-butyl 4-methyl cubanedicarboxylate (S1)

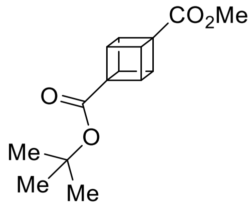

Following a procedure adapted from Ma *et al.*[5] To a solution of 4-methoxycarboxyl-1-cubanecarboxylic acid (**1**, 412 mg, 1.99 mmol, 1.00 equiv.) in anhydrous DMF (3 mL) and anhydrous *tert*-butanol (1.9 mL, 20.0 mmol, 10.0 equiv.) were added *N*-(3dimethylaminopropyl)-*N′*-ethylcarbodiimide (EDC) (575 mg, 2.99 mmol, 1.50 equiv.), 4-dimethylaminopyridine (DMAP, 244 mg. 1.99 mmol, 1.00 equiv.) and the mixture was stirred for 20 h at rt. Then, the resulting mixture was diluted with EtOAc (10 mL) and the organic phase was washed with aqueous HCl (4 mL of 1N), saturated $NaHCO_3$ (4 mL), H_2O (5 × 10 mL), dried over $MgSO_4$ and concentrated

under reduced pressure to afford *tert*-butyl 4-methyl cubanedicarboxylate (**S1**, 219 mg, 0.835 mmol, 42%) as a white powder. The *tert*-butyl ester **S1** was used in the next step without further purification.

Formula $C_{15}H_{18}O_4$;
MW 262.31 g.mol^{-1};
^{1}H NMR (400 MHz, $CDCl_3$) δ 4.14 – 4.23 (m, 6H), 3.72 (s, 3H), 1.47 (s, 9H) ppm;
^{13}C NMR (101 MHz, $CDCl_3$) δ 172.2 (*C*OOMe), 171.2 (*C*OO*t*Bu), 80.4 (O*C*(CH_3)$_3$), 56.9 (*C*–COO*t*Bu), 55.8 (*C*–COOMe), 51.6 (COO*CH_3*), 47.0 (3C, CH cubyl), 46.9 (3C, CH cubyl), 28.1 (3C, OC(*CH_3*)$_3$) ppm;
IR 2985 (s), 2952 (w), 1707 (s), 1321 (s), 1228 (s), 1086 (s), 862 (m), 731 (w) cm^{-1};
HRMS (ESI) *m/z* for $[C_{15}H_{18}NaO_4]^+$ $[M+Na]^+$ calcd: 285.1097 found: 285.1104.

7.25 Synthesis of methyl 4-hydroxymethyl-1-cubanecarboxylate (S2)

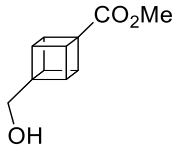

Following a procedure adapted from Burton *et al.*[6] To a 25 mL flame-dried flask, 4-methoxycarboxyl-1-cubanecarboxylic acid (**1**, 412 mg, 1.99 mmol, 1.00 equiv.) was dissolved in anhydrous THF (12 mL) and cooled to 0 °C using an ice-water bath. BH_3•SMe_2 solution (0.60 mL of 5 M in Et_2O, 3.0 mmol, 1.5 equiv.) was added dropwise, and the resulting solution was stirred for 2 h at rt. The reaction mixture was quenched by careful addition of H_2O (2 mL) and diluted with Et_2O (12 mL). The organic phase was washed with H_2O (2 × 5 mL), brine (5 mL), dried over $MgSO_4$ and concentrated under reduced pressure. Purification by flash column chromatography (1:1 hexane/EtOAc) afforded methyl 4-hydroxymethyl-1-cubanecarboxylate (**S2**, 336 mg, 1.75 mmol, 88%) as a white powder. Data are consistent with the literature.[6]

Formula $C_{11}H_{12}O_3$;
MW 192.21 g.mol^{-1};
TLC R_f 0.24 (1:1 hexane/EtOAc);
^{1}H NMR (400 MHz, $CDCl_3$) δ 4.11 – 4.19 (m, 3H), 3.85 – 3.93 (m, 3H), 3.78 (s, 2H), 3.71 (s, 3H), 1.39 (br s, 1H) ppm;
^{13}C NMR (101 MHz, $CDCl_3$) δ 172.6 (*C*OOMe), 63.3 (CH_2), 58.8 (*C*–CH_2), 56.4 (*C*–COOMe), 51.5 (COO*CH_3*), 46.3 (3C, CH cubyl) 44.4 (3C, CH cubyl) ppm;
IR 3288 (br), 2984 (w), 2859 (w), 1716 (s), 1440 (s), 1304 (s), 1198 (s), 1091 (s), 1027 (s), 999 (s) cm^{-1}.

7.26 Synthesis of methyl 4-benzyloxymethyl-1-cubanecarboxylate (S3)

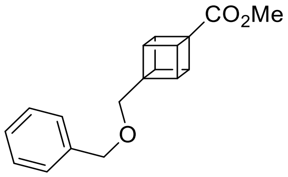

To a solution of methyl 4-hydroxymethyl-1-cubanecarboxylate (**S2**, 300 mg, 1.56 mmol, 1.0 equiv.) in anhydrous THF (5 mL) were added benzyl bromide (0.280 mL, 2.34 mmol, 1.5 equiv.) sodium hydride (125 mg of 60% in mineral oil, 3.12 mmol, 2.0 equiv.). The mixture was stirred for 18 h under argon atmosphere, then carefully quenched with saturated $NaHCO_3$ (2 mL) and diluted with H_2O (5 mL). The organic phase was extracted with Et_2O (3 × 5 mL), dried over $MgSO_4$ and concentrated under reduced pressure. Purification by flash column chromatography (7:3 hexane/EtOAc) afforded methyl 4-benzyloxymethyl-1-cubanecarboxylate (**S3**, 127 mg, 0.449 mmol, 29%) as a white powder.

Formula $C_{18}H_{18}O_3$;
MW 282.34 g.mol^{-1};
TLC R_f 0.38 (7:3 hexane/EtOAc);
^{1}H NMR (400 MHz, $CDCl_3$) δ 7.28 – 7.39 (m, 5H), 4.56 (s, 2H), 4.12 – 4.19 (m, 3H), 3.85 – 3.92 (m, 3H), 3.71 (s, 3H), 3.60 (s, 2H) ppm;
^{13}C NMR (101 MHz, $CDCl_3$) δ 172.7 (*C*OOMe), 138.5 (Ar), 128.4 (2C, Ar), 127.6 (2C, Ar) 127.6 (Ar), 73.2 (Ar–*CH₂*), 70.4 (C_{cubyl}–CH_2), 57.6 (C_{cubyl}–CH_2), 56.2 (*C*–COOMe), 51.5 (COO*CH₃*), 46.6 (3C, CH cubyl), 45.2 (3C, CH cubyl) ppm;
IR 2973 (m), 2854 (m), 1715 (s), 1305 (m), 1207 (s), 1068 (s), 748 (m), 594 (m) cm^{-1};
HRMS (ESI) *m/z* for $[C_{16}H_{18}NaO_3]^+$ $[M+Na]^+$ calcd: 305.1148 found: 305.1148.

7.27 Synthesis of methyl 4-((*tert*-butoxycarbonyl)amino)cubane-1-carboxylate (S4)

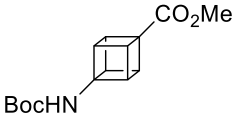

Following a procedure adapted from Nicolaou *et al.*[7] To a solution of 4-methoxycarboxyl-1-cubanecarboxylic acid (**1**, 500 mg, 2.42 mmol, 1.0 equiv.) in anhydrous *tert*-BuOH (15 mL) under an argon atmosphere, were added Et_3N (1.34 mL, 9.66mmol, 4.00 equiv.) and diphenylphosphoryl azide (DPPA, 0.77 mL, 3.6 mmol, 1.5 equiv.). The mixture was heated under reflux for 12 h, then the solvent was removed under reduced pressure and the resulting crude material was disolved in EtOAc (20 mL). The solution was washed with brine (3 × 10 mL), dried over $MgSO_4$ and concentrated under reduced pressure. Purification by flash column chromatography (1:0

to 4:1 hexane/EtOAc) afforded methyl 4-((*tert*-butoxycarbonyl)amino)cubane-1-carboxylate (**S4**, 322 mg, 1.16 mmol, 48%) as a white powder. Data are consistent with the literature.[7]

Formula $C_{15}H_{19}NO_4$;
MW 277.32 g.mol^{-1};
TLC R_f (8:2 hexane/EtOAc);
^{1}H NMR (500 MHz, $CDCl_3$) δ 5.09 (br s, 1H), 4.10 (br s, 6H), 3.71 (s, 3H), 1.46 (s, 9H) ppm;
^{13}C NMR (126 MHz, $CDCl_3$) δ 172.7 (*C*OOMe), 153.9 (NH*C*OOtBu), 79.9 (O*C*(CH_3)$_3$), 66.3 (*C*–NHBoc), 56.0 (*C*–COOMe), 51.5 (COO*CH$_3$*), 50.2 (3C, CH cubyl), 44.5 (3C, CH cubyl), 28.3 (3C, C(*CH$_3$*)$_3$) ppm;
IR 3395 (s), 2983 (w), 2951 (w), 1724 (s), 1689 (s), 1496 (s), 1313 (s), 1201 (s), 1087 (s) cm^{-1}.

7.28 General procedure C for the synthesis of amides

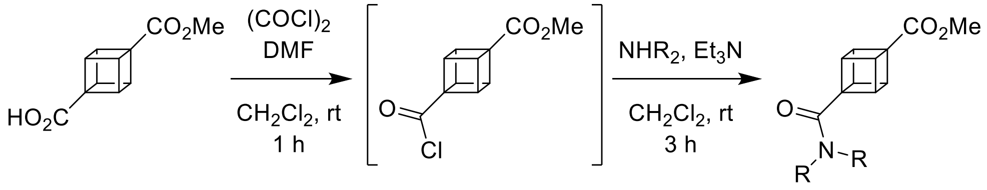

To a 25 mL flame-dried flask, containing a solution of 4-methoxycarbonyl-1-cubanecarboxylic acid (**1**, 412 mg, 1.99 mmol, 1.00 equiv.) in anhydrous CH_2Cl_2 (10 mL) were added oxalyl chloride (0.26 mL, 3.0 mmol, 1.5 equiv.) and 2 drops of anhydrous DMF. The solution was stirred for 1 h at rt. Then, the solution was concentrated under reduced pressure and the crude acyl chloride was dissolved in anhydrous CH_2Cl_2 (5 mL) and cooled to 0 °C. A solution of the corresponding amine (1.2 equiv.) and Et_3N (0.33 mL, 2.4 mmol, 1.2 equiv.) in anhydrous CH_2Cl_2 (2 mL) was transferred dropwise to the acyl chloride solution, and the resulting solution was stirred under argon for 3 h. The reaction mixture was quenched carefully with HCl (5 mL of 1N,) and the aqueous phase was extracted with CH_2Cl_2 (3 × 5 mL), dried over $MgSO_4$ and concentrated under reduced pressure. The crude mixture was purified by column chromatography if required or used without further purification.

7.29 Synthesis of methyl 4-(piperidine-1-carbonyl)cubane-1-carboxylate (S5)

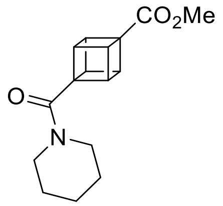

Prepared according to the **general procedure C** with piperidine. The crude material was purified by flash column chromatography (8:2 hexane/EtOAc) to afford methyl methyl 4-(piperidine-1-carbonyl)cubane-1-carboxylate (**S5**, 476 mg, 1.74 mmol, 87%) as a white solid.

Formula $C_{16}H_{19}NO_3$;
MW 273.33 g.mol^{-1};
TLC R_f (8:2 hexane/EtOAc);
^{1}**H NMR** (400 MHz, $CDCl_3$) δ 4.15 – 4.27 (m, 6H), 3.71 (s, 3H), 3.47 – 3.59 (m, 2H), 3.12 – 3.24 (m, 2H), 1.61 – 1.71 (m, 2H), 1.48 – 1.60 (m, 4H) ppm;
^{13}**C NMR** (101 MHz, $CDCl_3$) δ 172.2 (*C*OOMe), 169.1 (CON), 58.3 (*C*–CON), 54.7 (*C*–COOMe), 51.6 (COO*CH*$_3$), 47.0 (3C, CH cubyl), 46.4 (3C, CH cubyl), 45.9 (CH_2), 42.7 (CH_2), 26.7 (CH_2), 25.4 (CH_2), 24.6 (CH_2) ppm;

IR 2991 (m), 2936 (m), 2853 (m), 1724 (s), 1615 (s), 1435 (s), 1224 (s), 1087 (s), 729 (s) cm^{-1};

HRMS (ESI) m/z for $[C_{16}H_{20}NO_3]^+$ $[M+H]^+$ calcd: 274.1438 found: 274.1435; for $[C_{16}H_{19}NNaO_3]^+$ $[M+Na]^+$ calcd: 296.1257 found: 296.1255.

7.30 Synthesis of methyl 4-(diethylcarbamoyl)cubane-1-carboxylate (S6)

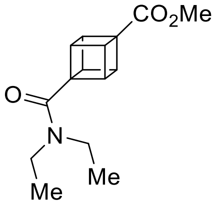

Prepared according to the **general procedure C** with diethylamine. The crude material was purified by flash column chromatography (8:2 hexane/EtOAc) to afford methyl methyl 4-(diethylcarbamoyl)cubane-1-carboxylate (**S6**, 471 mg, 1.80 mmol, 90%) as a white solid.

Formula $C_{15}H_{19}NO_3$;

MW 261.32 $g.mol^{-1}$;

TLC R_f (8:2 hexane/EtOAc);

^{1}H NMR (400 MHz, $CDCl_3$) δ 4.20 – 4.23 (m, 6H), 3.71 (s, 3H), 3.35 (q, *J* = 7.1 Hz, 2H), 3.13 (q, *J* = 7.1 Hz, 2H), 1.19 (t, *J* = 7.1 Hz, 3H), 1.12 (t, *J* = 7.1 Hz, 3H) ppm;

^{13}C NMR (101 MHz, $CDCl_3$) δ 172.2 (*C*OOMe), 170.1 (CON), 58.5 (*C*–CON), 54.8 (C–COOMe), 51.6 (COOCH_3), 47.2 (3C, CH cubyl), 46.4 (3C, CH cubyl), 40.8 (N$CH_2$$CH_3$), 39.2 (N$CH_2$$CH_3$), 14.6 ($NCH_2$$CH_3$), 12.8 ($NCH_2$$CH_3$) ppm;

IR 2972 (m), 2934 (w), 1716 (s), 1614 (s), 1434 (m), 1323 (s), 1211 (s), 1087 (s), 842 (w), cm^{-1};

HRMS (ESI) m/z for $[C_{15}H_{20}NO_3]^+$ $[M+H]^+$ calcd: 262.1438 found: 262.1436; for $[C_{15}H_{19}NNaO_3]^+$ $[M+Na]^+$ calcd: 284.1257 found: 284.1256.

7.31 Synthesis of methyl 4-(diisopropylcarbamoyl)cubane-1-carboxylate (S7)

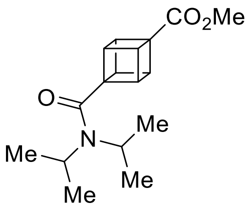

Prepared according to the **general procedure C** with diisopropylamine, but adapted to a larger scale using 4-methoxycarbonyl-1-cubanecarboxylic acid (**1**, 500 mg, 2.42 mmol). The crude material was purified by flash column chromatography (8:2 hexane/EtOAc) to afford methyl methyl 4-(diisopropylcarbamoyl)cubane-1-carboxylate (**S7**, 567 mg, 1.96 mmol, 81%) as a white solid. Data are consistent with the literature.[8]

Formula $C_{17}H_{23}NO_3$;
MW 289.38 g.mol^{-1};
TLC R_f 0.4 (8:2 hexane/EtOAc);
^{1}**H NMR** (400 MHz, $CDCl_3$) δ 4.26 – 4.14 (m, 6H), 3.72 (s, 3H), 3.48 (spt, *J* = 6.8 Hz, 1H), 3.32 (spt, *J* = 6.8 Hz, 1H), 1.43 (d, *J* = 6.7 Hz, 6H), 1.22 (d, *J* = 6.6 Hz, 6H) ppm;
^{13}**C NMR** (101 MHz, $CDCl_3$) δ 172.2 (*C*OOMe), 170.1 (CON), 59.4 (*C*–CON(*i*Pr)$_2$), 54.7 (*C*–COOMe), 51.6 (COOCH_3) 48.4 (*CH*(CH_3)$_2$), 47.0 (3C, CH cubyl), 46.2 (3C, CH cubyl), 45.90 (CH), 21.0 (2C, CH(CH_3)$_2$), 20.5 (2C, CH(CH_3)$_2$) ppm.

7.32 Synthesis of methyl 4-(morpholine-4-carbonyl)cubane-1-carboxylate (S8)

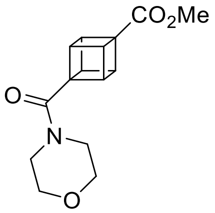

Prepared according to the **general procedure C** with morpholine. No further purification was carried out. Methyl 4-(morpholine-4-carbonyl)cubane-1-carboxylate (**S8**, 532 mg, 1.93 mmol, 97%) was obtained as a white solid.

Formula $C_{15}H_{17}NO_4$;
MW 275.30 g.mol^{-1};
TLC R_f n/a
^{1}**H NMR** (400 MHz, $CDCl_3$) δ 4.18 – 4.26 (m, 6H), 3.71 (s, 3H), 3.64 – 3.69 (m, 4H), 3.57 – 3.62 (m, 2H), 3.22 – 3.28 (m, 2H) ppm;
^{13}**C NMR** (101 MHz, $CDCl_3$) δ 171.9 (*C*OOMe), 169.5 (CON), 66.8 (OCH_2), 66.8 (OCH_2), 57.8 (*C*–CON), 54.7 (*C*–COOMe), 51.6 (COOCH_3), 46.9 (3C, CH cubyl), 46.5 (3C, CH cubyl), 45.4 (NCH_2), 41.9 (NCH_2) ppm;
IR 2954 (m), 2851 (m), 1713 (s), 1621 (s), 1424 (s), 1227 (s), 1111 (s), 1027 (s), 849 (s), 588 (s) cm^{-1};
HRMS (ESI) m/z for $[C_{15}H_{18}NO_4]^+$ $[M+H]^+$ calcd: 276.1230 found: 276.1225.

8 General procedure D for the synthesis of cubanecarboxylic acids

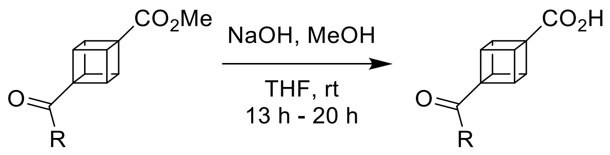

To a solution of the corresponding cubanecarboxylate ester in THF and powdered NaOH (between 1.0 – 30.0 equiv.) was added portionwise. After 12 h, the solvent was evaporated and the obtained solid was dissolved in the minimum amount of water and washed with CH_2Cl_2. The aqueous phase was acidified with concentrated HCl (pH ≈ 1 – 2) and extracted with CH_2Cl_2. The combined organic phase was dried over $MgSO_4$ and concentrated under reduced pressure to afford the cubanecarboxylic acid as a white powder. No further purification was carried out.

8.1 Synthesis of 4-*tert*-butoxycarbonyl-1-cubanecarboxylic acid (S9)

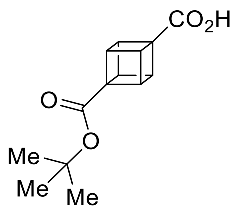

Prepared according to the **general procedure D** from 1-*tert*-butyl 4-methyl cubane-1,4-dicarboxylate (**S1**, 207 mg, 0.789 mmol, 1.00 equiv.) THF (8 mL) and NaOH (32 mg, 0.79 mmol, 1.0 equiv.). After extraction with CH_2Cl_2, the solution was dried over $MgSO_4$ and concentrated under reduced pressure to afford 4-*tert*-butoxycarbonyl-1-cubanecarboxylic acid (**S9**, 121 mg, 0.489 mmol, 62%) as a white solid.

Formula $C_{14}H_{17}NO_3$;
MW 247.28 g.mol^{-1};
TLC R_f n/a;

^{1}H NMR (400 MHz, $CDCl_3$) δ 4.23 – 4.28 (m, 3H), 4.17 – 4.22 (m, 3H), 1.47 (s, 9H) ppm;
^{13}C NMR (101 MHz, $CDCl_3$) δ 177.4 (*C*OOH), 171.1 (*C*OO*t*Bu), 80.6 (O*C*(CH_3)$_3$), 56.9 (*C*–COO*t*Bu), 55.5 (*C*–COOH), 47.1 (3C, CH cubyl), 46.8 (3C, CH cubyl), 28.1 (3C, OC(*CH$_3$*)$_3$) ppm;
IR 2990 (br), 2929 (w), 2607 (w), 1717 (s), 1675 (s), 1321 (s), 1160 (s), 1086 (s), 843 (s) cm^{-1};
HRMS (ESI) m/z for $[C_{14}H_{16}O_4]^-$ $[M–H]^-$ calcd: 247.0976 found: 247.0971.

8.2 Synthesis of 4-hydroxymethyl-1-cubanecarboxylic acid (S10)

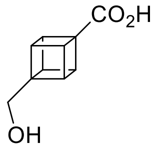

Prepared according to the **general procedure D** from methyl 4-hydroxymethyl-1-cubanecarboxylate (**S2**, 200 mg, 1.04 mmol, 1.00 equiv.), THF (10 mL) and NaOH (416 mg, 10.4 mmol, 10.0 equiv.). Extraction with EtOAc, drying over $MgSO_4$ and concentration under reduced pressure, afforded 4-hydroxymethyl-1-cubanecarboxylic acid (**S10**, 110 mg, 0.617 mmol, 62%) as a white solid.

Formula $C_{10}H_{10}O_3$;
MW 178.19 g.mol^{-1};
TLC R_f n/a;
^{1}H NMR (400 MHz, CD_3OD) δ 4.05 – 4.13 (m, 1H), 3.81 – 3.91 (3m, 3H), 3.67 (s, 2H) ppm;
^{13}C NMR (101 MHz, CD_3OD) δ 176.4 (COOH), 63.6 (CH_2), 60.6 (*C*–CH_2), 58.3 (*C*–COOH), 47.6 (3C, CH cubyl), 45.8 (3C, CH cubyl) ppm;
IR 3247 (br), 2991 (br), 2860 (w), 1655 (s), 1375 (s), 994 (s), 703 (w) cm^{-1}.

8.3 Synthesis of 4-benzyloxymethyl-1-cubanecarboxylic acid (S11)

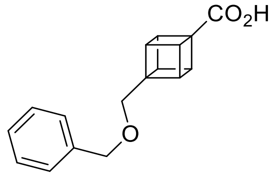

Prepared according to the **general procedure D** using methyl 4-benzyloxymethyl-1-cubanecarboxylate (**S3**, 114 mg, 0.404 mmol, 1.00 equiv.) THF (10 mL) and NaOH (488 mg, 12.2 mmol, 30.0 equiv.). Extraction with CH_2Cl_2, drying over $MgSO_4$ and concentration under reduced pressure afforded 4-benzyloxymethyl-1-cubanecarboxylic acid (**S11**, 101 mg, 0.376 mmol, 95%) as a white solid.

Formula $C_{17}H_{16}O_3$;
MW 268.31 g.mol^{-1};
TLC R_f n/a;
^{1}H NMR (400 MHz, $CDCl_3$) δ 7.27 – 7.39 (m, 5H), 4.57 (s, 2H), 4.16 – 4.23 (m, 3H), 3.87 – 3.94 (m, 3H), 3.61 (s, 2H) ppm;
^{13}C NMR (101 MHz, $CDCl_3$) δ 177.7 (COOH), 138.5 (*Ar*–CH_2), 128.4 (2C, Ar), 127.6 (3C, Ar), 73.2 (Ar–*CH_2*), 70.2 (C_{cubyl}–*CH_2*), 57.6 (*C_{cubyl}*–CH_2), 55.9 (*C*–$COOCH_3$), 46.6 (3C, CH cubyl), 45.2 (3C, CH cubyl) ppm;

IR 2987 (br), 2852 (br), 1681 (s), 1307 (m), 1205 (m), 1069 (s), 697 (s) cm^{-1};
HRMS (ESI) m/z for $[C_{17}H_{15}O_3]^-$ $[M–H]^-$ calcd: 267.1027 found: 267.1018.

8.4 Synthesis of 4-((*tert*-butoxycarbonyl)amino)cubane-1-carboxylic acid (S12)

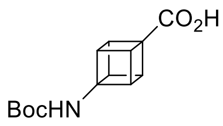

Prepared according to the **general procedure D** using methyl 4-((*tert*-butoxycarbonyl)amino)cubane-1-carboxylate (**S4**, 289 mg, 1.04 mmol, 1.00 equiv.) THF (10 mL) and NaOH (416 mg, 10.4 mmol, 10 equiv.). Extraction with CH_2Cl_2, drying over $MgSO_4$ and concentration under reduced pressure afforded 4-((*tert*-butoxycarbonyl)amino)cubane-1-carboxylic acid (**S12**, 230 mg, 0.874 mmol, 84%) as a white solid.

Formula $C_{14}H_{17}NO_4$;
MW 263.29 $g.mol^{-1}$;
TLC R_f n/a;
^{1}H NMR (500 MHz, CD_3OD) δ 4.03 (br s, 6H), 1.45 (s, 9H) ppm;
^{13}C NMR (126 MHz, CD_3OD) δ 176.4 (COOH), 156.7 (NH*C*OO*t*Bu), 80.9 (*C*–NHBoc), 67.9 (O*C*(CH_3)$_3$), 57.9 (*C*–COOH), 51.2 (3C, CH cubyl), 45.9 (3C, CH cubyl), 28.8 (3C, C(CH_3)$_3$) ppm;
IR 2978 (m), 2936 (br), 2859 (m), 1608 (s), 1441 (s), 1290 (s), 1025 (s), 841 (m), 568 (m) cm^{-1};
HRMS (ESI) m/z for $[C_{14}H_{16}NO_4]^-$ $[M–H]^-$ calcd: 262.1085 found: 262.1078.

8.5 Synthesis of 4-(piperidine-1-carbonyl)cubane-1-carboxylic acid (S13)

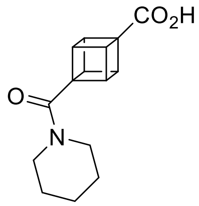

Prepared according to the **general procedure D** using methyl 4-(piperidine-1-carbonyl)cubane-1-carboxylate **S5** (407 mg, 1.49 mmol, 1.00 equiv.) THF (10 mL) and NaOH (600 g, 15.0 mmol, 10.0 equiv.). Extraction with CH_2Cl_2, drying over $MgSO_4$ and concentration under reduced pressure afforded 4-(piperidine-1-carbonyl)cubane-1-carboxylic acid (**S13**, 346 mg, 1.33 mmol, 89%) as a white solid.

Formula $C_{15}H_{17}NO_3$;
MW 259.31 $g.mol^{-1}$;
^{1}H NMR (400 MHz, $CDCl_3$) δ 4.18 – 4.31 (m, 6H) 3.48 – 3.60 (m, 2H) 3.13 – 3.24 (m, 2H) 1.62 – 1.72 (m, 2H) 1.50 – 1.62 (m, 4H) ppm;

^{13}**C NMR** (101 MHz, $CDCl_3$) δ 176.6 (COOH), 169.3 (CON), 58.2 (*C*–CON), 54.5 (*C*–COOH), 47.1 (3C, CH cubyl), 46.5 (3C, CH cubyl), 46.0 (NCH_2), 42.9 (NCH_2), 26.7 (CH_2), 25.4 (CH_2), 24.6 (CH_2) ppm;
IR 2933 (w), 2851 (w), 1704 (s), 1568 (s), 1470 (m), 1290 (m), 1200 (s), 855 (m) cm^{-1};
HRMS (ESI) m/z for $[C_{15}H_{18}NO_3]^+$ $[M+H]^+$ calcd: 260.1281 found: 260.1275.

8.6 Synthesis of 4-(diethylcarbamoyl)cubane-1-carboxylic acid (S14)

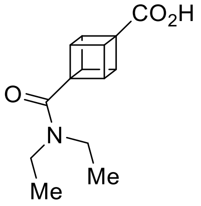

Prepared according to the **general procedure D** using methyl 4-(diethylcarbamoyl)cubane-1-carboxylate (**S6**, 385 mg, 1.47 mmol, 1.00 equiv.) THF (10 mL) and NaOH (600 mg, 15.0 mmol, 10.0 equiv.). Extraction with CH_2Cl_2, drying over $MgSO_4$ and concentration under reduced pressure afforded 4-(diethylcarbamoyl)cubane-1-carboxylic acid (**S14**, 346 mg, 1.40 mmol, 95%) as a white solid.

Formula $C_{14}H_{17}NO_3$;
MW 247.29 g.mol^{-1};
TLC R_f n/a;
^{1}**H NMR** (400 MHz, $CDCl_3$) δ 4.25 (6H, br s), 3.37 (2H, q, *J* = 6.5 Hz), 3.15 (2H, q, *J* = 7.0 Hz), 1.21 (3H, t, *J* = 6.8 Hz), 1.13 (3H, t, *J* = 6.8 Hz) ppm;
^{13}**C NMR** (101 MHz, $CDCl_3$) δ 176.6 (COOH), 170.4 (CON), 58.5 (*C*–$CONEt_2$), 54.7 (*C*–COOH), 47.3 (3C, CH cubyl), 46.4 (3C, CH cubyl), 40.9 (NCH_2CH_3), 39.3 (N$\mathit{CH_2}CH_3$), 14.6 ($NCH_2\mathit{CH_3}$), 12.8 ($NCH_2\mathit{CH_3}$) ppm;
IR 2987 (br), 2944 (w), 1714 (s), 1591 (s), 1196 (m), 1080 (m), 846 (m), 700 (s), 660 (w) cm^{-1};
HRMS (ESI) *m/z* 246.9 for $[C_{15}H_{16}NO_3]^-$ $[M–H]^-$ calcd: 246.1136 found: 246.1131

8.7 Synthesis of 4-(diisopropylcarbamoyl)cubane-1-carboxylic acid (S15)

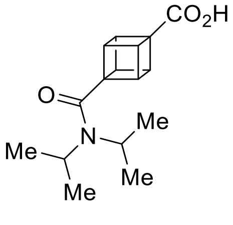

Prepared according to the **general procedure D** using methyl 4-(diisopropylcarbamoyl)cubane-1-carboxylate (**S7**, 340 mg, 1.17 mmol, 1.00 equiv.), THF (10 mL) and powdered NaOH (1.44 g, 36.0 mmol, 30.0 equiv.). Extraction with CH_2Cl_2, drying over $MgSO_4$ and concentration under reduced pressure afforded 4-(diisopropylcarbamoyl)cubane-1-carboxylic acid (**S15**, 320 mg, 1.16 mmol, 97%) as a white solid.

Formula $C_{16}H_{21}NO_3$;

MW 275.35 g.mol^{-1};

TLC R_f n/a;

^{1}H NMR (400 MHz, $CDCl_3$) δ 4.15 – 4.27 (6H, m), 3.48 (1H, spt, J = 6.7 Hz), 3.32 (1H, spt, J = 6.7 Hz), 1.43 (6H, d, J = 6.7 Hz), 1.22 (6H, d, J = 6.7 Hz) ppm;

^{13}C NMR (101 MHz, $CDCl_3$) δ 176.7 (COOH), 170.2 (CON), 59.4 (*C*–CON(*i*Pr)$_2$), 54.6 (*C*–COOH), 48.5 (*CH*(CH_3)$_2$), 47.0 (3C, CH cubyl), 46.3 (3C, CH cubyl), 46.0 (*CH*(CH_3)$_2$), 21.0 (2C, CH(*CH$_3$*)$_2$), 20.5 (2C, CH(*CH$_3$*)$_2$) ppm;

IR 2971 (br), 2931 (w), 1712 (s), 1578 (s), 1445 (m), 1213 (s), 1036 (m), 729 (s) cm^{-1};

HRMS (ESI) m/z for $[C_{16}H_{22}NO_3]^+$ $[M+H]^+$ calcd: 276.1594 found: 276.1593.

8.8 Synthesis of 4-(morpholine-4-carbonyl)cubane-1-carboxylic acid (S16)

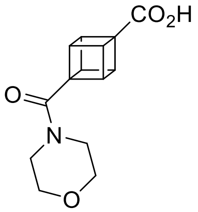

Prepared according to the **general procedure D** using methyl 4-(morpholine-4-carbonyl)-1-cubanecarboxylate (S8, 350 mg, 1.27 mmol, 1.00 equiv.), THF (12 mL) and NaOH (520 mg, 13.0 mmol, 10.0 equiv.). Extraction with EtOAc, drying over $MgSO_4$ and concentration under reduced pressure afforded 4-(morpholine-4-carbonyl)cubane-1-carboxylic acid (**S16**, 230 mg, 0.880 mmol, 69%) as a white solid.

Formula $C_{14}H_{15}NO_4$

MW 261.28 g.mol^{-1}

TLC R_f n/a

^{1}H NMR (400 MHz, $CDCl_3$) δ 4.16 – 4.34 (m, 6H), 3.66 – 3.73 (m, 4H), 3.55 – 3.65 (m, 2H), 3.19-3.31 ppm (m, 2H) ppm;

^{13}C NMR (101 MHz, $CDCl_3$) δ 175.9 (COOH), 169.5 (CON) 66.9 (OCH_2), 66.8 (OCH_2), 57.9 (*C*–CON, 54.4 (*C*–COOH), 47.0 (3C, CH cubyl), 46.5 (3C, CH cubyl), 45.4 (NCH_2), 42.0 (NCH_2) ppm;

IR 3144 (w), 2995 (m), 2918 (m), 1712 (s), 1626 (s), 1428 (s), 1104 (m), 846 (m) cm^{-1}.

HRMS (ESI) m/z for $[C_{14}H_{14}NO_4]^-$ $[M–H]^-$ calcd: 260.0928 found 260.0922.

9 NMR Spectra

9.1 Methyl 4-methoxy-1-cubanecarboxylate (7)

9.1.1 ^{1}H NMR (400 MHz, $CDCl_3$)

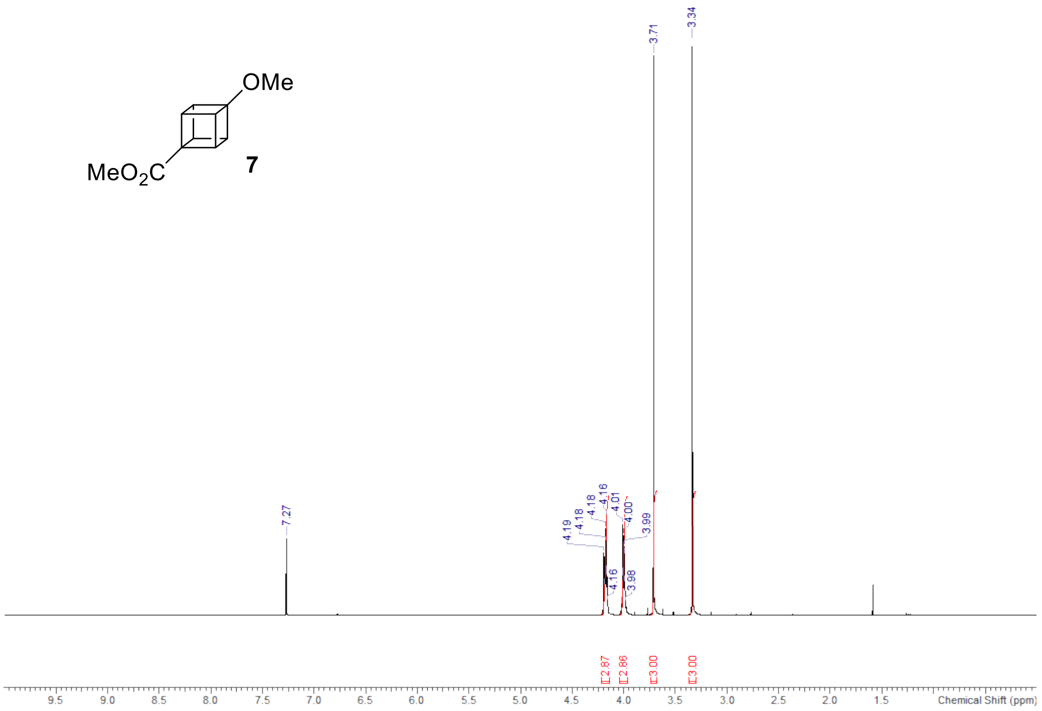

9.1.2 ^{13}C NMR (101 MHz, $CDCl_3$)

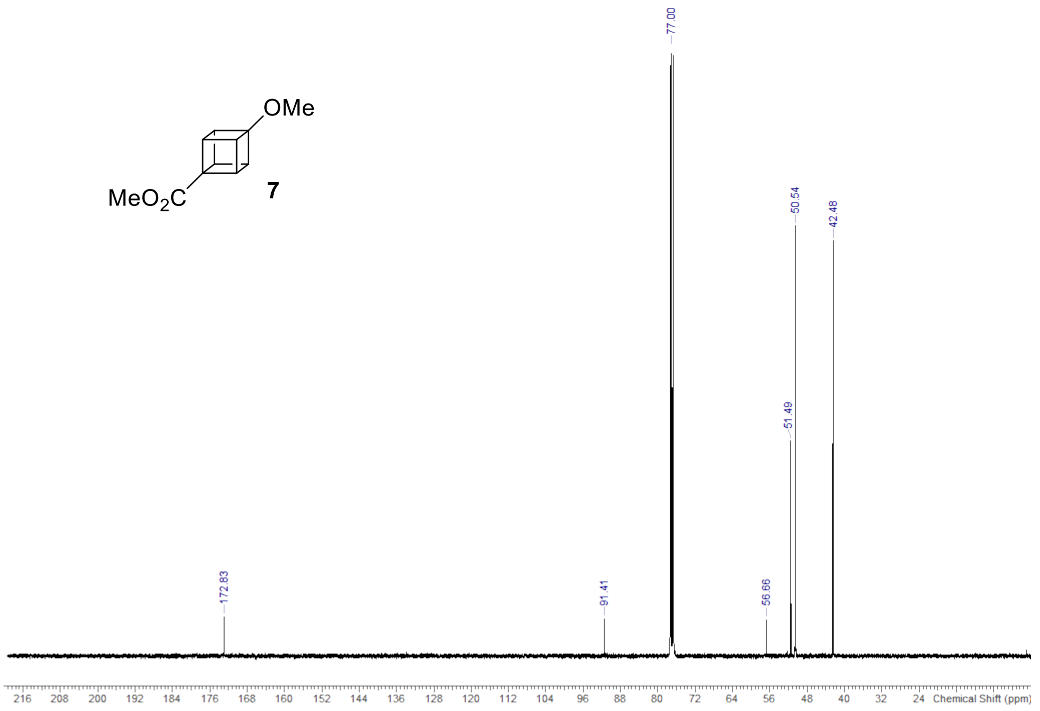

9.2 Methyl cubanecarboxylate (10)

9.2.1 ^{1}H NMR (400 MHz, $CDCl_3$)

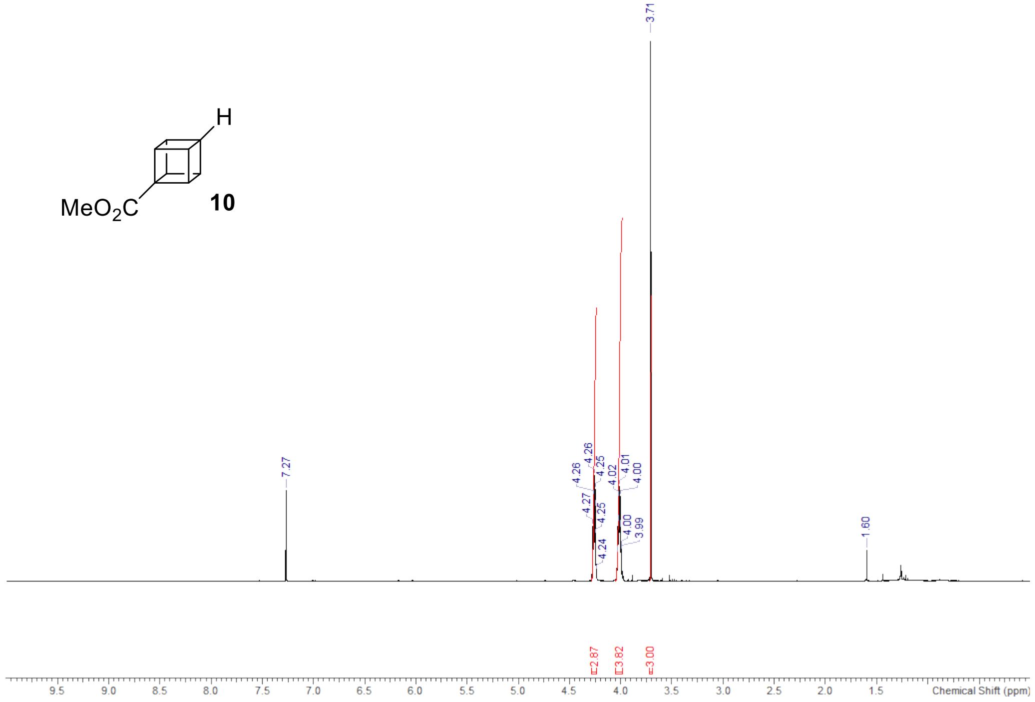

9.2.2 ^{13}C NMR (101 MHz, $CDCl_3$)

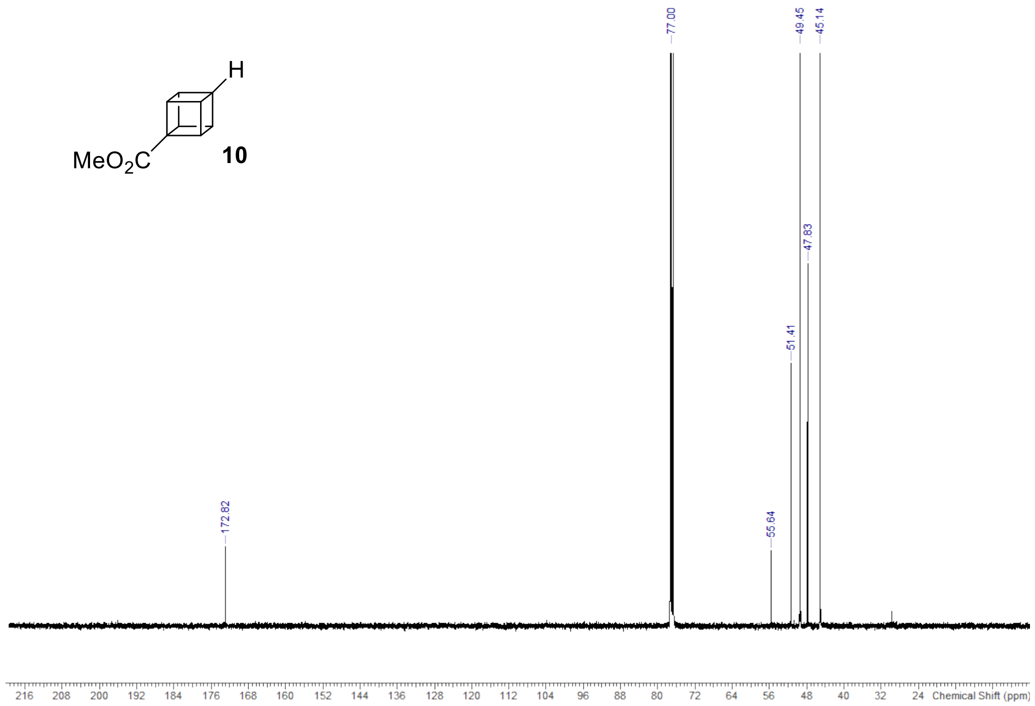

9.3 Methyl 4-methoxy(d_3)-1-cubanecarboxylate (11)

9.3.1 ^{1}H NMR (400 MHz, $CDCl_3$)

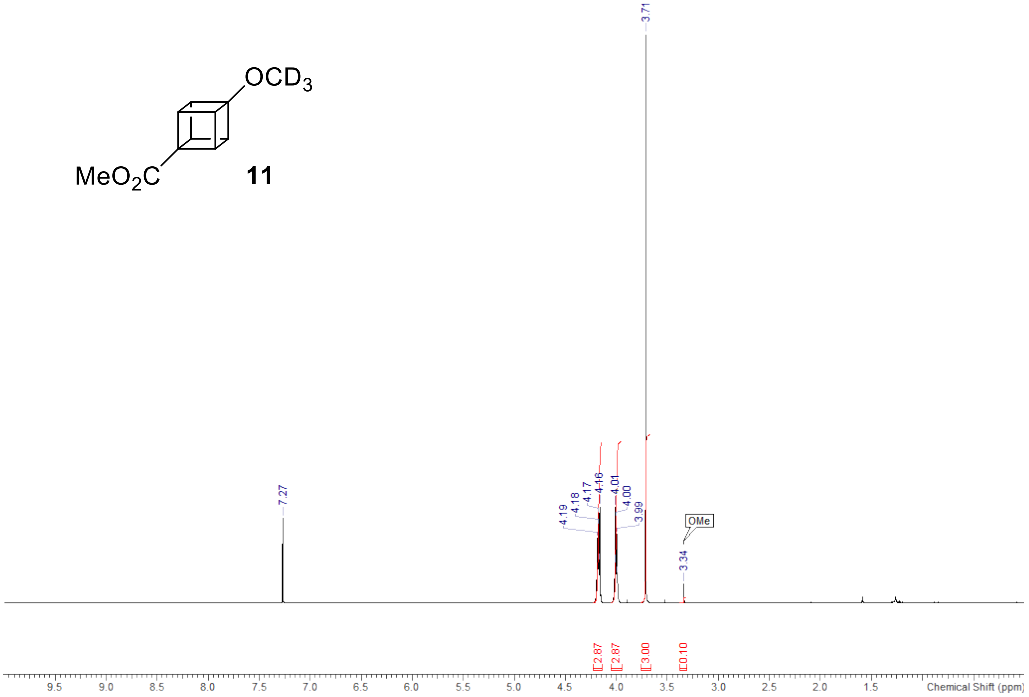

9.3.2 ^{13}C NMR (101 MHz, $CDCl_3$)

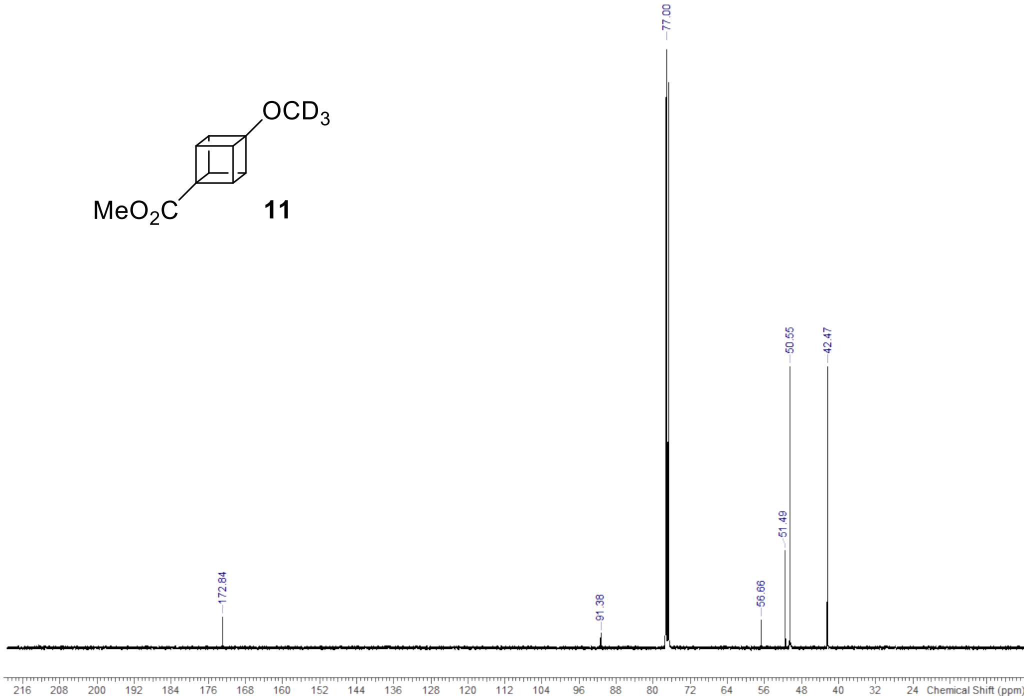

9.4 Methyl 4-ethoxy-1-cubanecarboxylate (12)

9.4.1 ^{1}H NMR (400 MHz, $CDCl_3$)

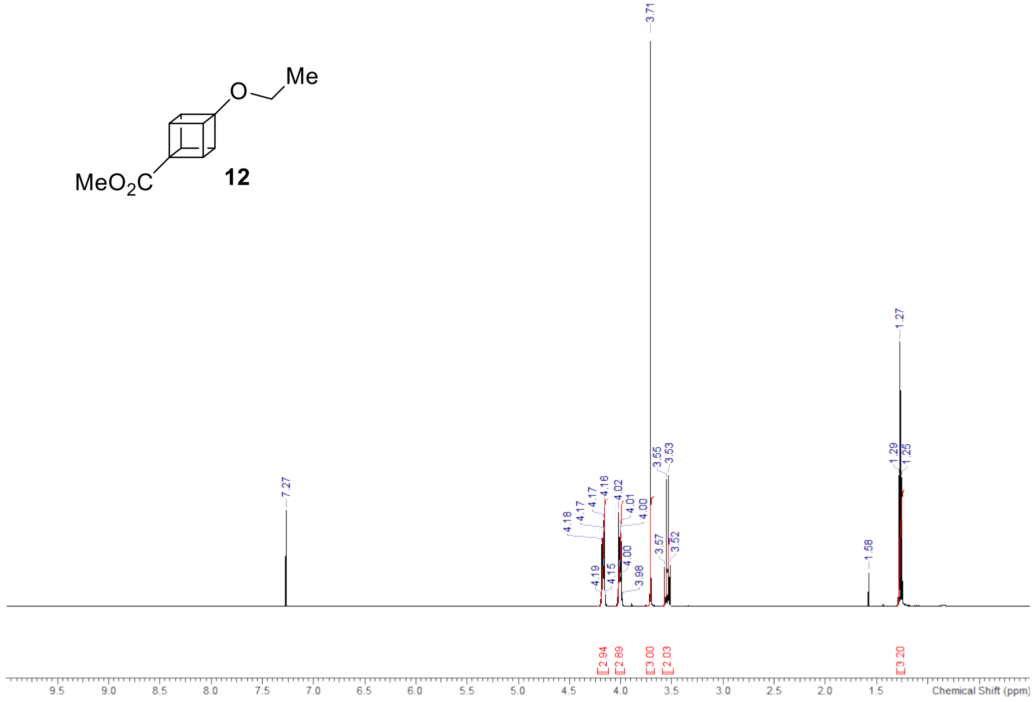

9.4.2 ^{13}C NMR (101 MHz, $CDCl_3$)

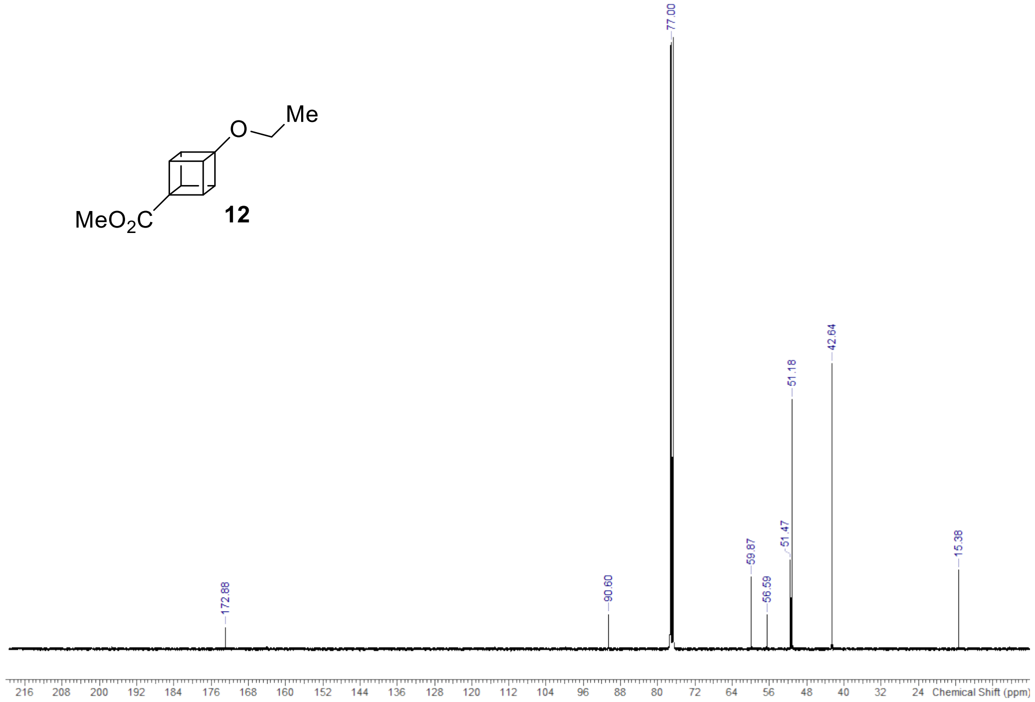

9.5 Methyl 4-isopropoxy-1-cubanecarboxylate (13)

9.5.1 ^{1}H NMR (400 MHz, $CDCl_3$)

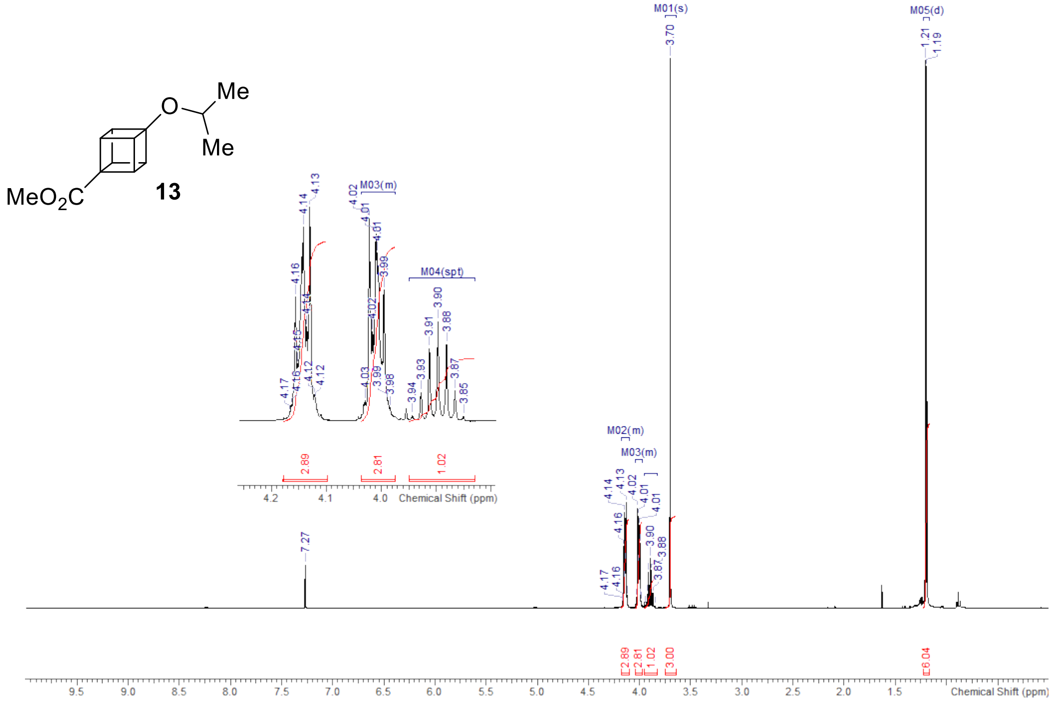

9.5.2 ^{13}C NMR (101 MHz, $CDCl_3$)

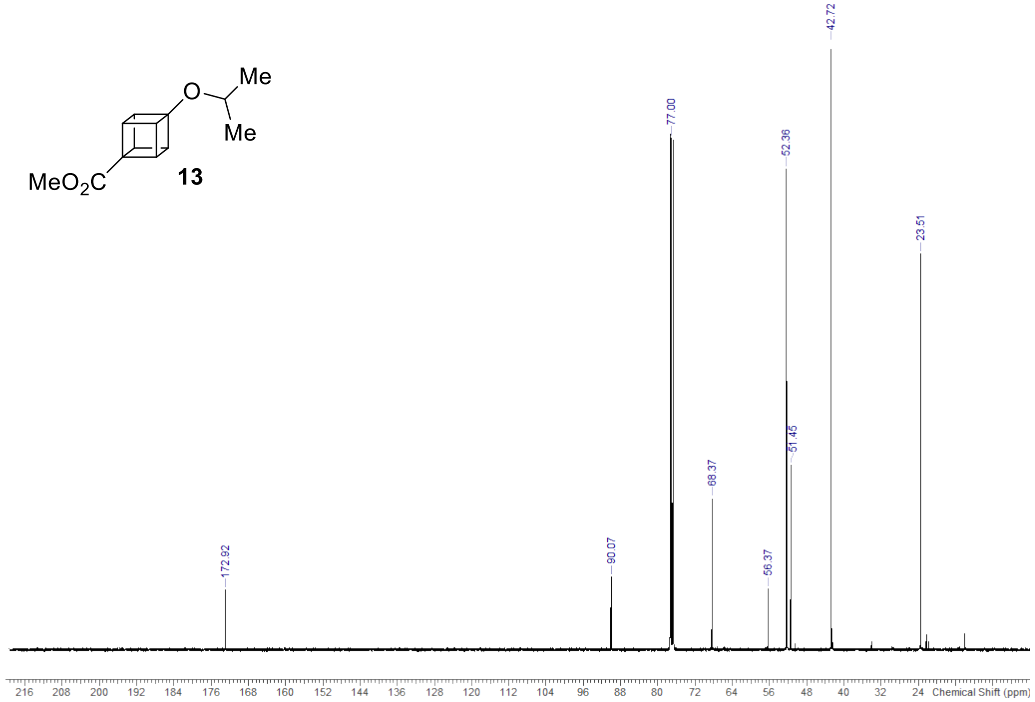

9.6 Methyl 4-trifluoroethoxy-1-cubanecarboxylate (14)

9.6.1 ^{1}H NMR (400 MHz, $CDCl_3$)

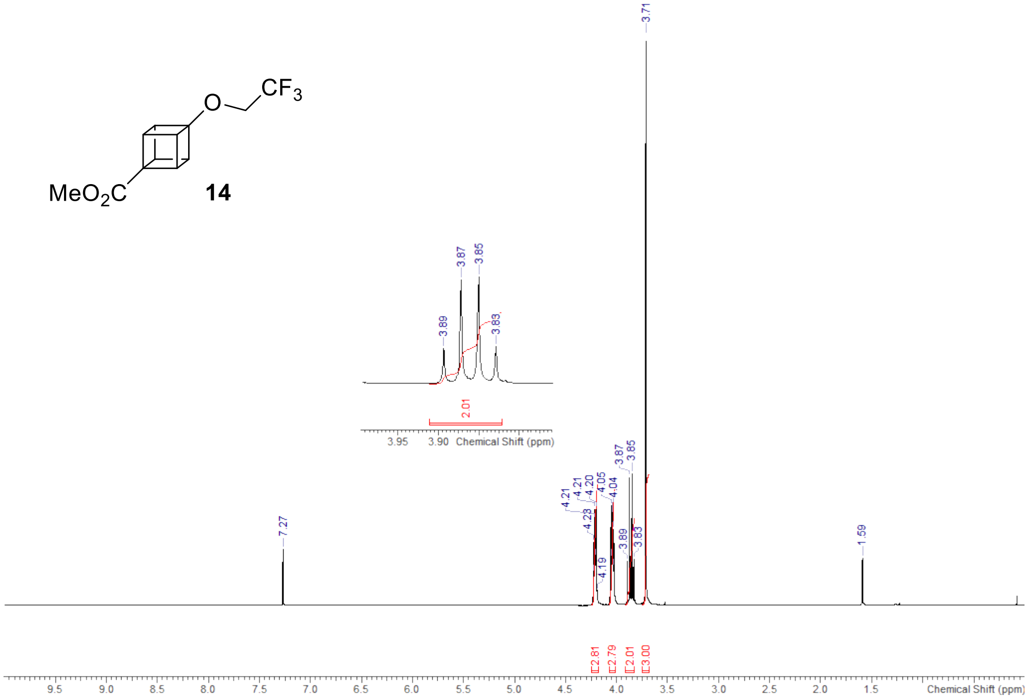

9.6.2 ^{13}C NMR (101 MHz, $CDCl_3$)

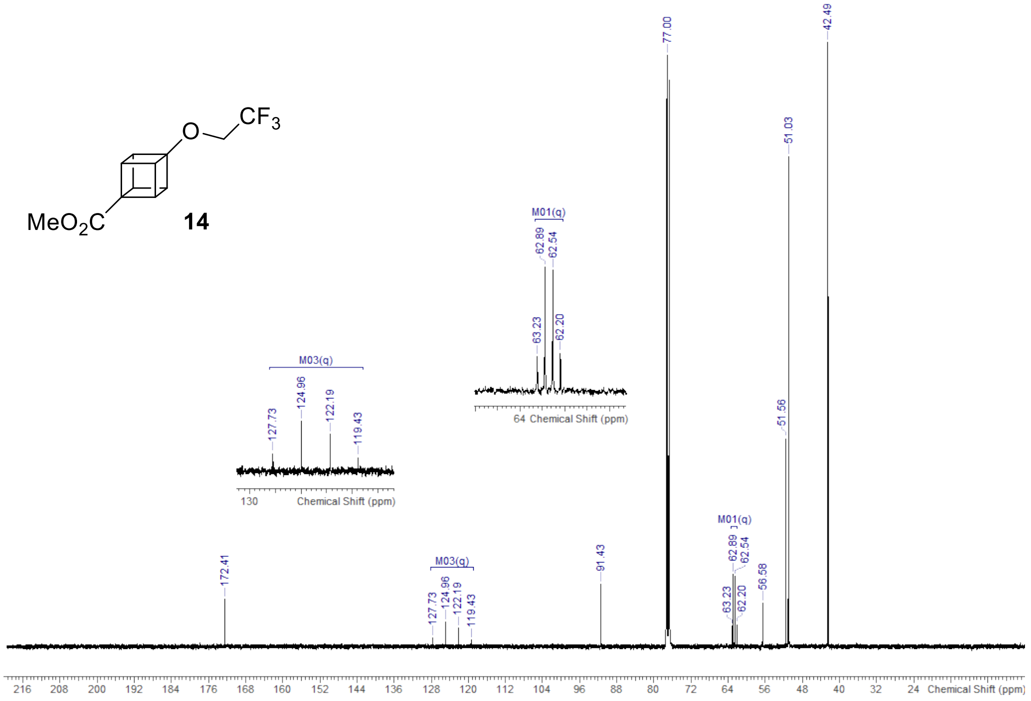

9.6.3 ^{19}F NMR (376 MHz, $CDCl_3$)

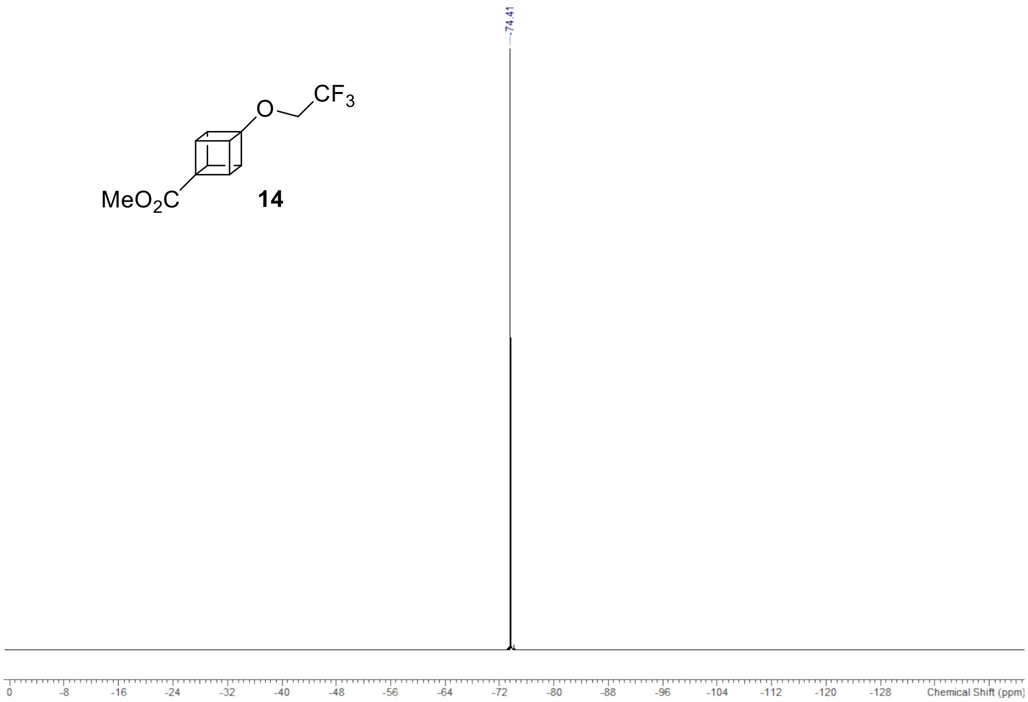

9.7 Methyl 4-(1,1,1,3,3,3-hexafluoroisopropoxy)-1-cubanecarboxylate (15)

9.7.1 ^{1}H NMR (400 MHz, $CDCl_3$)

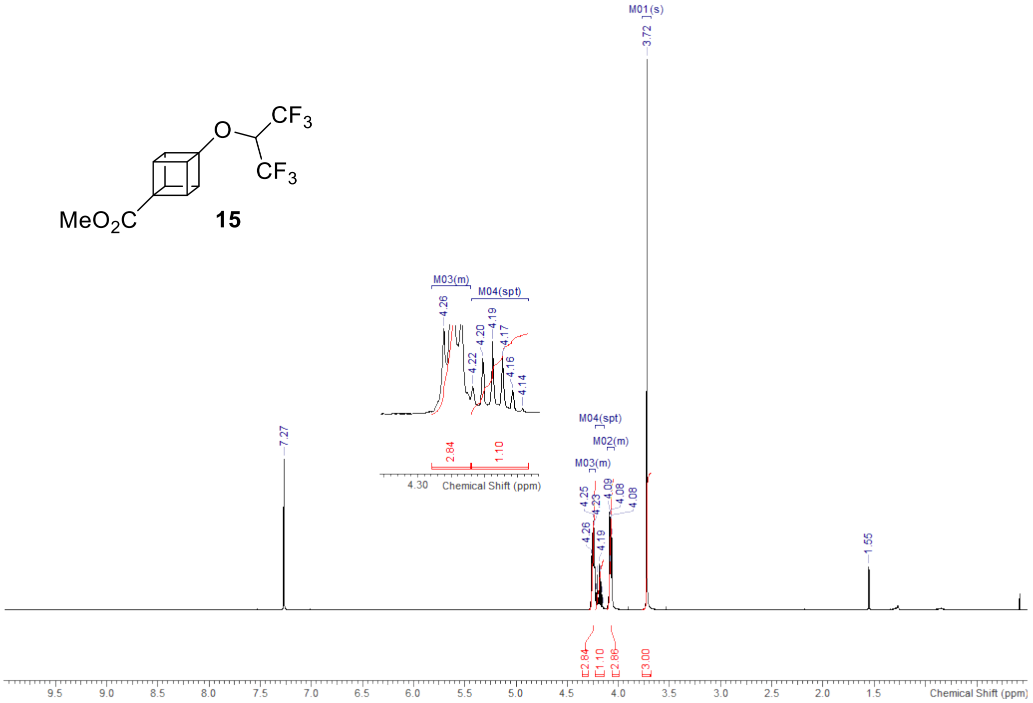

9.7.2 ^{13}C NMR (126 MHz, $CDCl_3$)

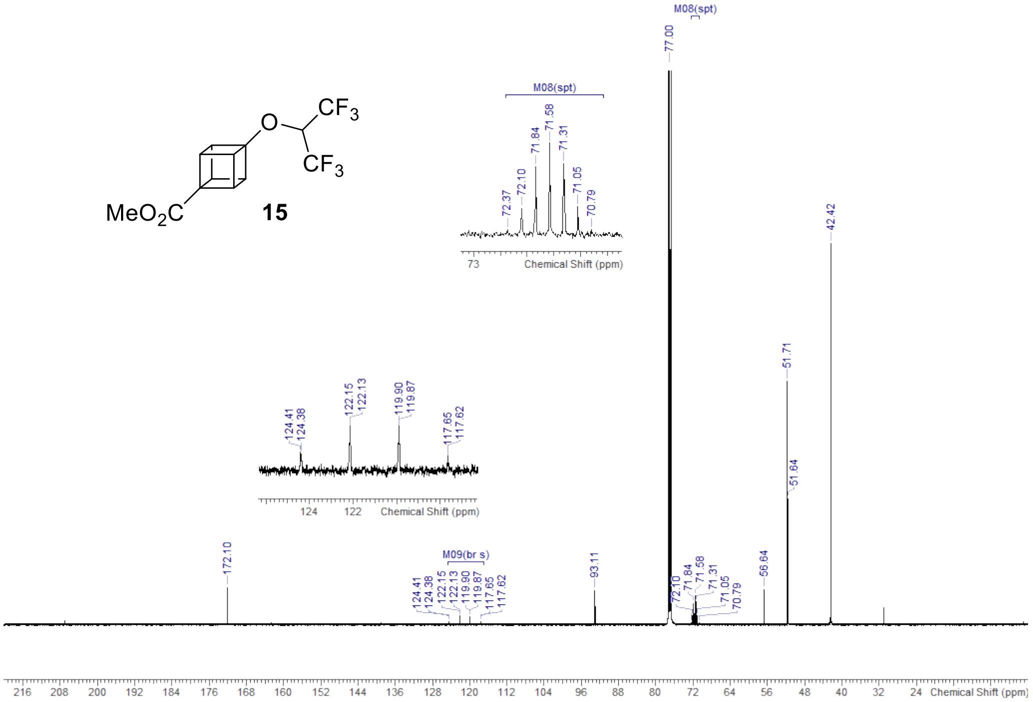

9.7.3 ^{19}F NMR (376 MHz, CDl_3)

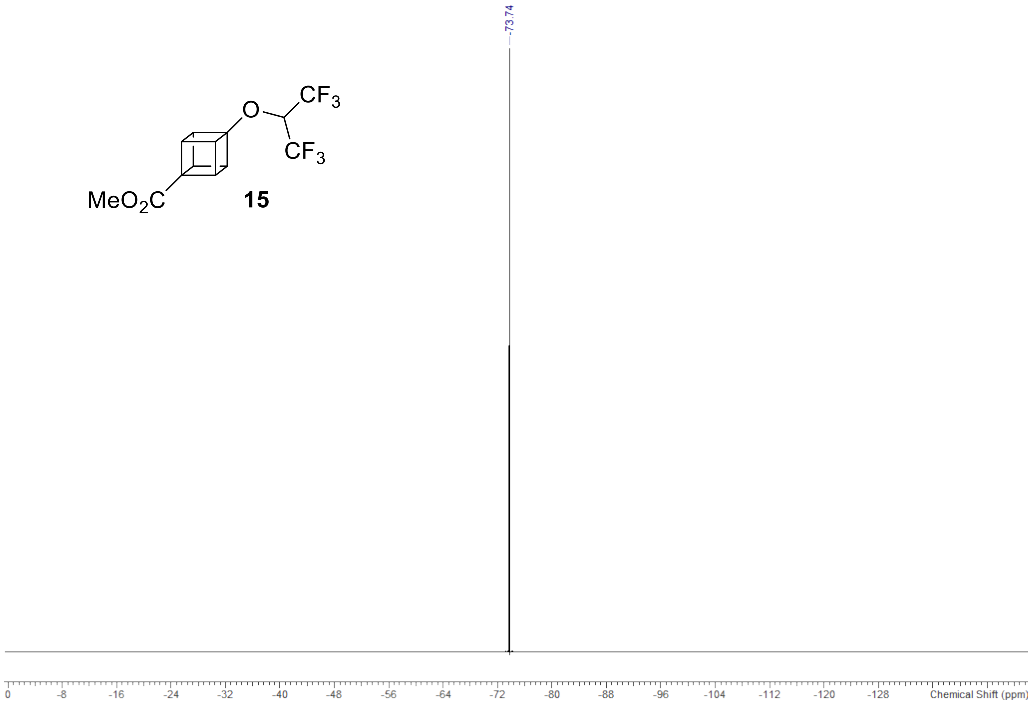

9.7.4 (4-Methoxycubyl)(piperidin-1-yl)methanone (16)

9.7.5 ^{1}H NMR (400 MHz, $CDCl_3$)

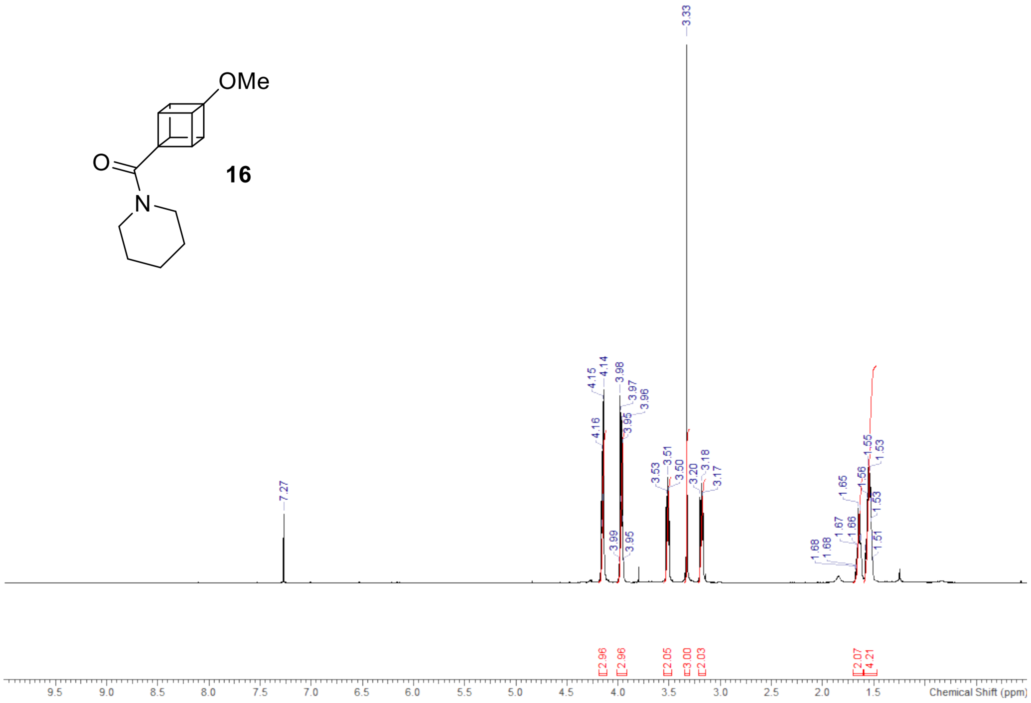

9.7.6 ^{13}C NMR (101 MHz, $CDCl_3$)

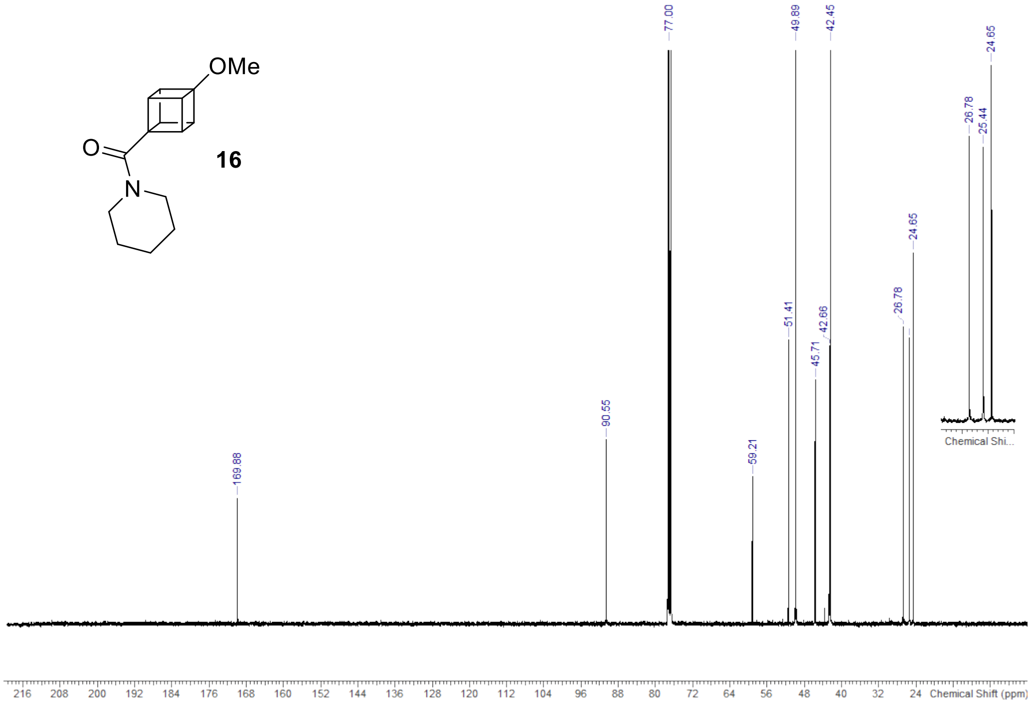

9.8 *N*,*N*-Diethyl-4-methoxycubane-1-carboxamide (17)

9.8.1 ^{1}H NMR (400 MHz, $CDCl_3$)

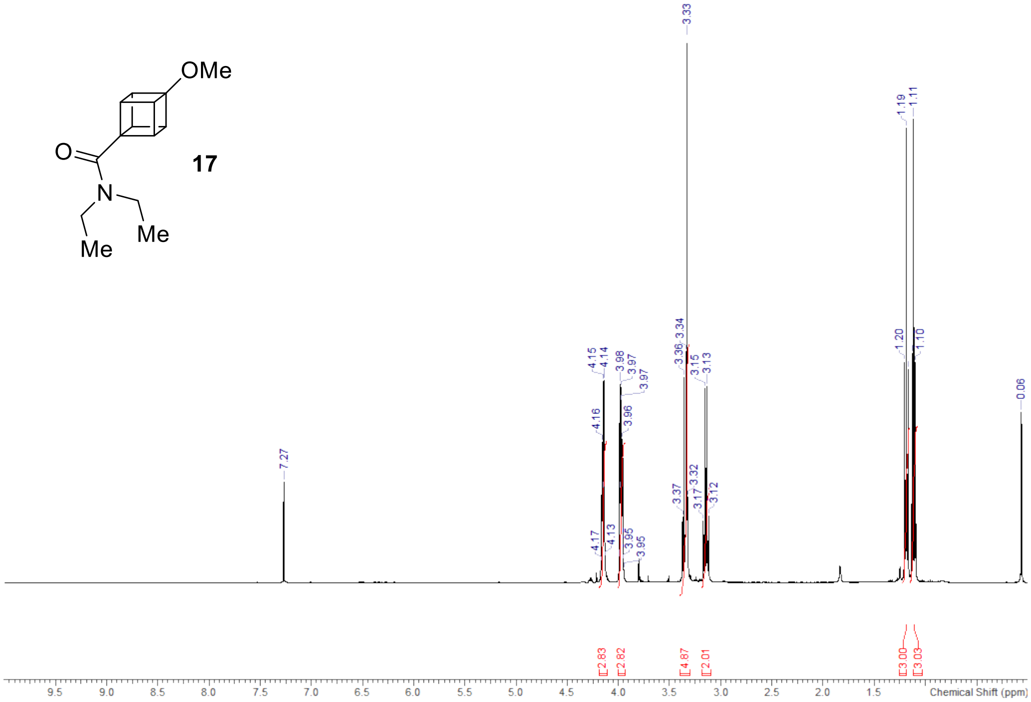

9.8.2 ^{13}C NMR (101 MHz, $CDCl_3$)

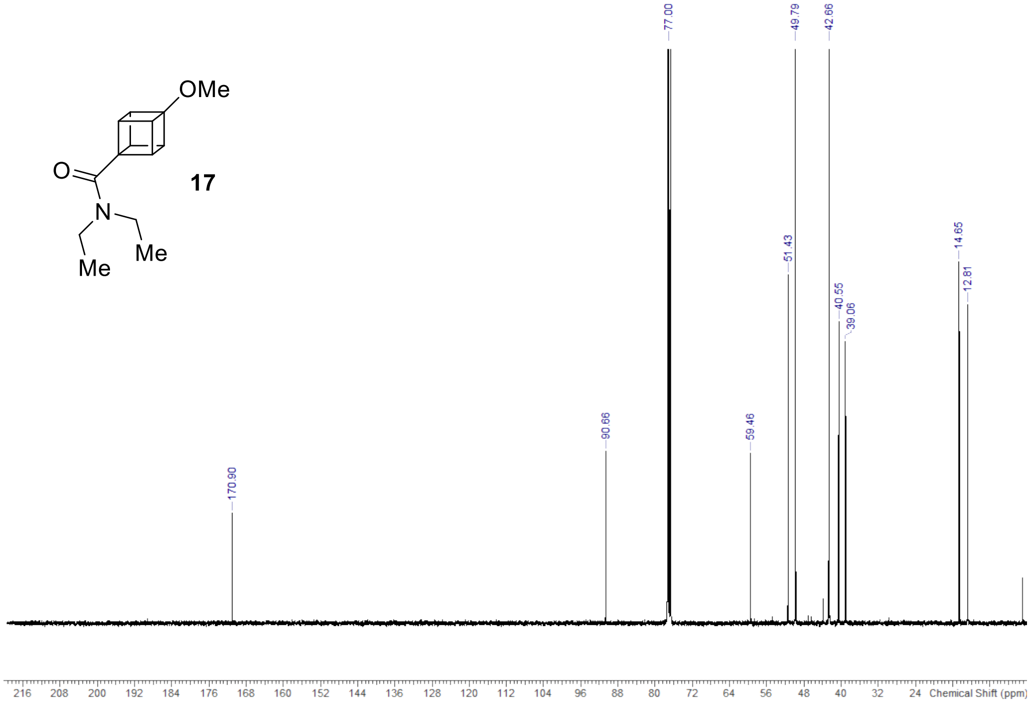

9.8.3 *N*,*N*-Diisopropyl-4-methoxycubane-1-carboxamide (18)

9.8.4 ^{1}H NMR (400 MHz, $CDCl_3$)

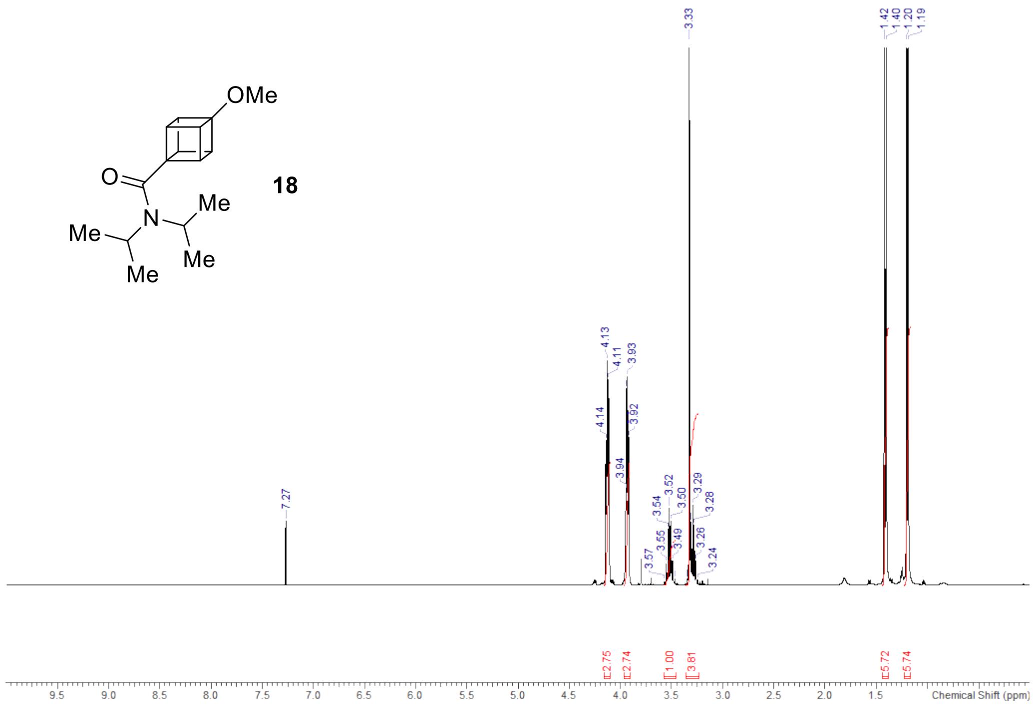

9.8.5 ^{13}C NMR (101 MHz, $CDCl_3$)

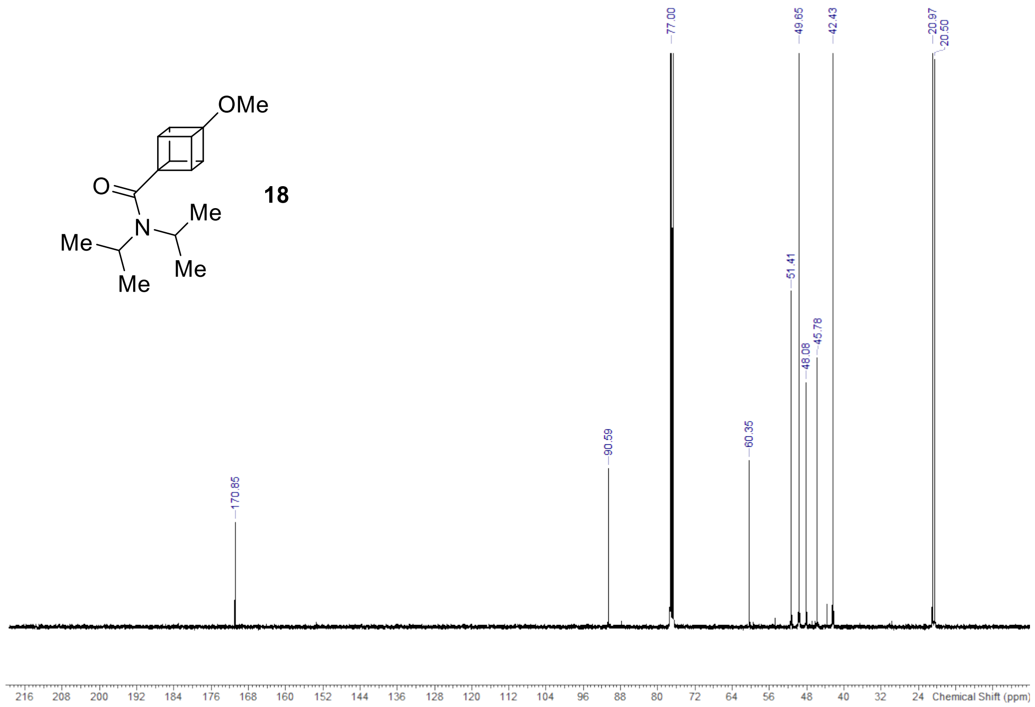

9.9 (4-Methoxycubyl)(morpholino)methanone (19)

9.9.1 ^{1}H NMR (400 MHz, $CDCl_3$)

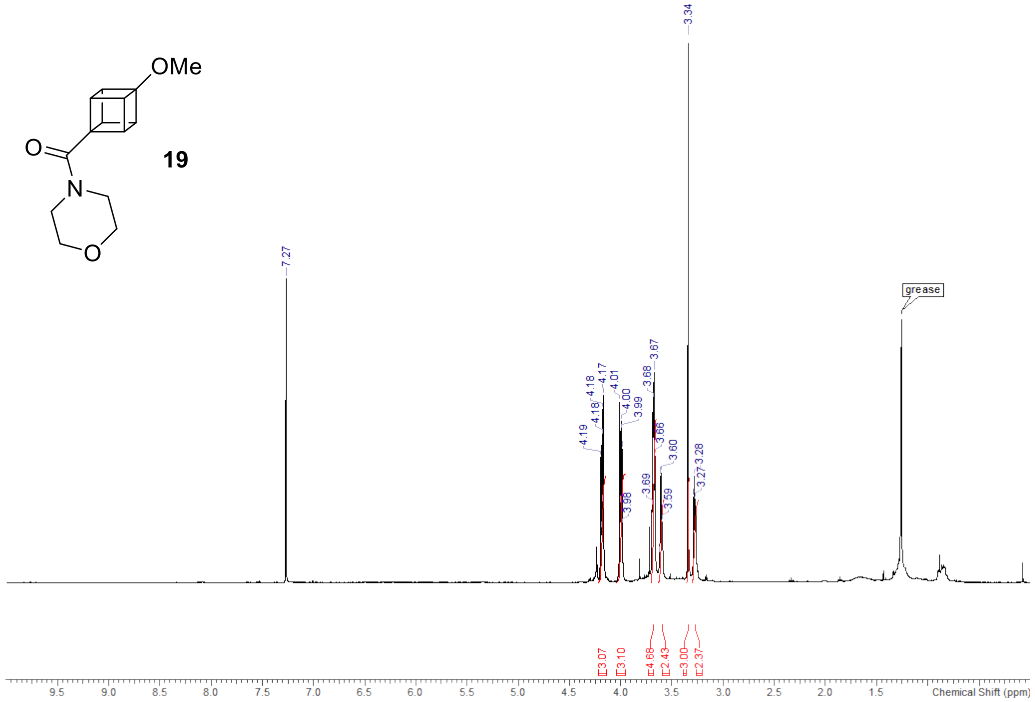

9.9.2 ^{13}C NMR (101 MHz, $CDCl_3$)

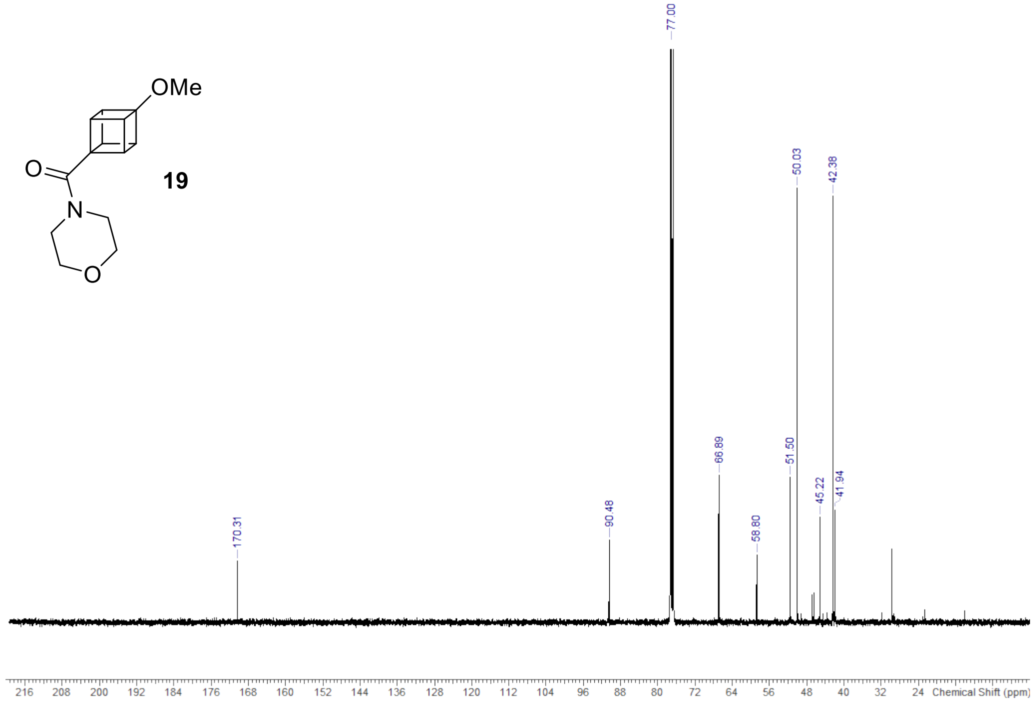

9.10 4-Methoxy-1-cubanebenzylether (20)

9.10.1 ^{1}H NMR (400 MHz, $CDCl_3$)

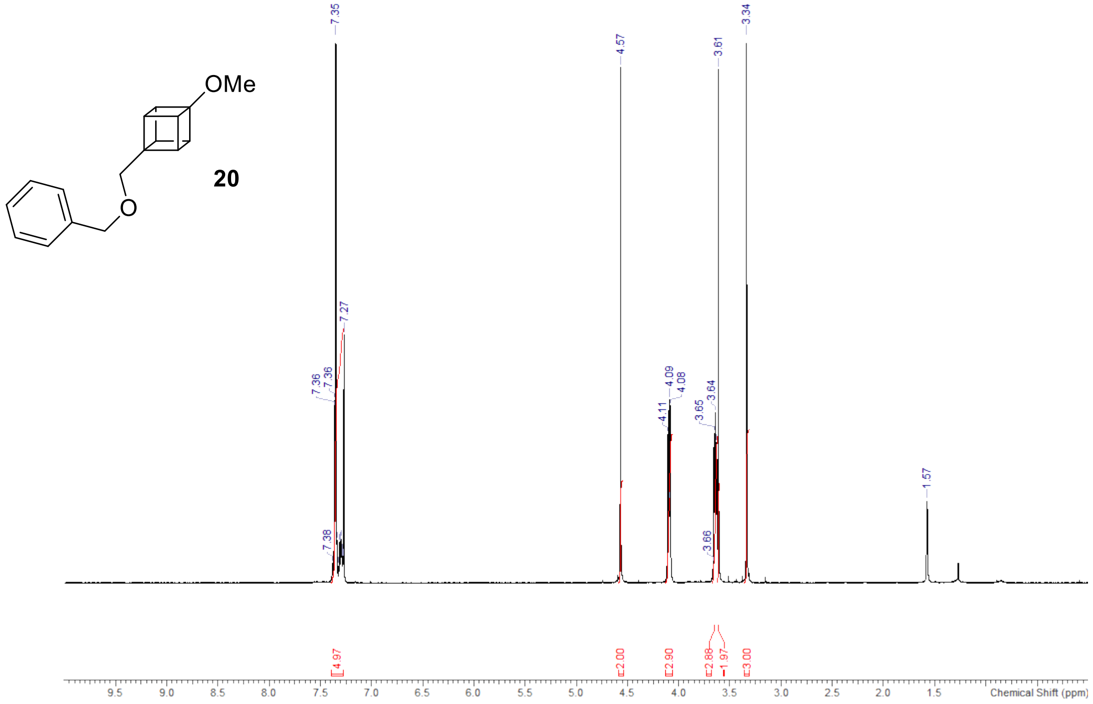

9.10.2 ^{13}C NMR (101 MHz, $CDCl_3$)

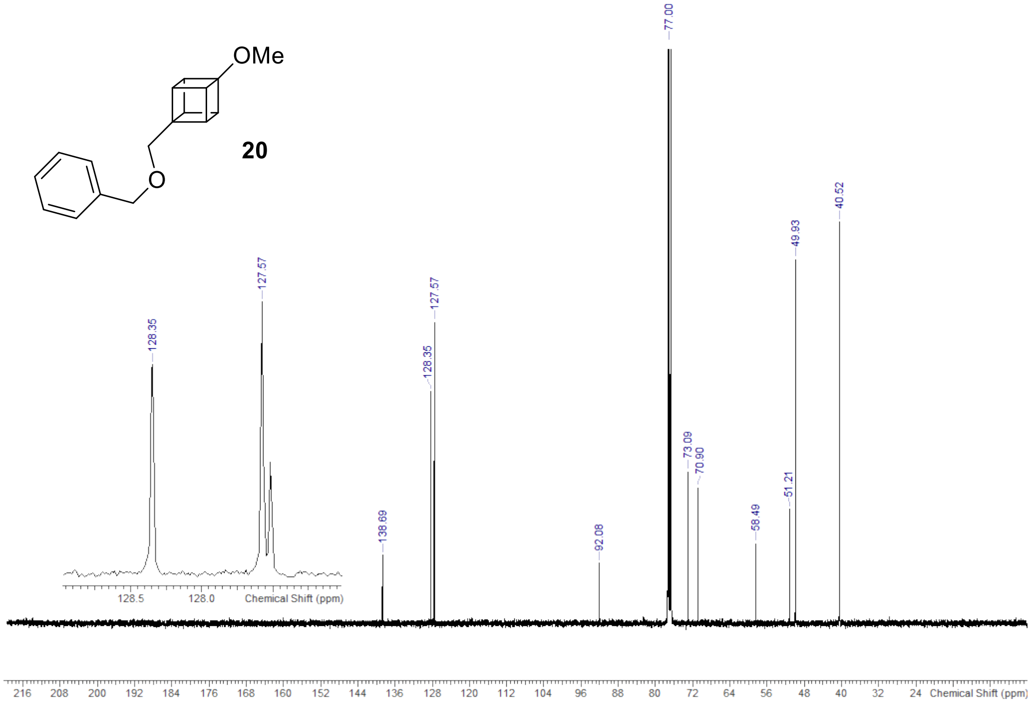

9.11 4-Methoxy-1-cubylmethanol (21)

9.11.1 ^{1}H NMR (400 MHz, $CDCl_3$)

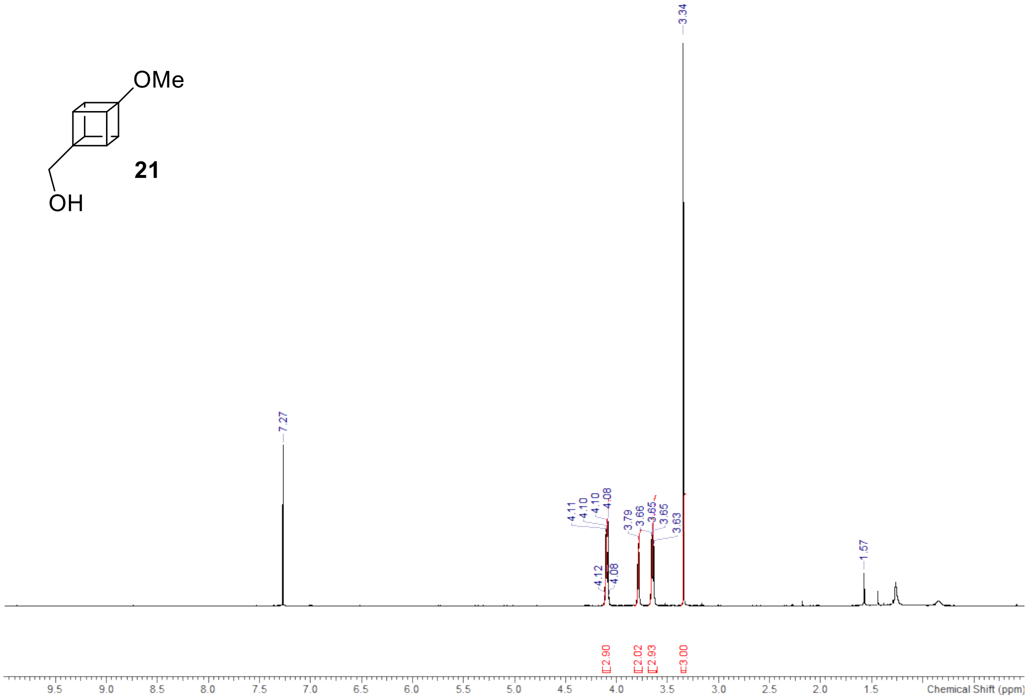

9.11.2 ^{13}C NMR (101 MHz, $CDCl_3$)

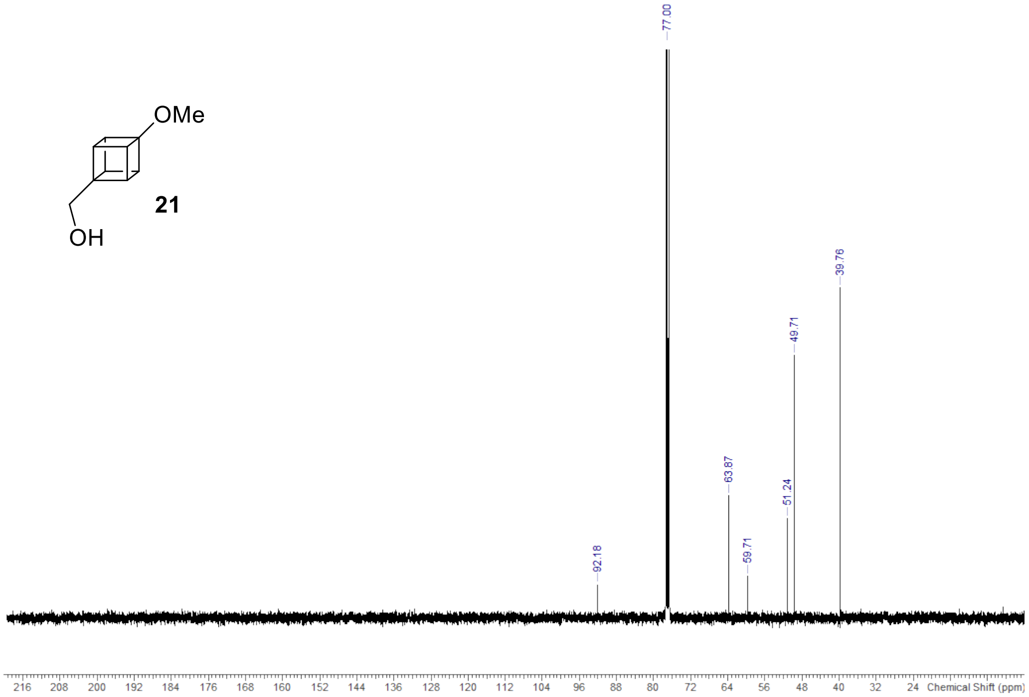

9.12 *tert*-Butyl 4-methoxy-1-cubanecarboxylate (22)

9.12.1 ^{1}H NMR (400 MHz, CDCl$_3$)

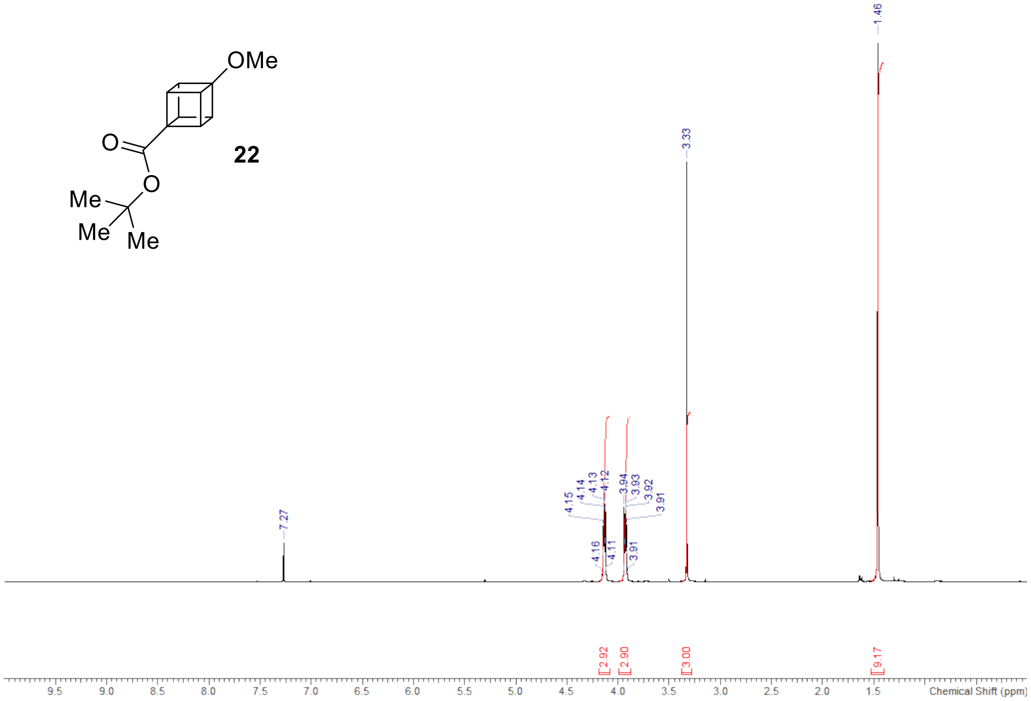

9.12.2 ^{13}C NMR (101 MHz, CDCl$_3$)

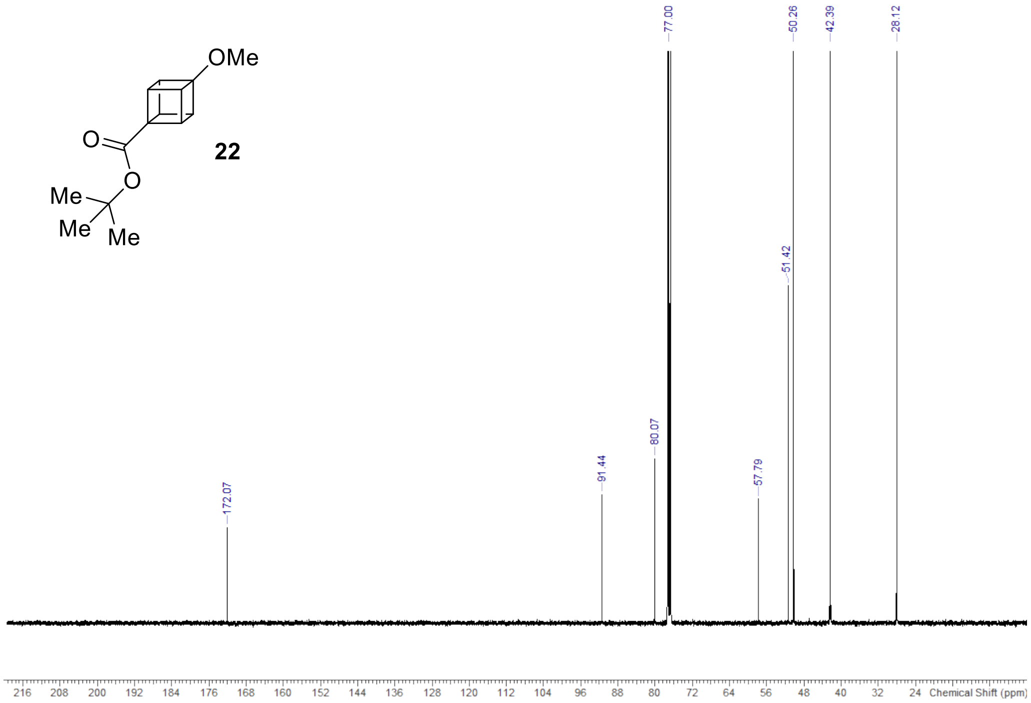

9.13 *tert*-Butyl 4-methoxy-1-cubylcarbamate (23) (from 24)

9.13.1 ^{1}H NMR (400 MHz, $CDCl_3$)

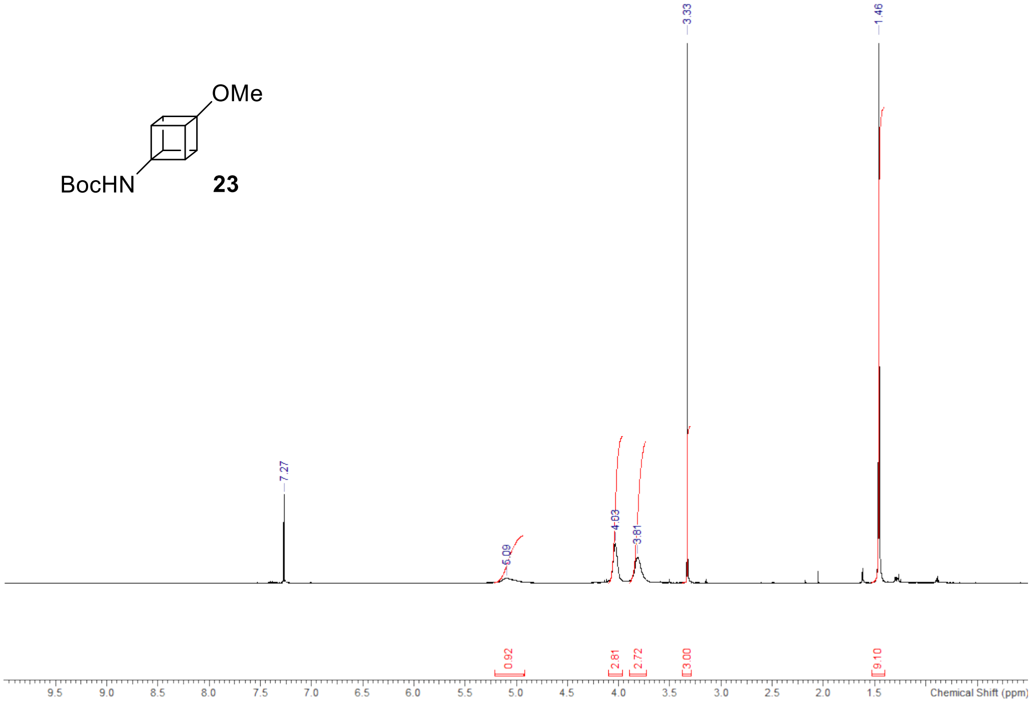

9.13.2 ^{13}C NMR (126 MHz, $CDCl_3$)

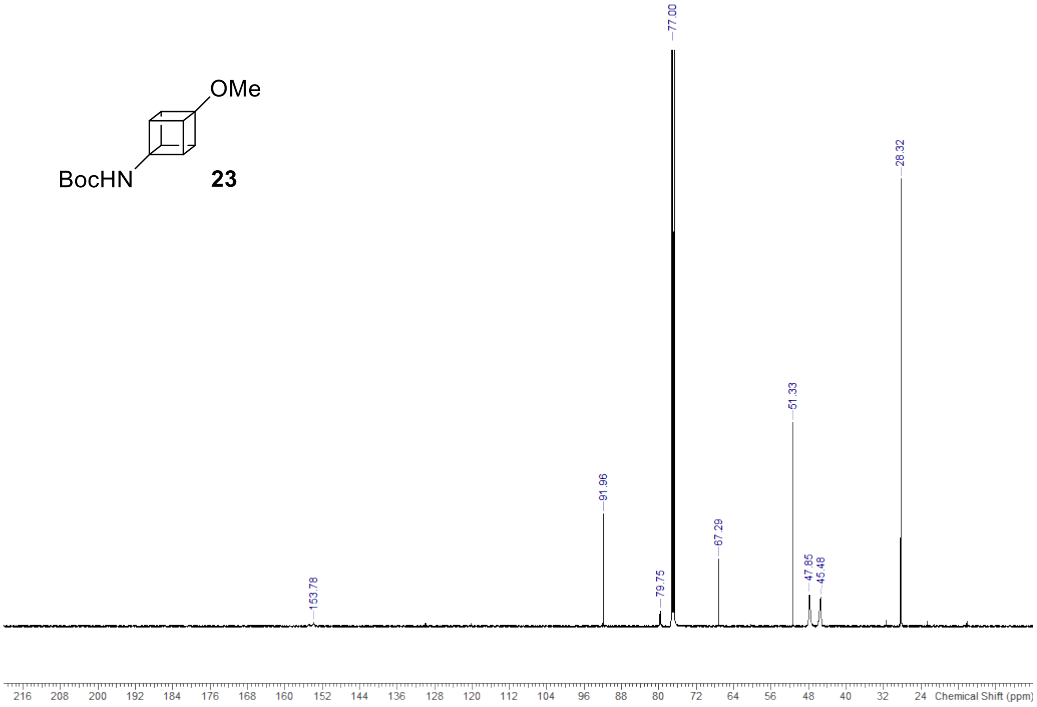

9.14 4-Methoxy-1-cubanecarboxylic acid (24)

9.14.1 ^{1}H NMR (400 MHz, $CDCl_3$)

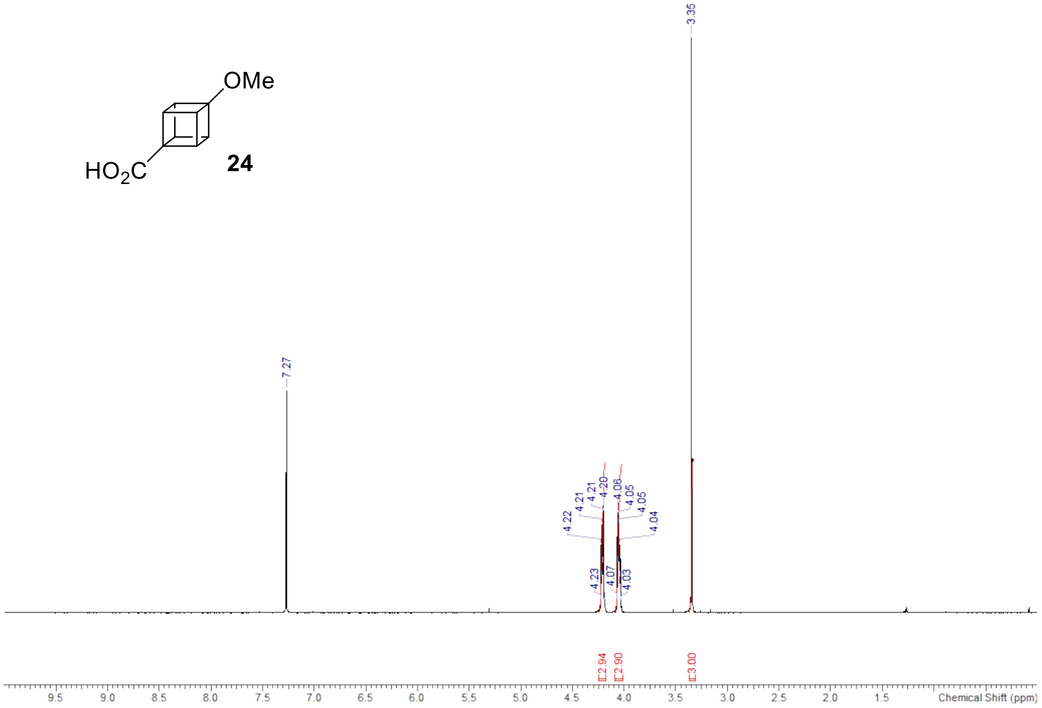

9.14.2 ^{13}C NMR (101 MHz, $CDCl_3$)

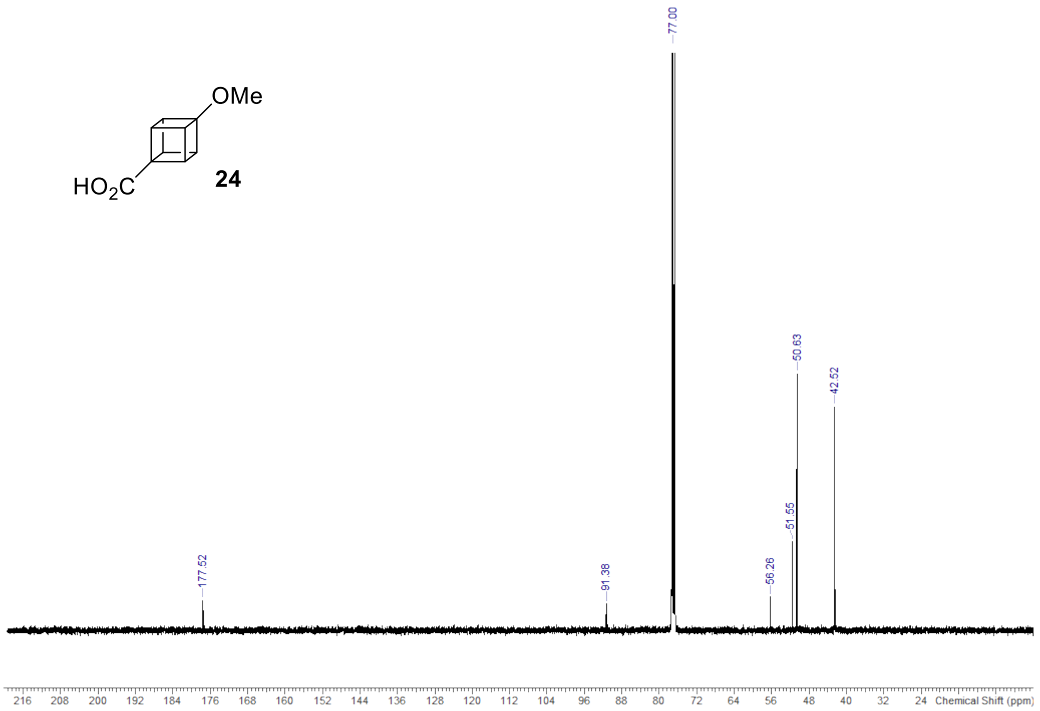

9.15 4-Methoxy-1-cubanamine hydrochloride (25)

9.15.1 ^{1}H NMR (400 MHz, D_2O)

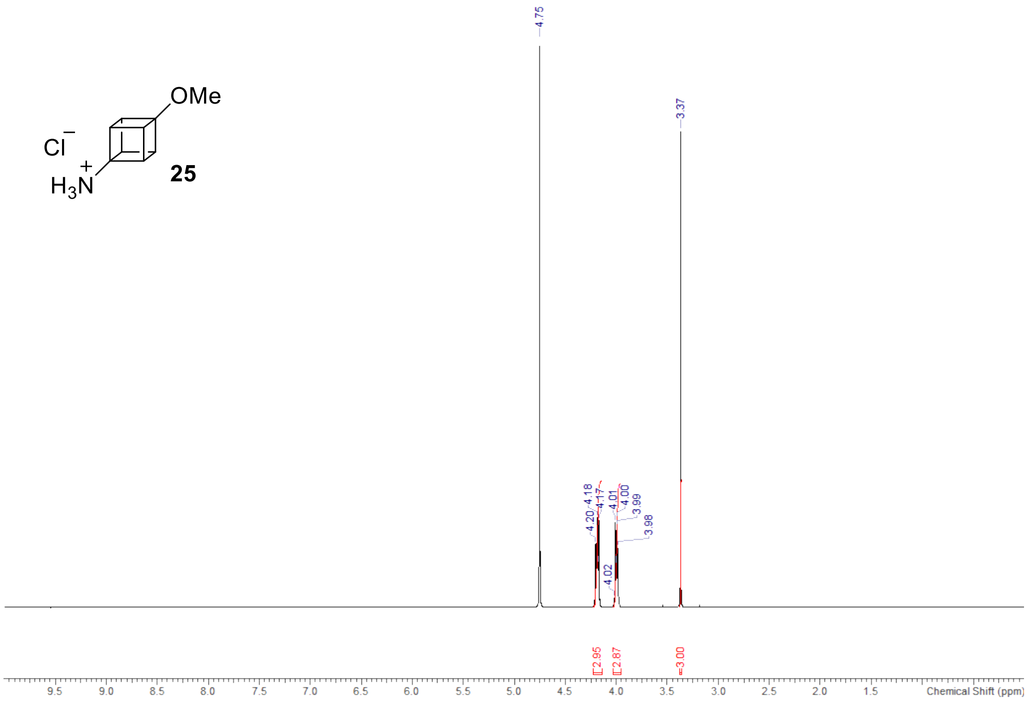

9.15.2 ^{13}C NMR (101 MHz, D_2O)

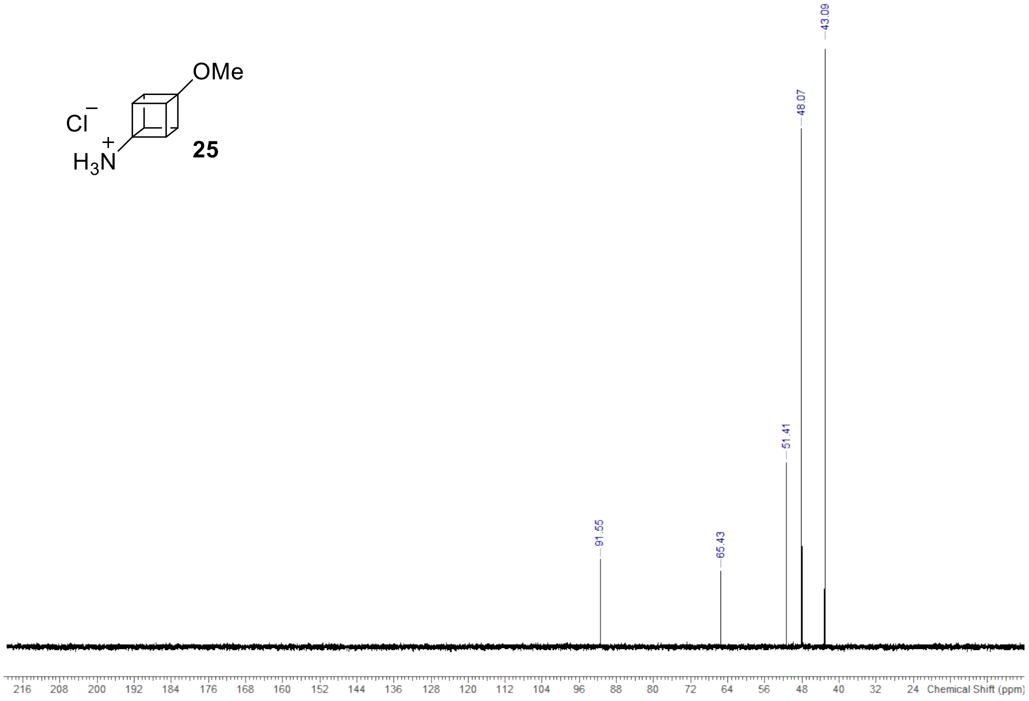

9.16 4-Methoxy-1-cubancarbaldehyde (27)

9.16.1 ^{1}H NMR (400 MHz, $CDCl_3$)

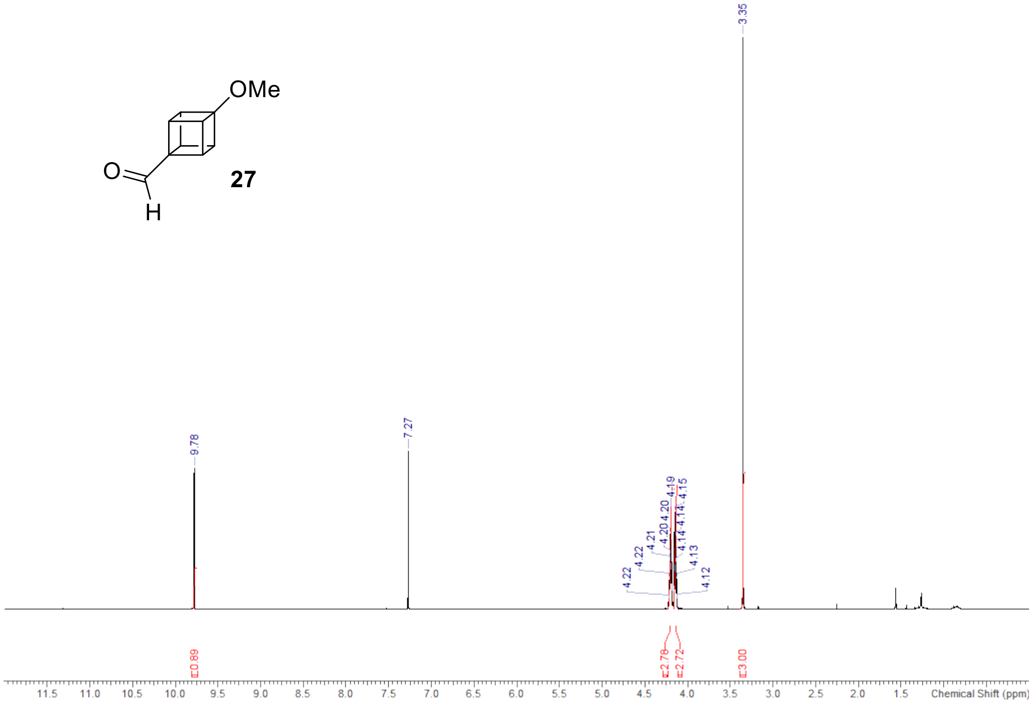

9.16.2 ^{13}C NMR (101 MHz, $CDCl_3$)

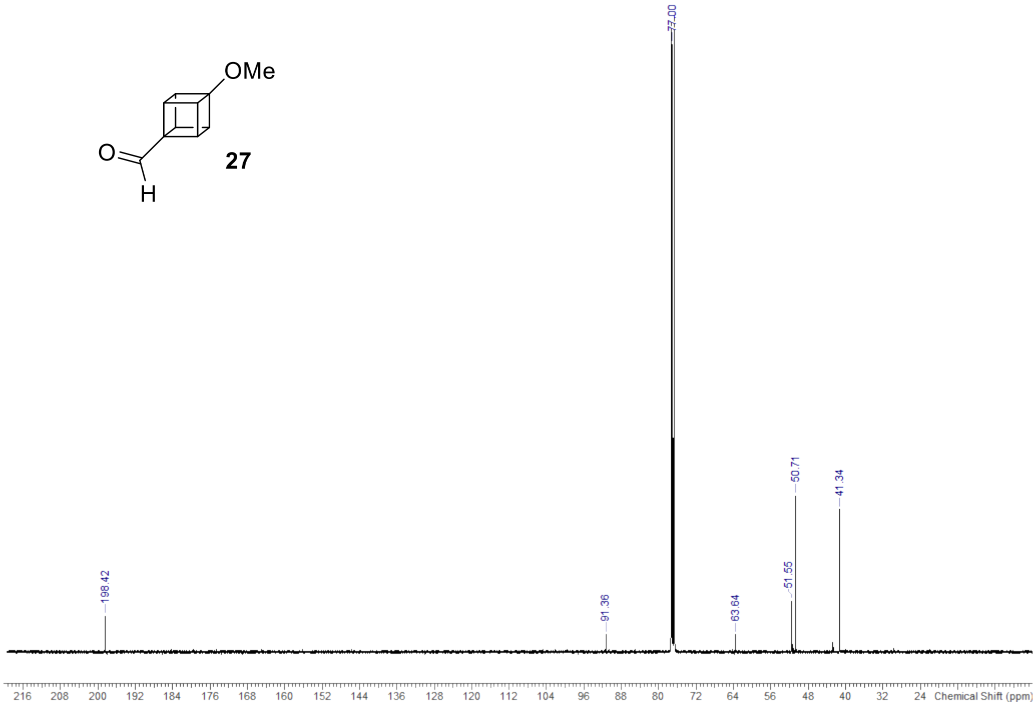

9.17 Cubanisindione (29)

9.17.1 ^{1}H NMR (400 MHz, $CDCl_3$)

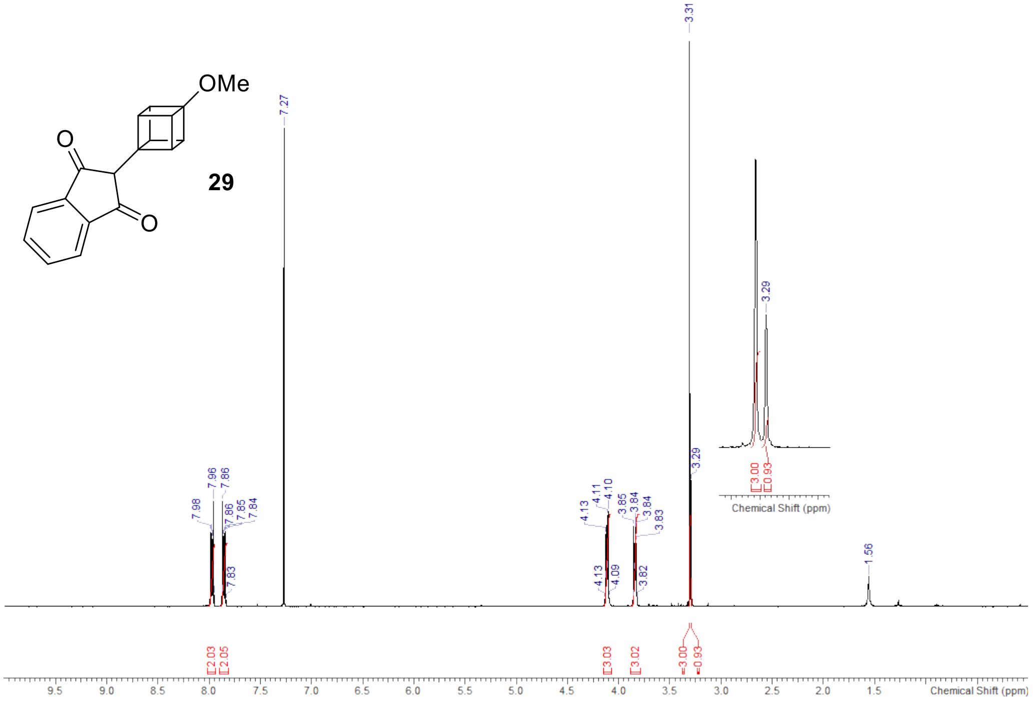

9.17.2 ^{13}C NMR (400 MHz, $CDCl_3$)

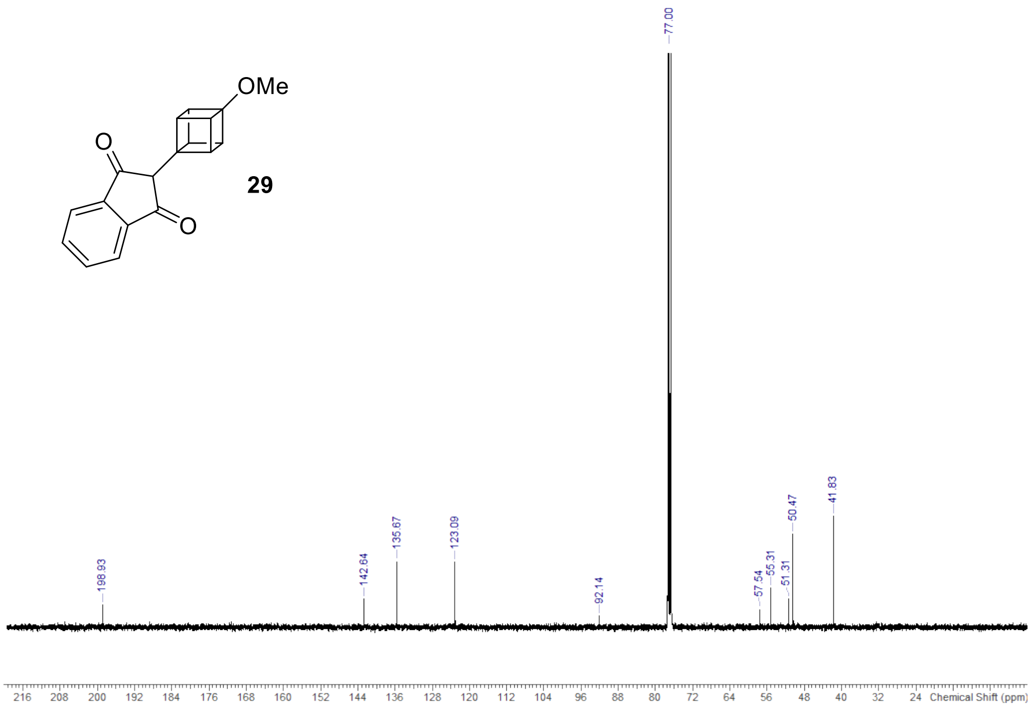

9.18 *tert*-Butyl 4-methyl cubanedicarboxylate (S1)

9.18.1 ^{1}H NMR (400 MHz, $CDCl_3$)

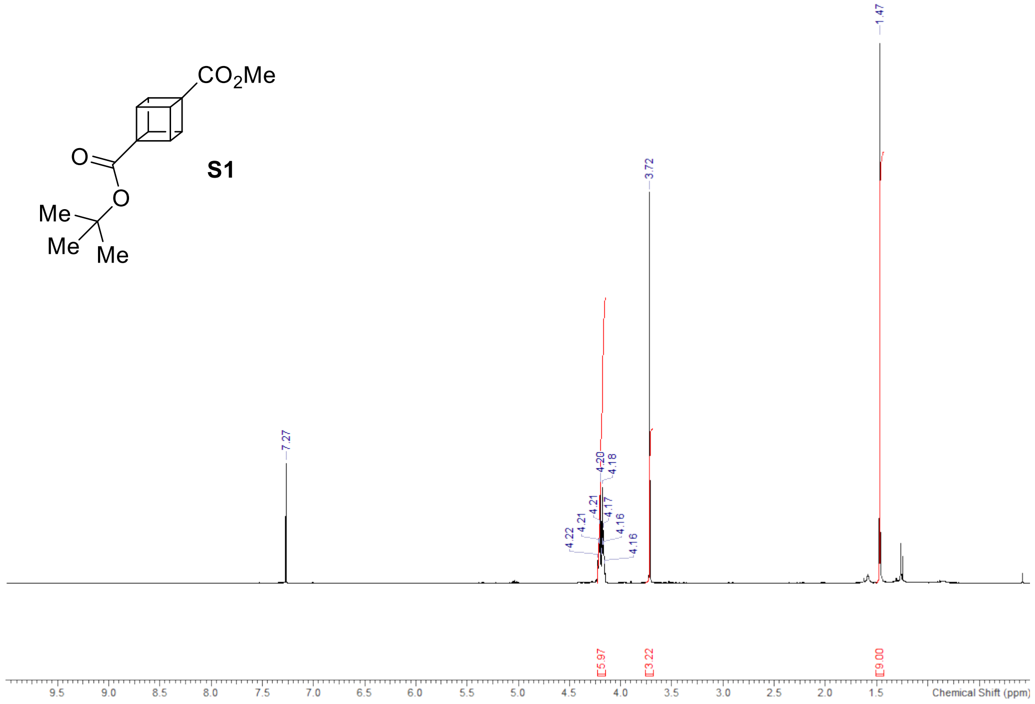

9.18.2 ^{13}C NMR (101 MHz, $CDCl_3$)

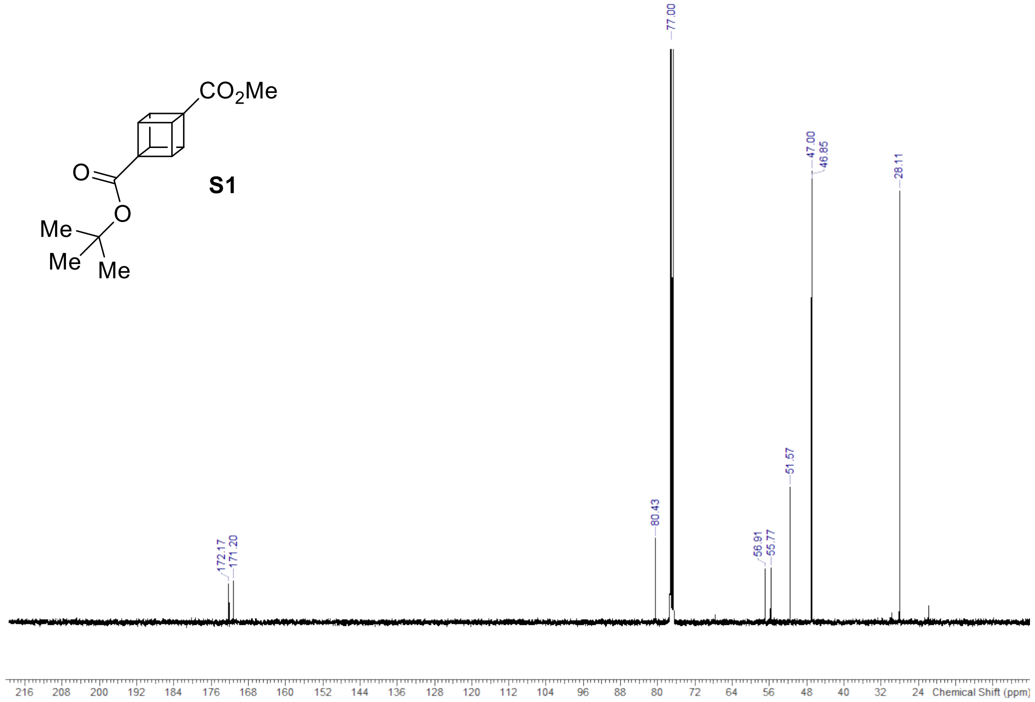

9.19 Methyl 4-hydroxymethyl-1-cubanecarboxylate (S2)

9.20 ^{1}H NMR (400 MHz, $CDCl_3$)

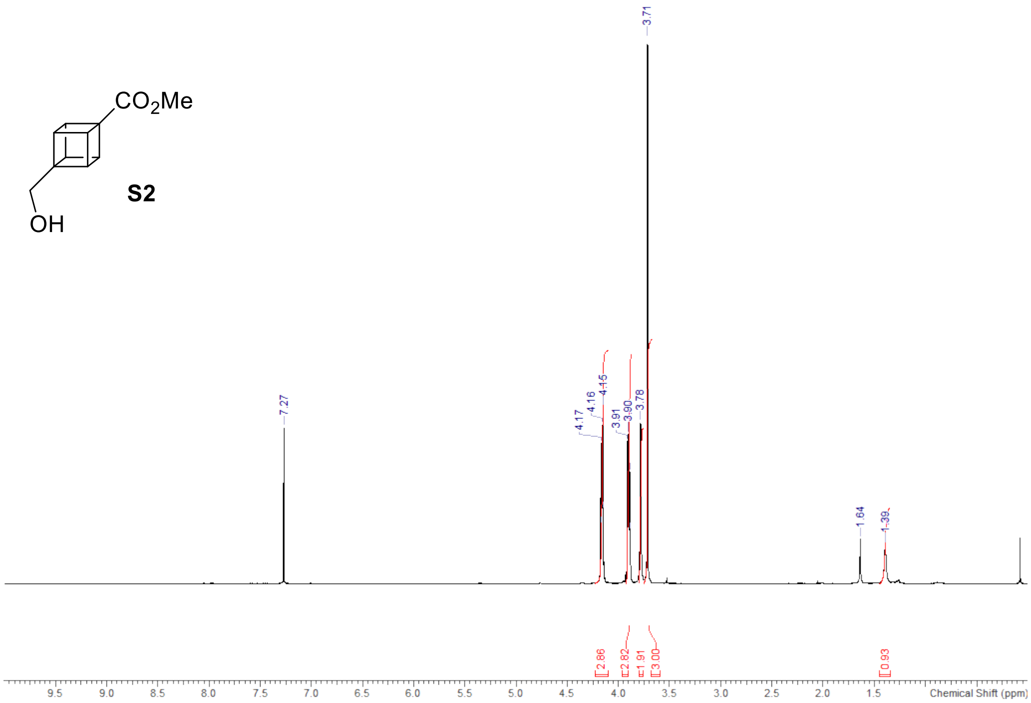

9.20.1 ^{13}C NMR (101 MHz, $CDCl_3$)

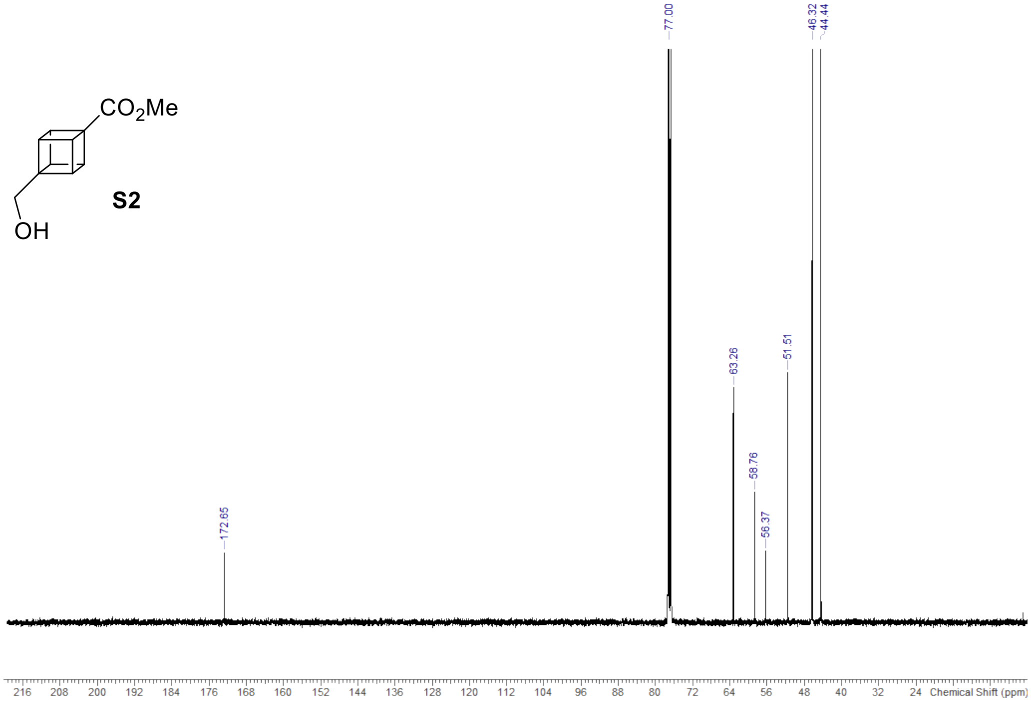

9.21 Methyl 4-benzyloxymethyl-1-cubanecarboxylate (S3)

9.21.1 ^{1}H NMR (400 MHz, $CDCl_3$)

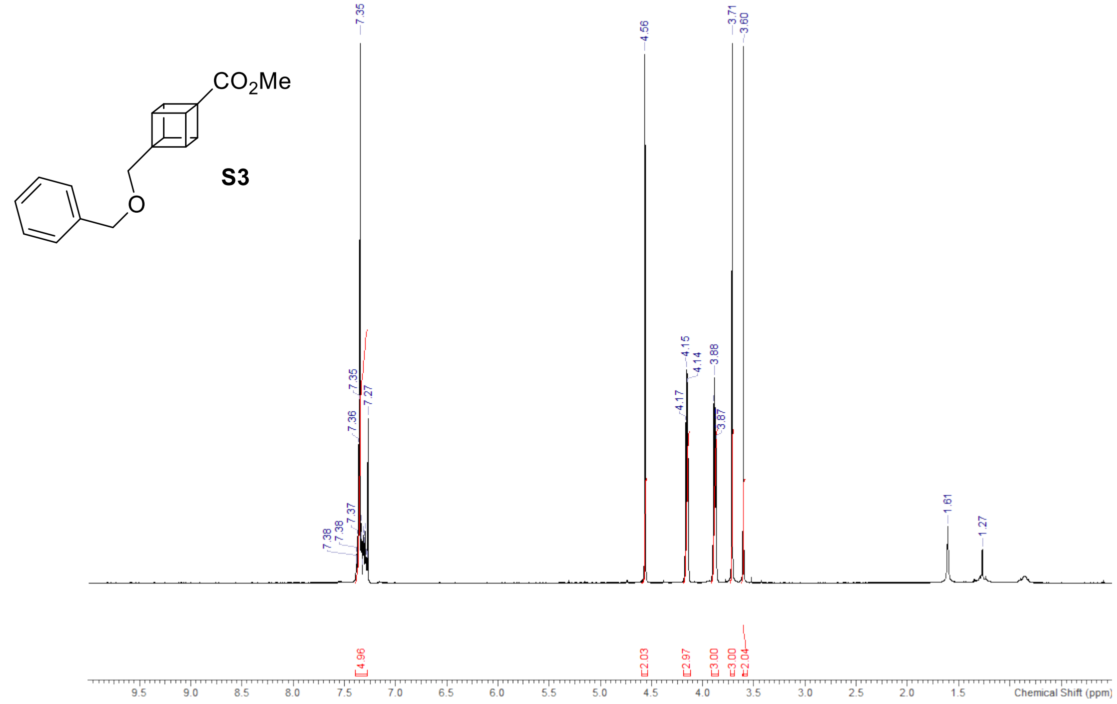

9.21.2 ^{13}C NMR (101 MHz, $CDCl_3$)

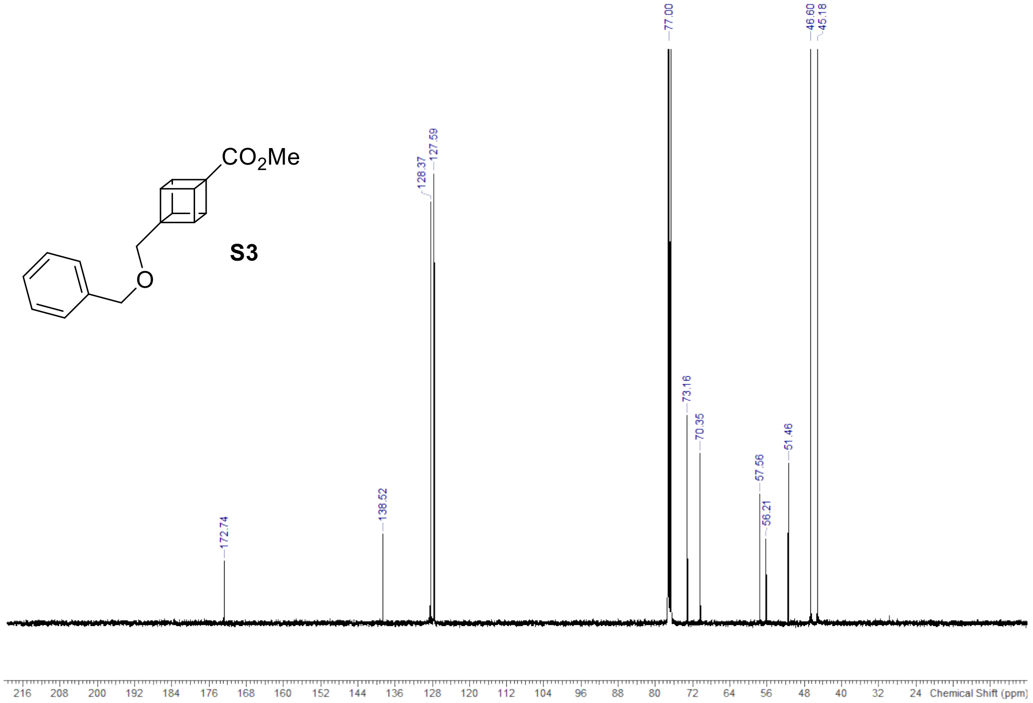

9.22 Methyl 4-((*tert*-butoxycarbonyl)amino)cubane-1-carboxylate (S4)

9.22.1 ^{1}H NMR (500 MHz, $CDCl_3$)

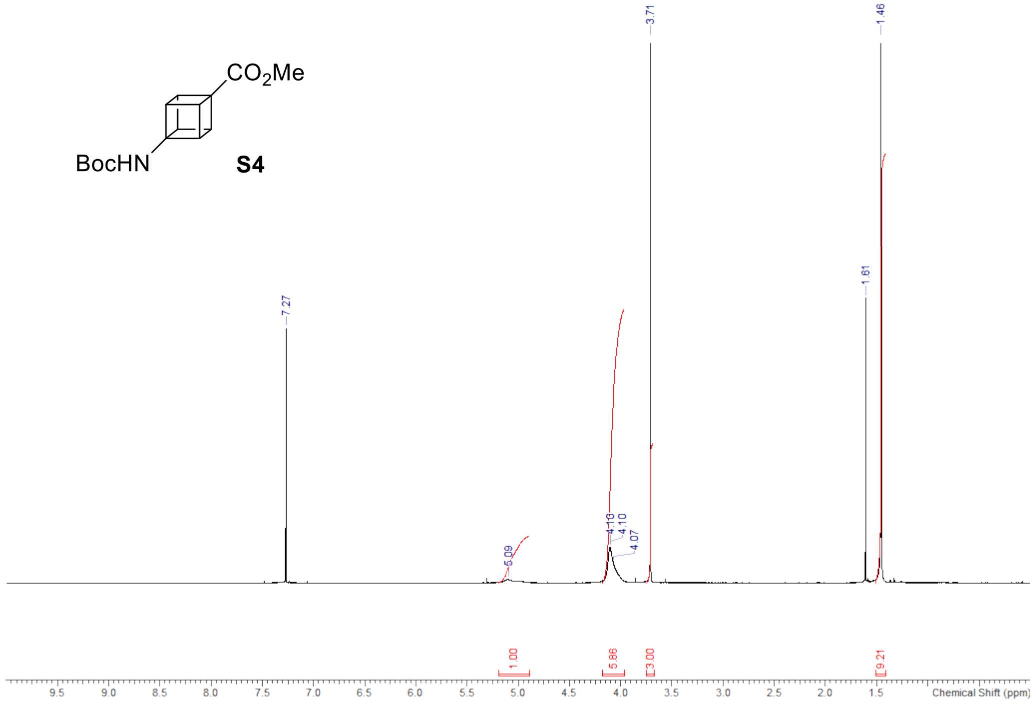

9.22.2 ^{13}C NMR (126 MHz, $CDCl_3$)

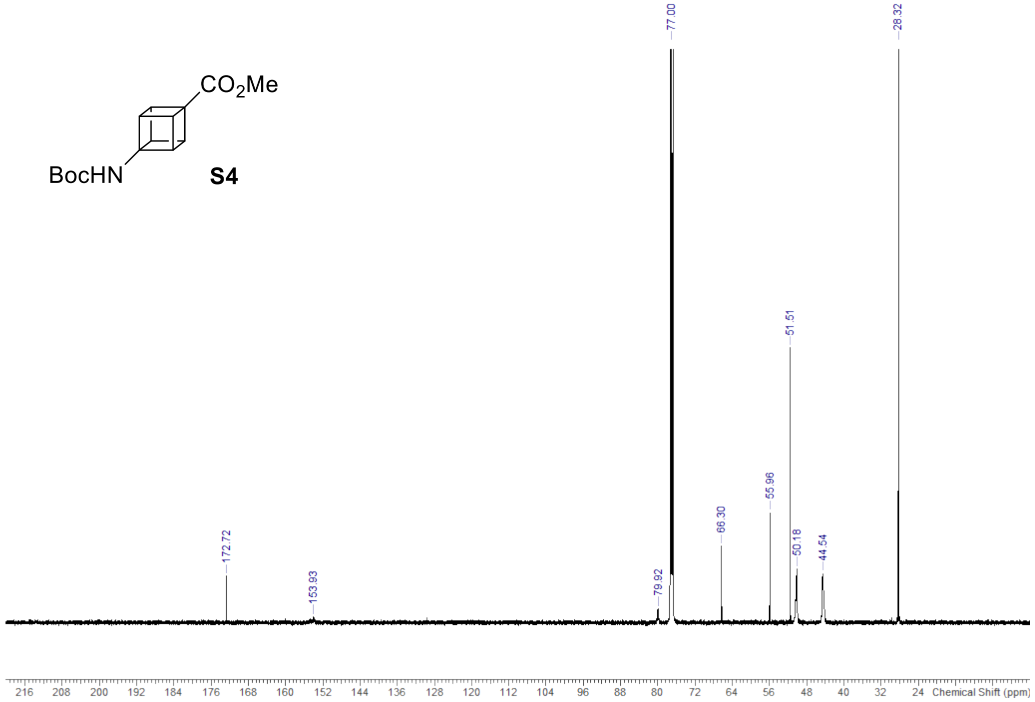

9.23 Methyl 4-(piperidine-1-carbonyl)cubane-1-carboxylate (S5)

9.23.1 ^{1}H NMR (400 MHz, CDCl$_3$)

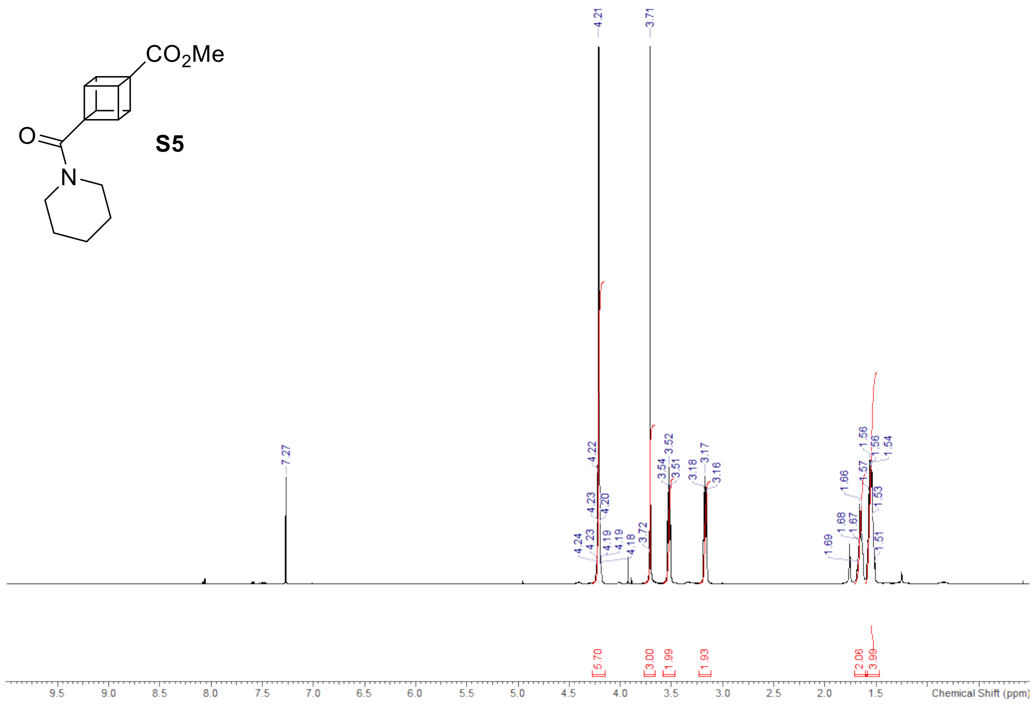

9.23.2 ^{13}C NMR (101 MHz, CDCl$_3$)

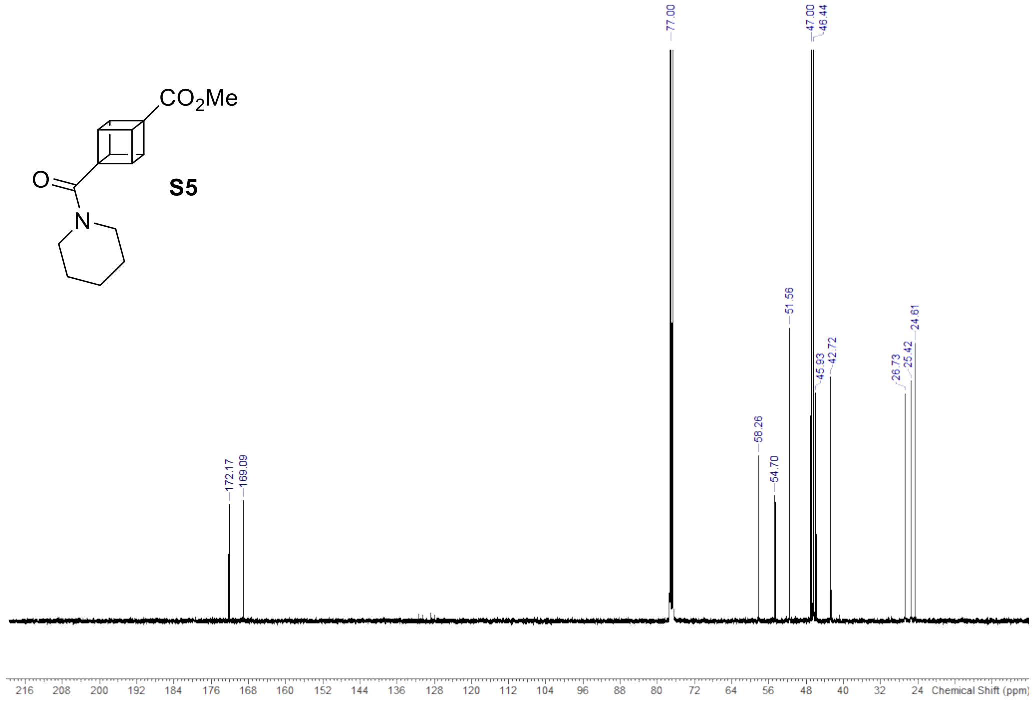

9.24 Methyl 4-(diiethylcarbamoyl)cubane-1-carboxylate (S6)

9.24.1 ^{1}H NMR (400 MHz, $CDCl_3$)

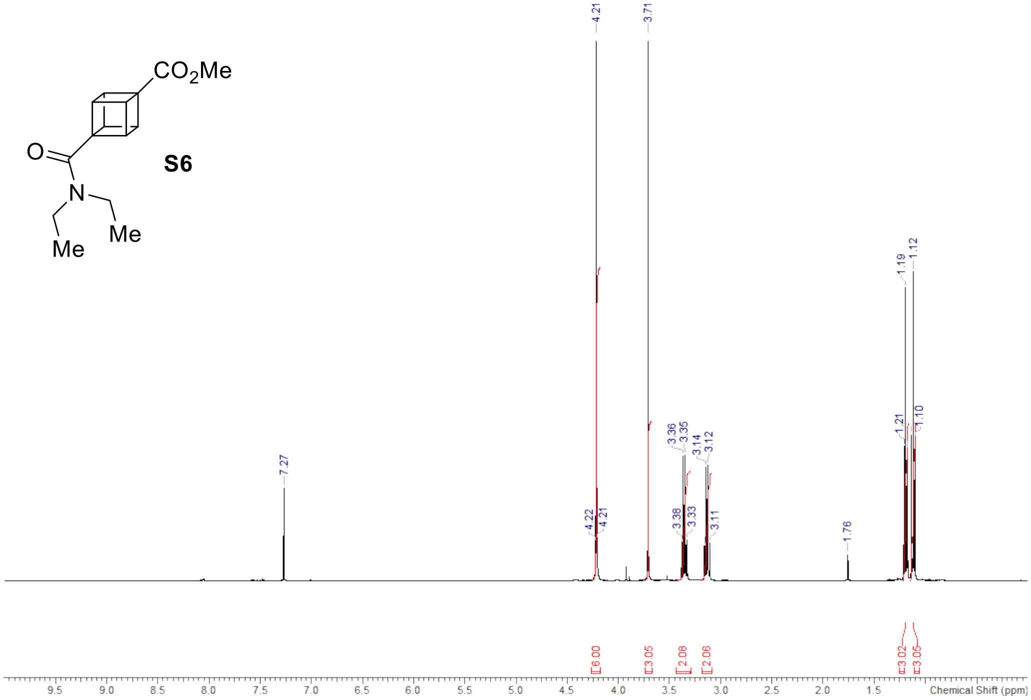

9.24.2 ^{13}C NMR (101 MHz, $CDCl_3$)

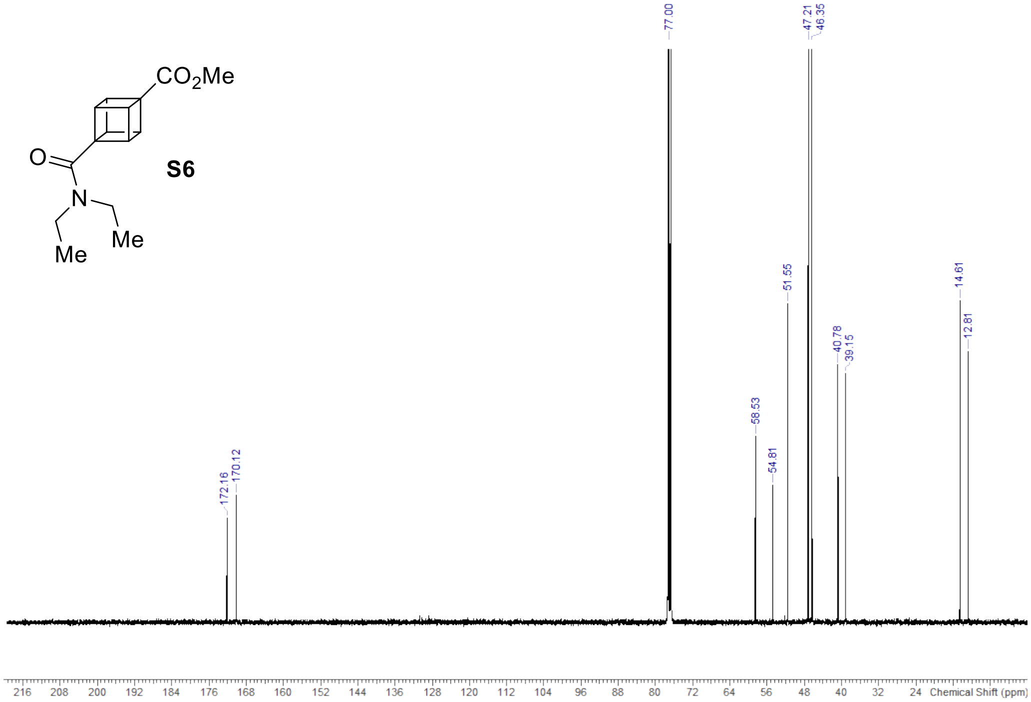

9.25 Methyl 4-(diisopropylcarbamoyl) cubane-1-carboxylate (S7)

9.25.1 ^{1}H NMR (400 MHz, $CDCl_3$)

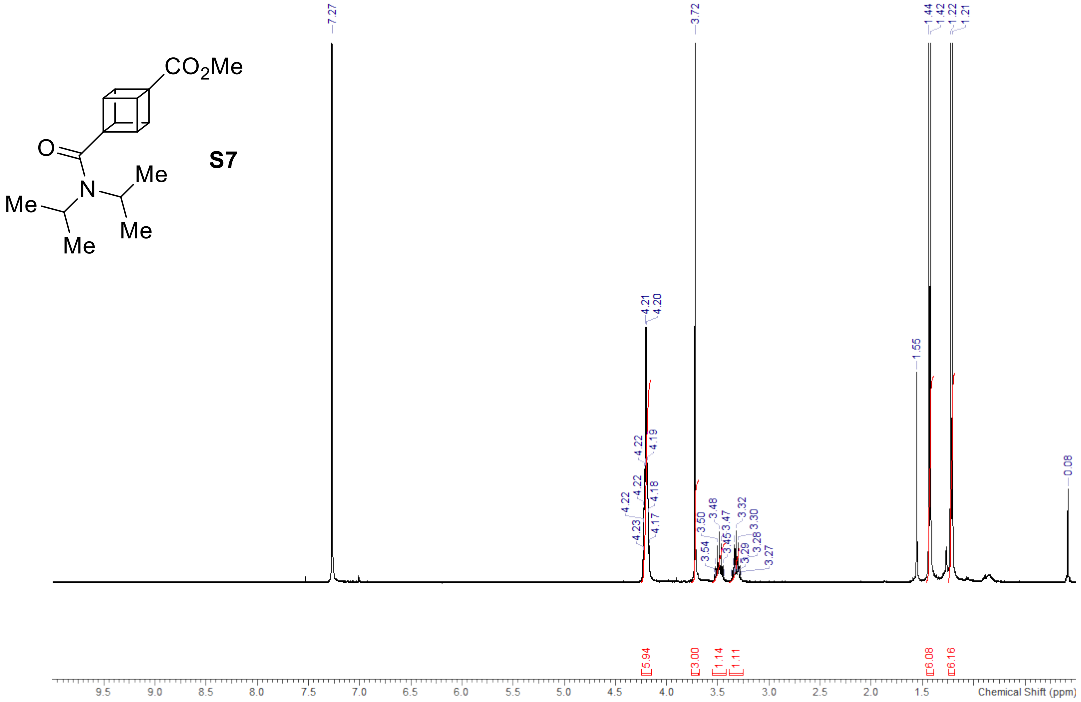

9.25.2 ^{13}C NMR (101 MHz, $CDCl_3$)

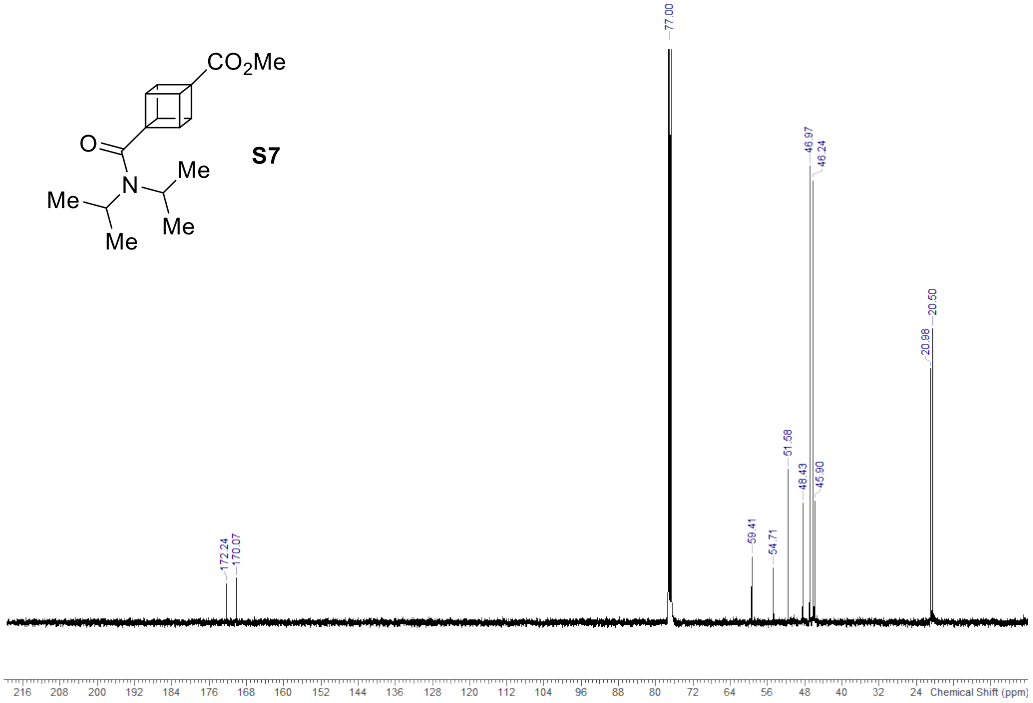

9.26 Methyl 4-(morpholine-4-carbonyl)cubane-1-carboxylate (S8)

9.26.1 ^{1}H NMR (400 MHz, $CDCl_3$)

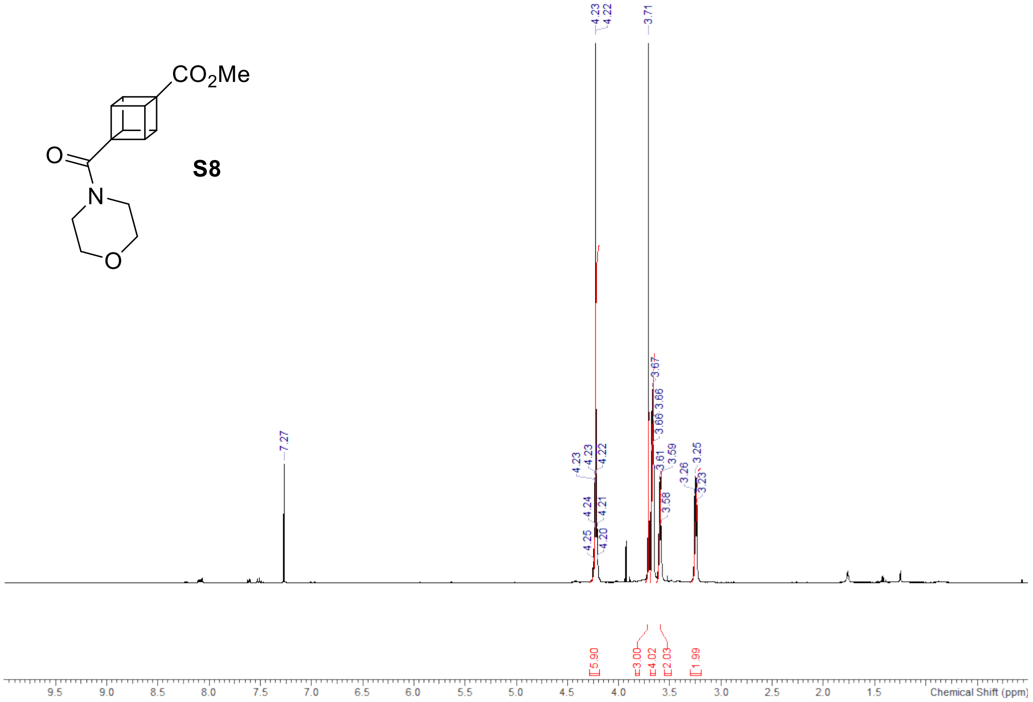

9.26.2 ^{13}C NMR (101 MHz, $CDCl_3$)

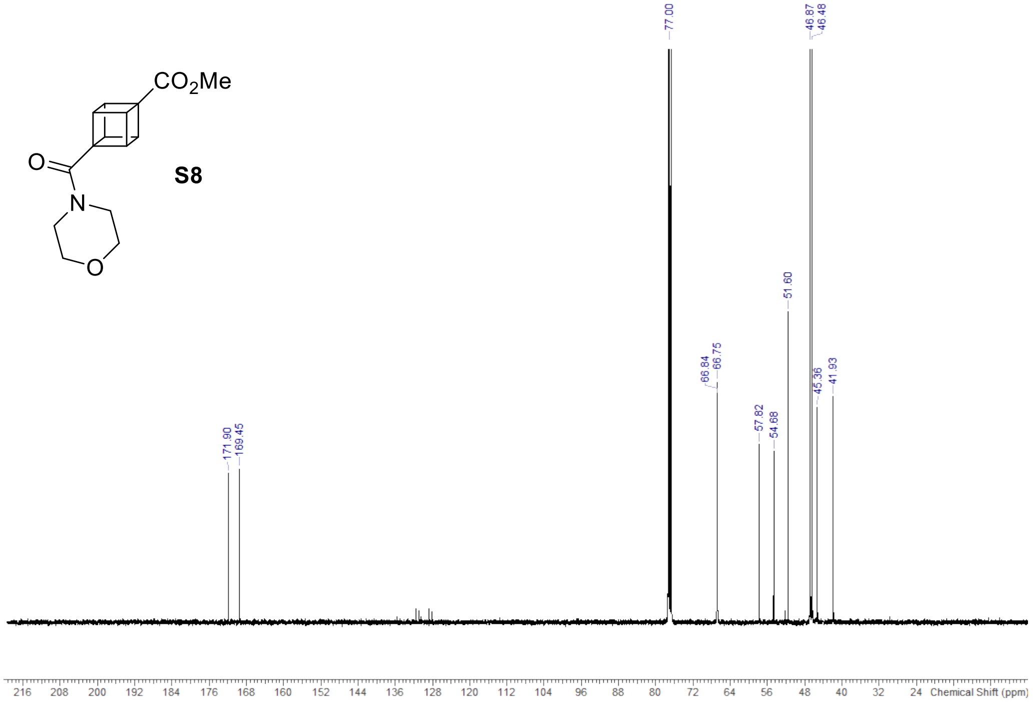

9.27 4-*tert*-Butoxycarbonyl-1-cubanecarboxylic acid (S9)

9.27.1 ^{1}H NMR (400 MHz, $CDCl_3$)

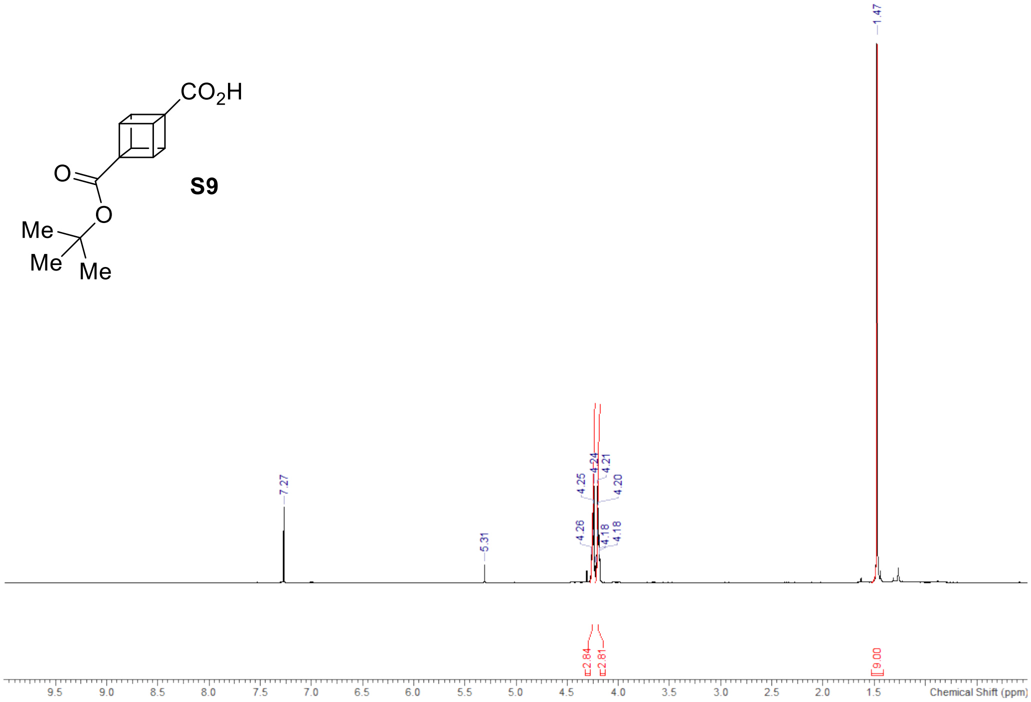

9.27.2 ^{13}C NMR (101 MHz, $CDCl_3$)

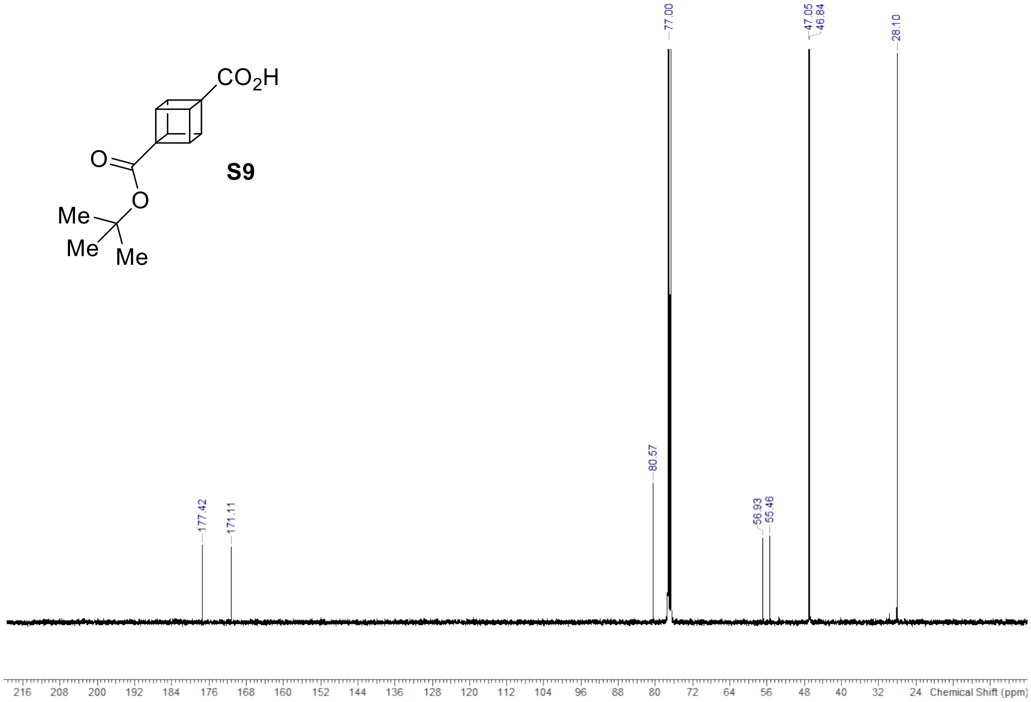

9.28 4-Hydroxymethyl-1-cubanecarboxylic acid (S10)

9.28.1 ^{1}H NMR (400 MHz, CD_3OD)

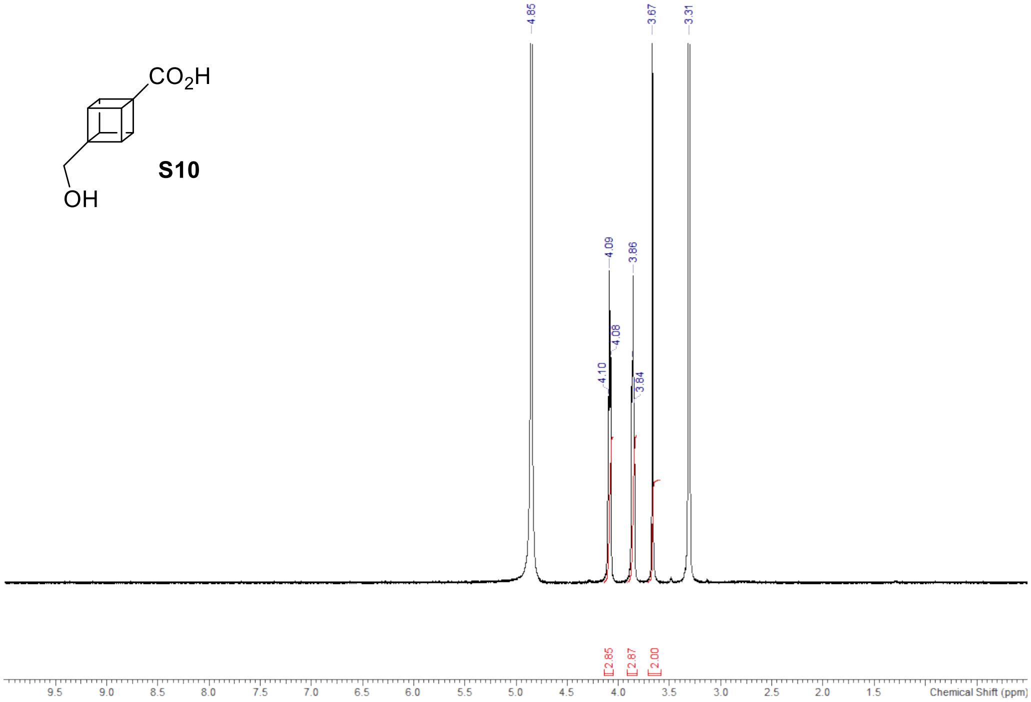

9.28.2 ^{13}C NMR (101 MHz, CD_3OD)

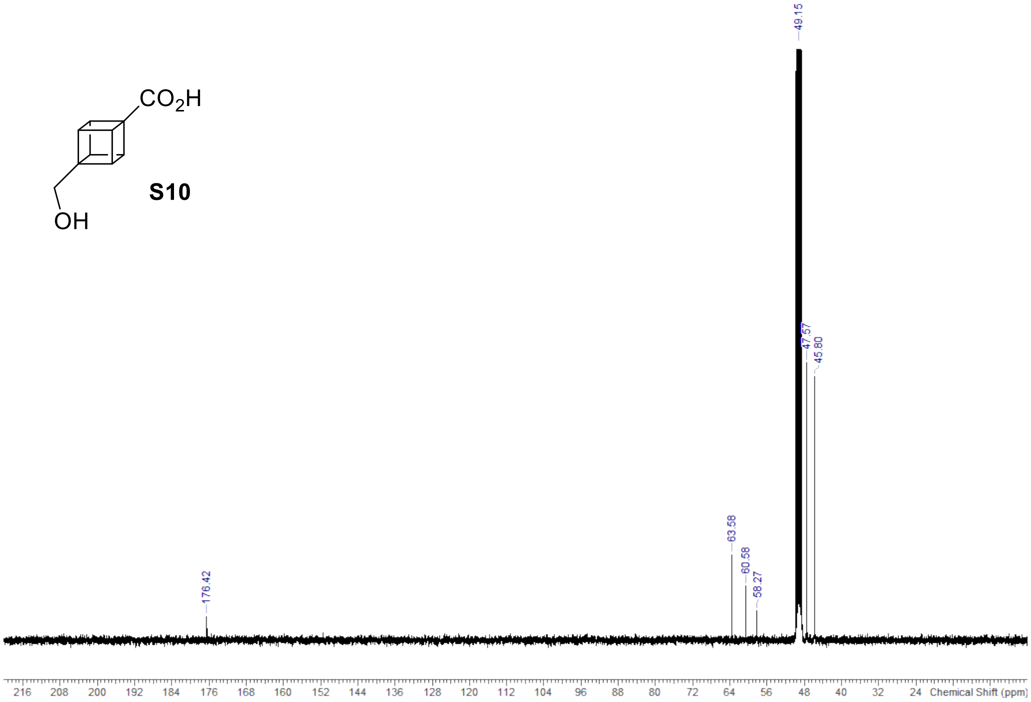

9.29 Methyl 4-benzyloxy-1-cubanecarboxylic acid (S11)

9.29.1 ^{1}H NMR (400 MHz, $CDCl_3$)

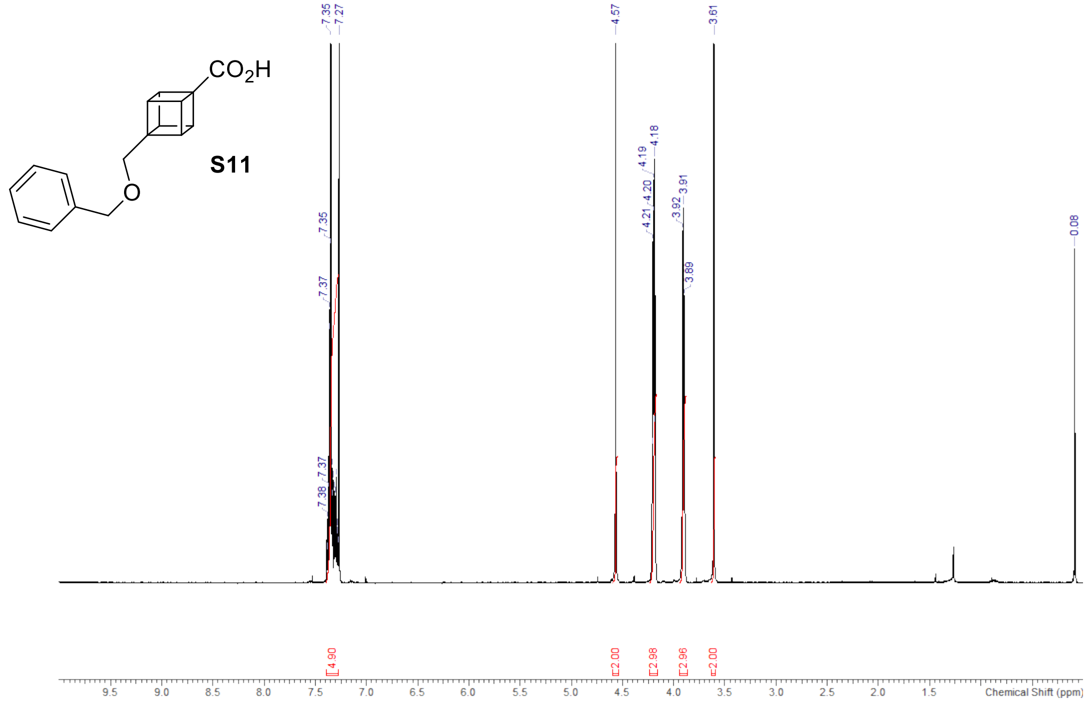

9.29.2 ^{13}C NMR (101 MHz, $CDCl_3$)

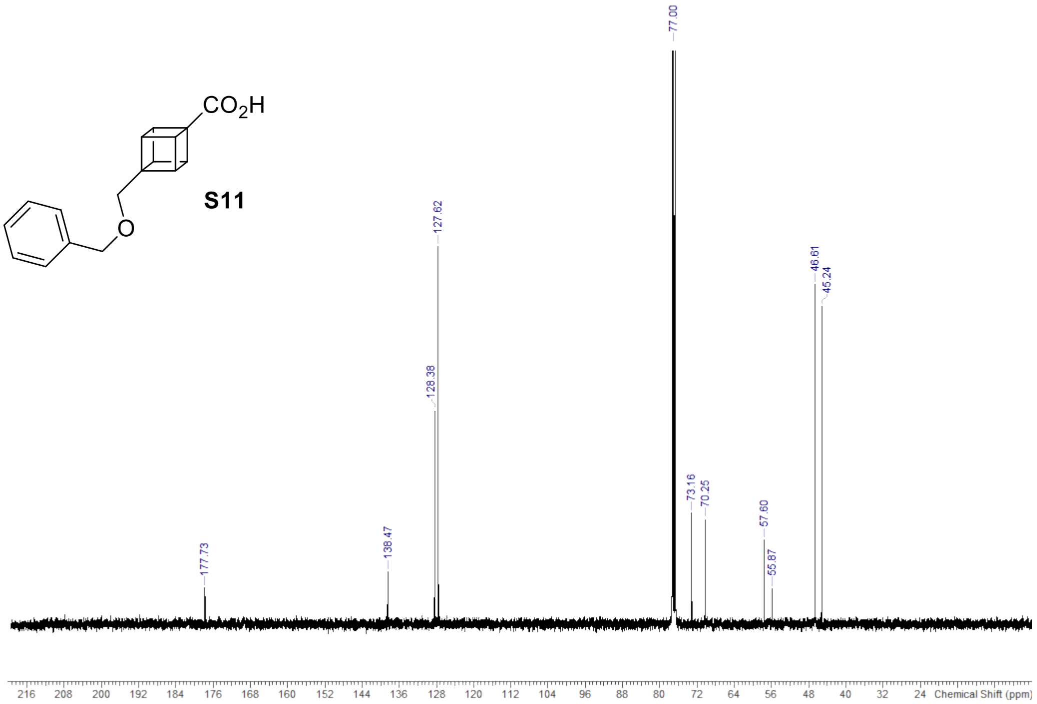

9.30 4-((*tert*-Butoxycarbonyl)amino)cubane-1-carboxylic acid (S12)

9.30.1 ^{1}H NMR (500 MHz, CD_3OD)

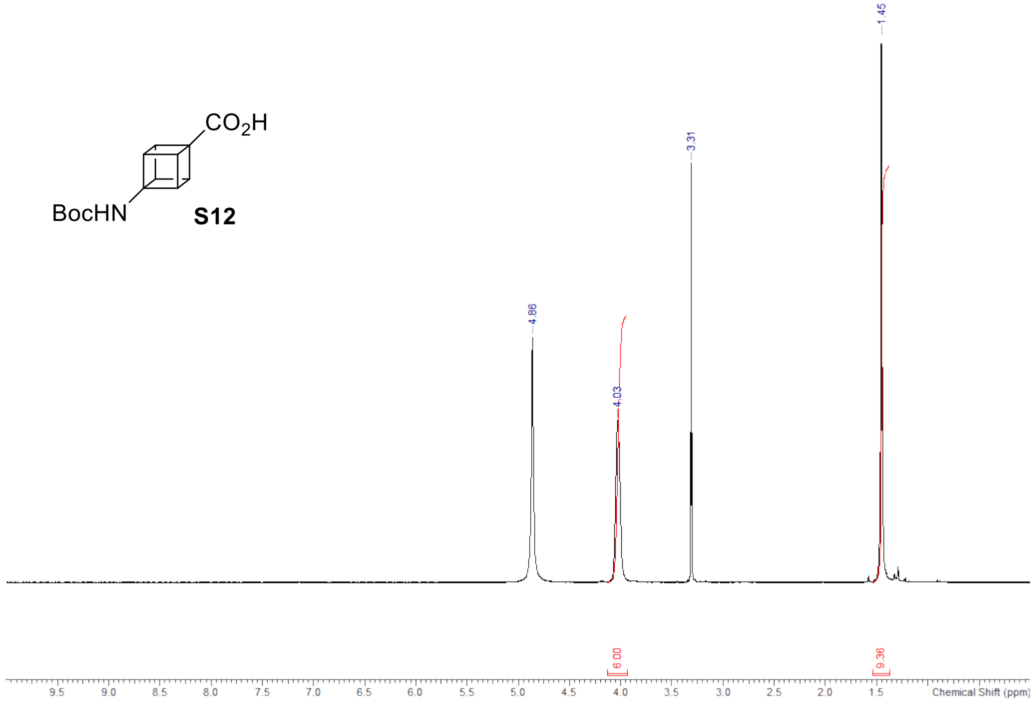

9.30.2 ^{13}C NMR (126 MHz, CD_3OD)

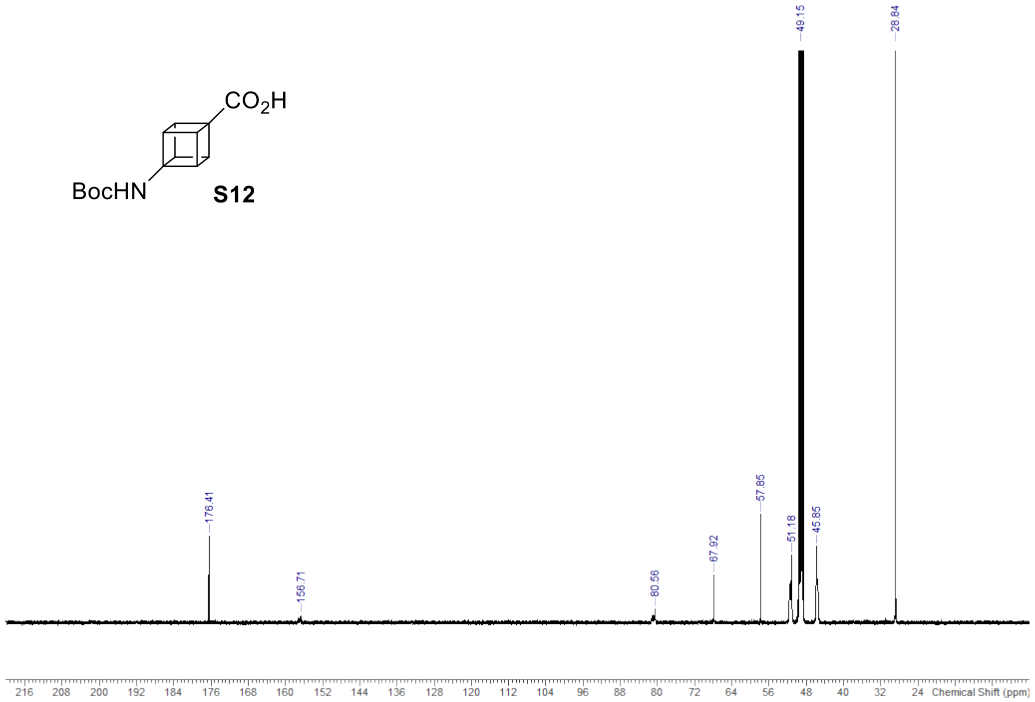

9.31 4-(Piperidine-1-carbonyl)cubane-1-carboxylic acid (S13)

9.31.1 ^{1}H NMR (400 MHz, $CDCl_3$)

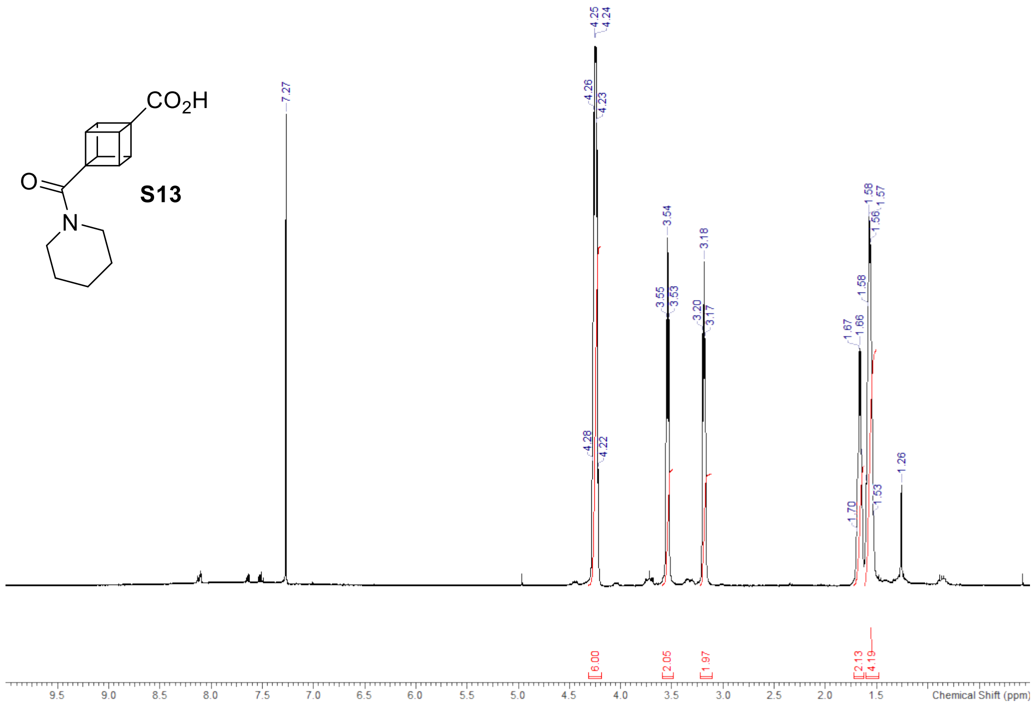

9.31.2 ^{13}C NMR (101 MHz, $CDCl_3$)

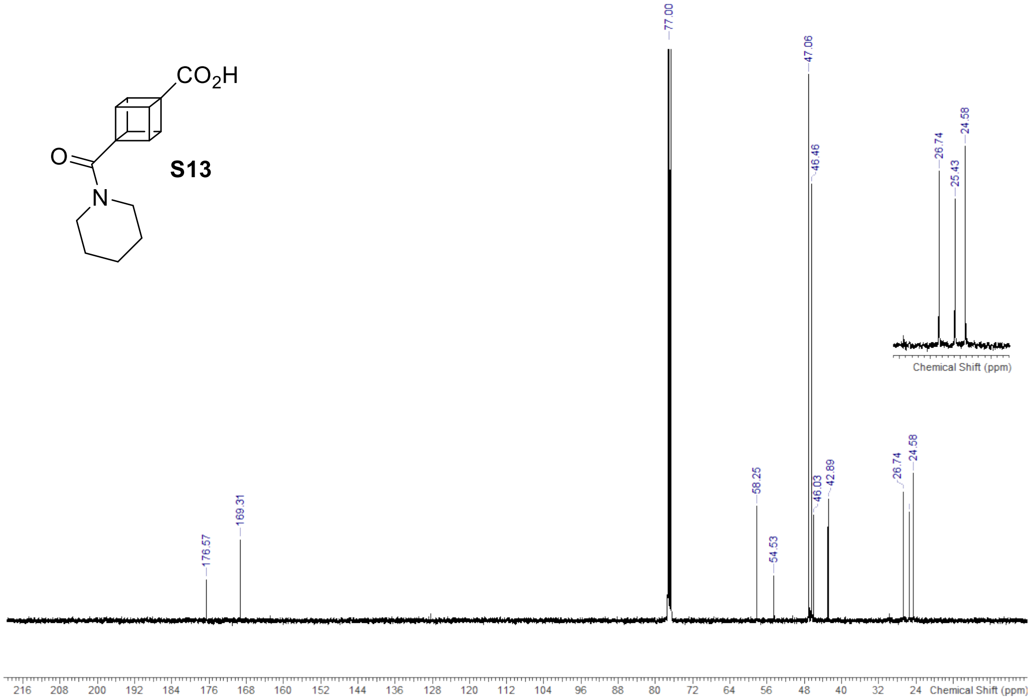

9.32 4-(Diethylcarbamoyl)cubane-1-carboxylic acid (S14)

9.32.1 ^{1}H NMR (400 MHz, $CDCl_3$)

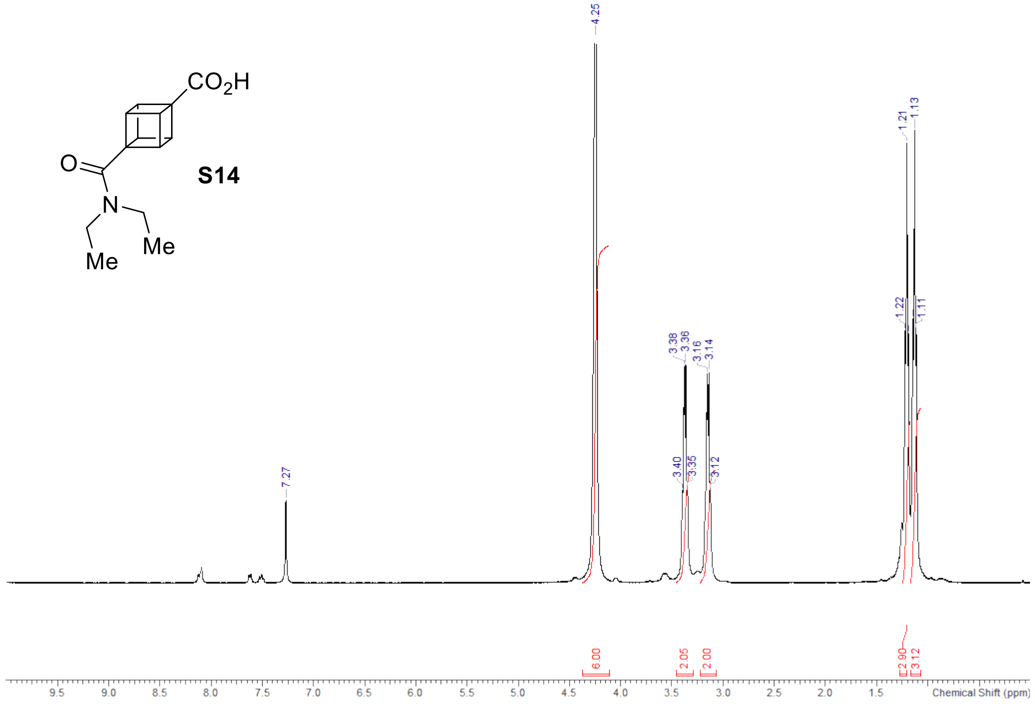

9.32.2 ^{13}C NMR (101 MHz, $CDCl_3$)

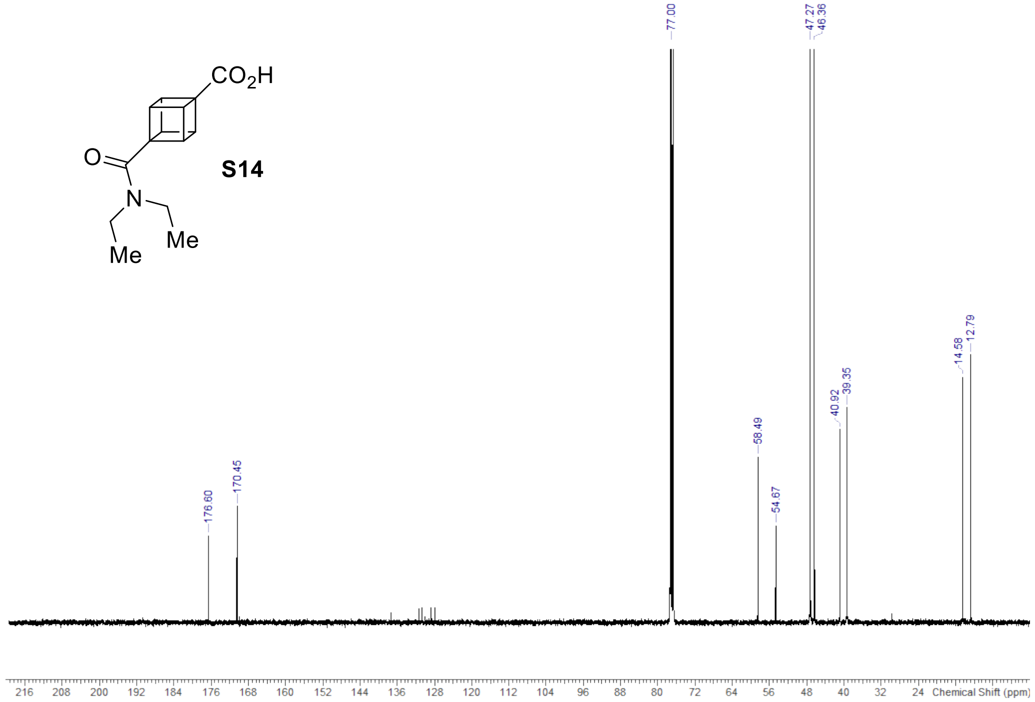

9.33 4-(Diisopropylcarbamoyl)cubane-1-carboxylic acid (S15)

9.33.1 ^{1}H NMR (400 MHz, $CDCl_3$)

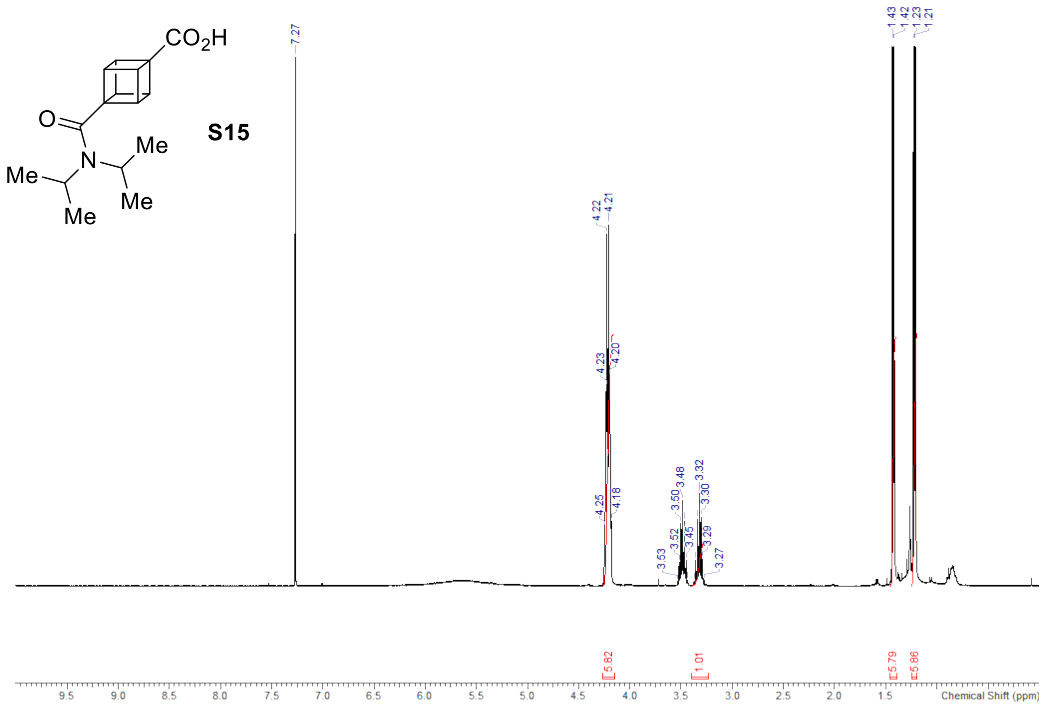

9.33.2 ^{13}C NMR (101 MHz, $CDCl_3$)

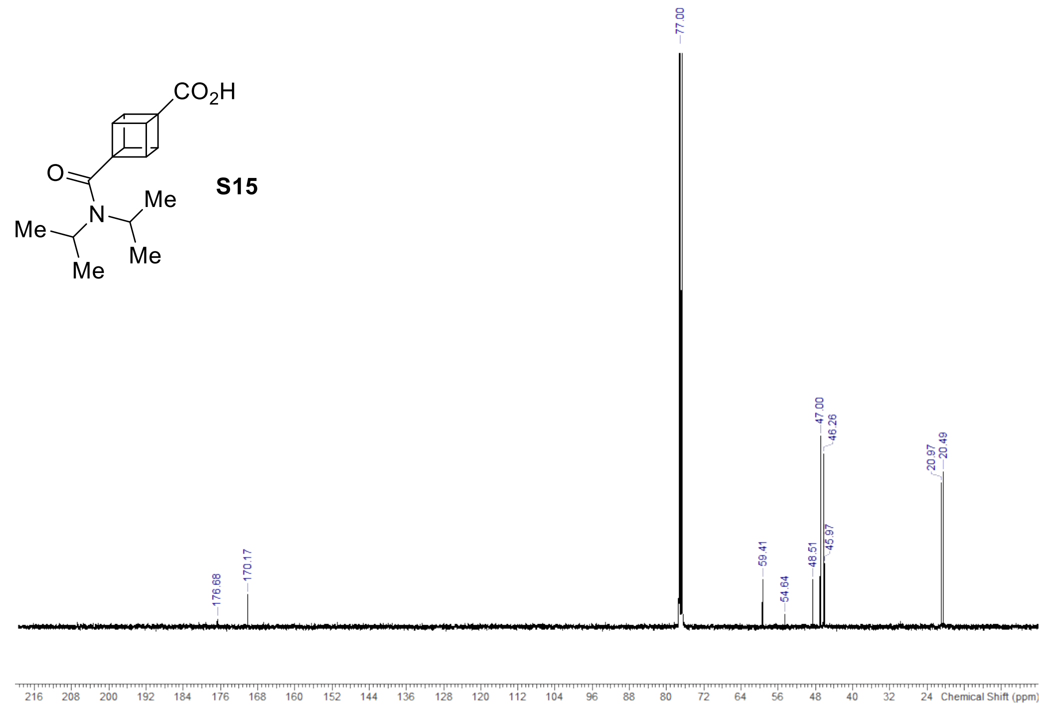

9.34 4-(Morpholine-4-carbonyl)cubane-1-carboxylic acid (S16)

9.34.1 ^{1}H NMR (400 MHz, $CDCl_3$)

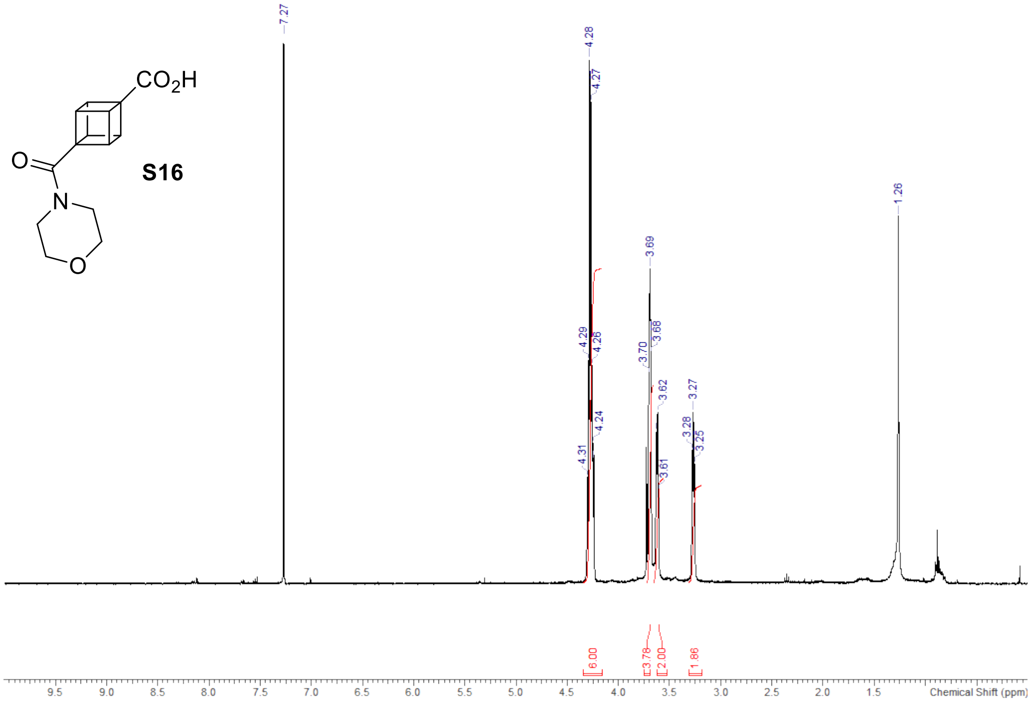

9.34.2 ^{13}C NMR (101 MHz, $CDCl_3$)

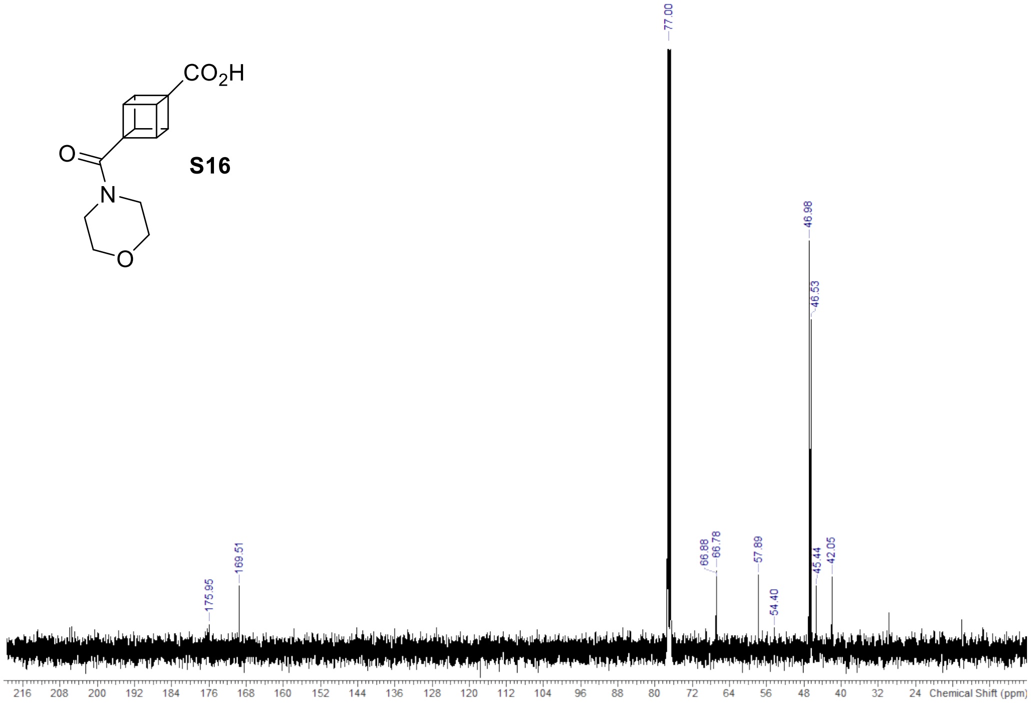

10 X-Ray Crystallographic Data

10.1 Methyl 4-methoxy-1-cubanecarboxylate [CCDC: 1905443 2019sot0002_K1_100K] 7

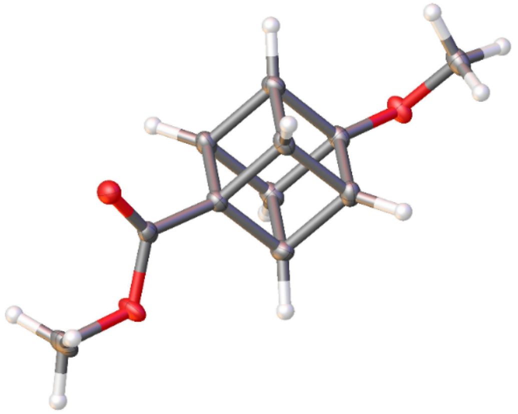

Figure S25: Structure of **7** with thermal ellipsoids drawn at the 50% probability level.

Experimental: Single clear colourless prism-shaped crystals of **DC_8620_53** crystallised from Et_2O by slow evaporation. A suitable crystal 0.25×0.20×0.10 mm^3 was selected and mounted on a MITIGEN holder silicon oil on a Rigaku AFC12 FRE-HF diffractometer.[i] The crystal was kept at a steady T = 100(2) K during data collection. The structure was solved with the ShelXT[ii] structure solution program using the Intrinsic Phasing solution method and by using Olex2[iii] as the graphical interface. The model was refined with version 2016/6 of ShelXL[iv] using Least Squares minimisation.

[i] CrysAlisPro Software System, Rigaku Oxford Diffraction, (2018).
[ii] Sheldrick, G. M. *Acta Cryst.* **2015**, *A71*, 3-8.
[iii] Dolomanov, O. V., Bourhis, L. J., R.J. Gildea, R. J., and J.A.K. Howard, J. A. K. and H. Puschmann, H., *J. Appl. Cryst.* **2009**, *42*, 339-341.
[iv] Sheldrick, G. M., *Acta Cryst.*, **2015**, *C27*, 3-8.

Table SX: Crystal data of 7

Formula	$C_{11}H_{12}O_3$
$D_{calc.}$/ g cm^{-3}	1.392
μ/mm^{-1}	0.101
Formula Weight	192.21
Colour	clear colourless
Shape	prism
Size/mm^3	0.25×0.20×0.10
T/K	100(2)
Crystal System	monoclinic
Space Group	$P2_1/c$
a/Å	7.2351(2)
b/Å	8.0399(2)
c/Å	31.7327(5)
$\alpha/^\circ$	90
$\beta/^\circ$	96.243(2)
$\gamma/^\circ$	90
V/Å^3	1834.93(7)
Z	8
Z'	2
Wavelength/Å	0.71073
Radiation type	MoK_α
$\Theta_{min}/^\circ$	2.982
$\Theta_{max}/^\circ$	28.499
Measured Refl.	40539
Independent Refl.	4658
Reflections with I > 2(I)	4415
R_{int}	0.0480
Parameters	257
Restraints	0
Largest Peak	0.404
Deepest Hole	–0.224
GooF	1.276
wR_2 (all data)	0.1408
wR_2	0.1379
R_1 (all data)	0.0707
R_1	0.0655

10.2 Methyl 4-(1,1,1,3,3,3-hexafluoroisopropoxy)-1-cubanecarboxylate [CCDC: 1889723 2018sot0040_K1_100K] 15

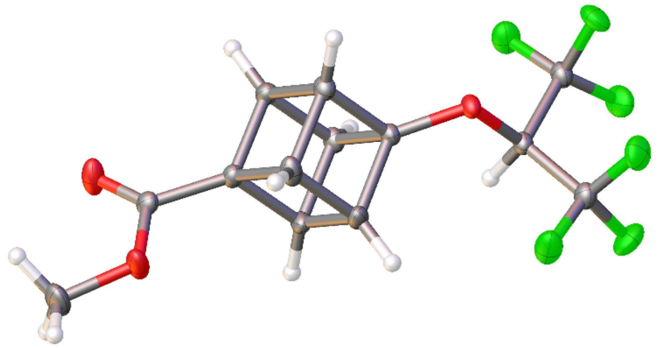

Figure S26: Structure of **15** with thermal ellipsoids drawn at the 50% probability level.

Experimental: Single clear colourless prism-shaped crystals of **DC_8507_67** crystallised from HFIP by slow evaporation. A suitable crystal 0.46×0.28×0.10 mm^3 was selected and mounted on a MITIGEN holder silicon oil on a Rigaku AFC12 FRE-HF diffractometer.[i] The crystal was kept at a steady T = 100(2) K during data collection. The structure was solved with the ShelXT[ii] structure solution program using the Intrinsic Phasing solution method and by using Olex2[iii] as the graphical interface. The model was refined with version 2016/6 of ShelXL[iv] using Least Squares minimisation.

Table SX: Crystal data of 15

Formula	$C_{13}H_{10}F_6O_3$
$D_{calc.}$/ g cm^{-3}	1.738
μ/mm^{-1}	0.179
Formula Weight	328.21
Colour	clear colourless
Shape	prism
Size/mm^3	0.46×0.28×0.10
T/K	100(2)
Crystal System	monoclinic
Space Group	$P2_1/n$
a/Å	11.2408(3)
b/Å	9.7652(2)
c/Å	12.5959(3)
$\alpha/^\circ$	90
$\beta/^\circ$	114.902(3)
$\gamma/^\circ$	90
V/Å^3	1254.09(6)
Z	4
Z'	1
Wavelength/Å	0.71073
Radiation type	MoK_α
$\Theta_{min}/^\circ$	2.888
$\Theta_{max}/^\circ$	28.500
Measured Refl.	18889
Independent Refl.	3169
Reflections with I > 2(I)	2860
R_{int}	0.0488
Parameters	200
Restraints	0
Largest Peak	0.375
Deepest Hole	–0.272
GooF	1.075
wR_2 (all data)	0.1007
wR_2	0.0969
R_1 (all data)	0.0471
R_1	0.0416

10.3 Methyl 4-ethoxy-1-cubanecarboxylate [CCDC: 1919701 2019sot0009_K1_100K] 12

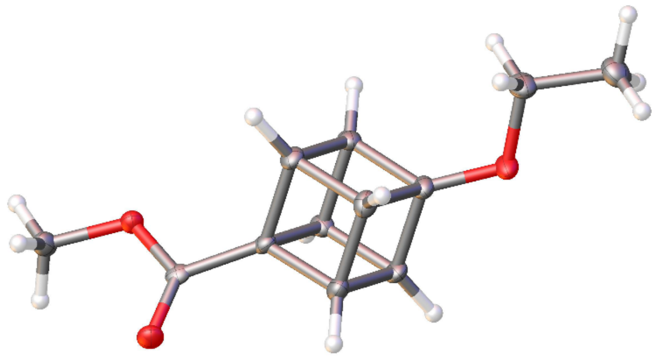

Figure S27: Structure of **12** with thermal ellipsoids drawn at the 50% probability level.

Experimental. Single clear colourless plate-shaped crystals of **DC_8620_76** crystallised from hexane by slow evaporation. A suitable crystal 0.40×0.21×0.08 mm^3 was selected and mounted on a MITIGEN holder with silicon oil on a Rigaku AFC12 FRE-HF diffractometer.[i] The crystal was kept at a steady T = 100(2) K during data collection. The structure was solved with the **ShelXT**[ii] structure solution program using the Intrinsic Phasing solution method and by using **Olex2**[iii] as the graphical interface. The model was refined with version 2016/6 of **ShelXL**[iv] using Least Squares minimisation.

Table 2: Crystal data of 12

Formula	$C_{12}H_{14}O_3$
$D_{calc.}$/ g cm^{-3}	1.334
μ/mm^{-1}	0.095
Formula Weight	206.23
Colour	clear colourless
Shape	plate
Size/mm^3	0.40×0.21×0.08
T/K	100(2)
Crystal System	monoclinic
Space Group	$P2_1/n$
a/Å	5.7615(2)
b/Å	28.2305(8)
c/Å	6.3185(2)
α/°	90
β/°	92.235(3)
γ/°	90
V/Å^3	1026.92(6)
Z	4
Z'	1
Wavelength/Å	0.71073
Radiation type	MoK$_a$
Θ_{min}/°	3.306
Θ_{max}/°	28.493
Measured Refl.	15175
Independent Refl.	2609
Reflections with I > 2(I)	2434
R_{int}	0.0513
Parameters	138
Restraints	0
Largest Peak	0.439
Deepest Hole	–0.264
GooF	1.251
wR_2 (all data)	0.1566
wR_2	0.1531
R_1 (all data)	0.0907
R_1	0.0823

10.4 Methyl 4-methoxy(d_3) cubanecarboxylate (11) [CCDC: 1919978 2019sot0008_R1_100K]

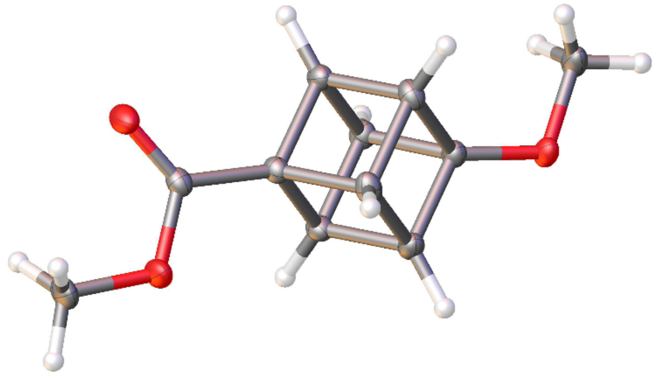

Figure S28: Structure of **11** with thermal ellipsoids drawn at the 50% probability level.

Experimental. Single clear colourless prism-shaped crystals of **DC_8620_78** crystallised from hexane by slow evaporation. A suitable crystal 0.25×0.15×0.10 mm^3 was selected and mounted on a MITIGEN holder with silicon oil on a Rigaku AFC12 FRE-VHF diffractometer.[i] The crystal was kept at a steady T = 100(2) K during data collection. The structure was solved with the **ShelXS**[ii] structure solution program using the Direct Methods solution method and by using **Olex2**[iii] as the graphical interface. The model was refined with version 2016/6 of **ShelXL**[iv] using Least Squares minimisation.

Table 3: Crystal data of 11.

Formula	$C_{11}H_9D_3O_3$
$D_{calc.}$/ g cm^{-3}	1.417
μ/mm^{-1}	0.101
Formula Weight	195.22
Colour	clear colourless
Shape	prism
Size/mm^3	0.25×0.15×0.10
T/K	100(2)
Crystal System	monoclinic
Space Group	$P2_1/c$
a/Å	7.2266(2)
b/Å	8.0387(2)
c/Å	31.6932(6)
α/°	90
β/°	96.282(2)
γ/°	90
V/Å^3	1830.08(8)
Z	8
Z'	2
Wavelength/Å	0.71073
Radiation type	MoK$_a$
Θ_{min}/°	1.939
Θ_{max}/°	31.816
Measured Refl.	48674
Independent Refl.	5842
Reflections with I > 2(I)	5373
R_{int}	0.0889
Parameters	258
Restraints	0
Largest Peak	0.313
Deepest Hole	–0.313
GooF	1.097
wR_2 (all data)	0.1383
wR_2	0.1364
R_1 (all data)	0.0514
R_1	0.0485

11 References

(1). Green, R. A.; Brown, R. C. D.; Pletcher, D.; Harji, B., *Electrochem. Commun.* **2016,** *73*, 63-66.
(2). Schulz, L.; Enders, M.; Elsler, B.; Schollmeyer, D.; Dyballa, K. M.; Franke, R.; Waldvogel, S. R., *Angew. Chem., Int. Ed.* **2017,** *56*, 4877-4881.
(3). Irngartinger, H.; Strack, S.; Gredel, F.; Dreuw, A.; Della, E. W., *Eur. J. Org. Chem.* **1999,** *1999*, 1253-1257.
(4). Qin, T.; Malins, L. R.; Edwards, J. T.; Merchant, R. R.; Novak, A. J.; Zhong, J. Z.; Mills, R. B.; Yan, M.; Yuan, C.; Eastgate, M. D., *Angew. Chem., Int. Ed.* **2017,** *56*, 260-265.
(5). Ma, X.; Sloman, D. L.; Han, Y.; Bennett, D. J., *Org. Lett.* **2019**.
(6). Wlochal, J.; Davies, R. D.; Burton, J., *Org. Lett.* **2014,** *16*, 4094-4097.
(7). Nicolaou, K. C.; Yin, J.; Mandal, D.; Erande, R. D.; Klahn, P.; Jin, M.; Aujay, M.; Sandoval, J.; Gavrilyuk, J.; Vourloumis, D., *J. Am. Chem. Soc.* **2016,** *138*, 1698-1708.
(8). Eaton, P. E.; Xiong, Y.; Zhou, J. P., *J. Org. Chem.* **1992,** *57*, 4277-4281.
